# Versatile Diphosphine Chelators for Radiolabeling Peptides with ^99m^Tc and ^64^Cu

**DOI:** 10.1021/acs.inorgchem.3c00426

**Published:** 2023-03-27

**Authors:** Ingebjørg N. Hungnes, Truc Thuy Pham, Charlotte Rivas, James A. Jarvis, Rachel E. Nuttall, Saul M. Cooper, Jennifer D. Young, Philip J. Blower, Paul G. Pringle, Michelle T. Ma

**Affiliations:** † School of Biomedical Engineering and Imaging Sciences, King’s College London, Fourth Floor Lambeth Wing, St. Thomas’ Hospital, London SE1 7EH, United Kingdom; ‡ Randall Centre of Cell and Molecular Biophysics and Centre for Biomolecular Spectroscopy, King’s College London, London SE1 9RT, United Kingdom; § School of Chemistry, University of Bristol, Cantock’s Close, Bristol BS8 1TS, United Kingdom; ∥ Department of Chemistry, Imperial College London, Molecular Sciences Research Hub, London W12 0BZ, United Kingdom

## Abstract

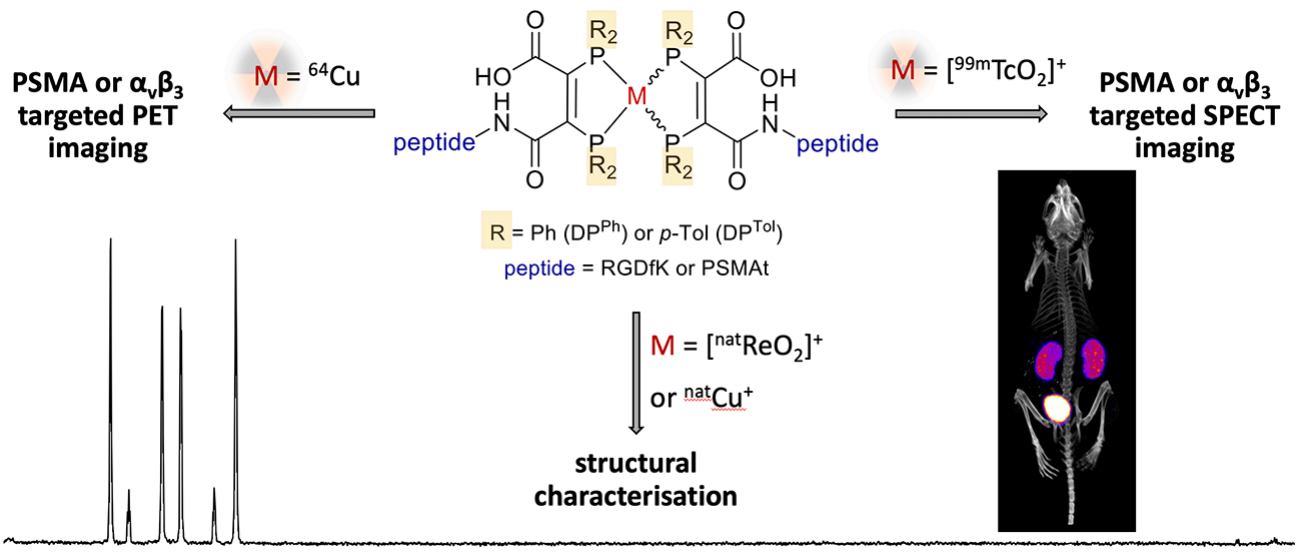

We have developed a diphosphine (DP) platform for radiolabeling peptides with ^99m^Tc and ^64^Cu for molecular SPECT and PET imaging, respectively. Two diphosphines, 2,3-bis(diphenylphosphino)maleic anhydride (DP^Ph^) and 2,3-bis(di-*p*-tolylphosphino)maleic anhydride (DP^Tol^), were each reacted with a Prostate Specific Membrane Antigen-targeted dipeptide (PSMAt) to yield the bioconjugates DP^Ph^-PSMAt and DP^Tol^-PSMAt, as well as an integrin-targeted cyclic peptide, RGD, to yield the bioconjugates DP^Ph^-RGD and DP^Tol^-RGD. Each of these DP-PSMAt conjugates formed geometric *cis*/*trans*-[MO_2_(DP^X^-PSMAt)_2_]^+^ (M = ^99m^Tc, ^99g^Tc, ^nat^Re; X = Ph, Tol) complexes when reacted with [MO_2_]^+^ motifs. Furthermore, both DP^Ph^-PSMAt and DP^Tol^-PSMAt could be formulated into kits containing reducing agent and buffer components, enabling preparation of the new radiotracers *cis*/*trans*-[^99m^TcO_2_(DP^Ph^-PSMAt)_2_]^+^ and *cis*/*trans*-[^99m^TcO_2_(DP^Tol^-PSMAt)_2_]^+^ from aqueous ^99m^TcO_4_^–^ in 81% and 88% radiochemical yield (RCY), respectively, in 5 min at 100 °C. The consistently higher RCYs observed for *cis*/*trans*-[^99m^TcO_2_(DP^Tol^-PSMAt)_2_]^+^ are attributed to the increased reactivity of DP^Tol^-PSMAt over DP^Ph^-PSMAt. Both *cis*/*trans*-[^99m^TcO_2_(DP^Ph^-PSMAt)_2_]^+^ and *cis*/*trans*-[^99m^TcO_2_(DP^Tol^-PSMAt)_2_]^+^ exhibited high metabolic stability, and *in vivo* SPECT imaging in healthy mice revealed that both new radiotracers cleared rapidly from circulation, via a renal pathway. These new diphosphine bioconjugates also furnished [^64^Cu(DP^X^-PSMAt)_2_]^+^ (X = Ph, Tol) complexes rapidly, in a high RCY (>95%), under mild conditions. In summary, the new DP platform is versatile: it enables straightforward functionalization of targeting peptides with a diphosphine chelator, and the resulting bioconjugates can be simply radiolabeled with both the SPECT and PET radionuclides, ^99m^Tc and ^64^Cu, in high RCYs. Furthermore, the DP platform is amenable to derivatization to either increase the chelator reactivity with metallic radioisotopes or, alternatively, modify the radiotracer hydrophilicity. Functionalized diphosphine chelators thus have the potential to provide access to new molecular radiotracers for receptor-targeted imaging.

## Abstract 2

Derivatives of 2,3-bis(diarylphosphino)maleic anhydride enable synthetic access to diphosphine−peptide bioconjugates for efficient radiolabeling and molecular imaging with the SPECT isotope, ^99m^Tc, or the PET isotope, ^64^Cu.

## Introduction

Single photon emission computed tomography (SPECT) and positron emission tomography (PET) with radiopharmaceuticals allow whole-body molecular imaging. One class of PET and SPECT radiopharmaceuticals incorporates a radioactive metal bound via a chelator attached to a peptide, which targets cell-surface receptors of diseased cells.^1^ The γ-emitting radionuclide technetium-99m (^99m^Tc, *t*_1/2_ = 6 h, 90% γ, 140 keV) and the positron-emitting radionuclide copper-64 (^64^Cu, *t*_1/2_ = 12.7 h, β^+^*E*_max_ = 656 keV, 19%) have both been used to radiolabel and subsequently image peptides for molecular SPECT/γ-scintigraphy and PET imaging, respectively. ^99m^Tc is largely produced by benchtop generators, enabling widespread access, while ^64^Cu can be produced by both cyclotrons and reactors. Both ^99m^Tc- and ^64^Cu-labeled receptor-targeted peptides have demonstrated clinical diagnostic value in the management of cancer.^2−4^

Radiopharmaceuticals based on ^99m^Tc are widely used, with approximately 30 million imaging procedures performed worldwide every year.^5^ The majority of these radiopharmaceuticals are used for imaging perfusion (as opposed to molecular) processes. These relatively simple ^99m^Tc complexes are prepared using kit-based radiosynthetic protocols in which the precursor ^99m^TcO_4_^–^ is simply eluted from a generator in a saline solution and added to commercially available “kit” vials that contain a reducing agent, a chelator, and other reagents.^6^ One of the challenges in developing ^99m^Tc or ^64^Cu radiometalated peptides for molecular imaging is designing chelators that allow simple, quantitative, and rapid radiolabeling in physiologically compatible solutions, using kits. Additionally, in these radiochemical reactions, the concentrations/amounts of both the chelator–peptide bioconjugate and radiometallic ion are very low, so favorable thermodynamics are required to drive formation of the desired complex. Finally, the resulting radiometalated complex needs to be sufficiently stable *in vivo* to resist transchelation of the radiometal to endogenous species in the biological milieu, such as proteins, minerals, and other biomolecules, which compete for metal binding.^1^ In radiolabeling reactions with ^99m^Tc, there are several accessible oxidation states; the selected chelator also needs to yield a well-defined complex that is inert in the presence of biological oxidants and reductants.^5^ One of the major challenges in developing chelators for ^64^Cu and other Cu radioisotopes is ensuring that the resulting complex is highly *kinetically* stable in biological media.^7^ Thus, the majority of these successful chelators are based on macrobicyclic species that complex Cu^2+^,^7^ but for the most part, these chelators have little utility in coordinating other radiometals.

Phosphine ligands form useful complexes with ^99m^Tc. The radiopharmaceutical “Myoview” is routinely used to image cardiac perfusion. In Myoview, two bidentate diphosphines coordinate to a Tc^V^ metal center, with two oxido ligands occupying axial positions.^8^ Myoview is prepared using a single step kit: ^99m^TcO_4_^–^ is added to a kit containing sodium gluconate, stannous chloride, sodium bicarbonate, and a diphosphine ligand, followed by incubation at room temperature for 15 min to produce Myoview in >90% yield, which is administered to patients without further processing.^9^ Other multidentate chelator systems designed specifically for the coordination of ^99m^Tc also have incorporated phosphine donors. These include P,S-bidentate and P_2_,N-tridentate ligands for coordinating the [TcN]^2+^ motif,^10,11^ P_2_,N- and P,S_2_-tridentate ligands for coordinating the [Tc(CO)_3_]^+^ motif,^12,13^ and P_2_,S_2_- and P_2_,N_2_-tetradentate ligands for coordinating the [TcO_2_]^+^ motif.^14,15^

We have recently described the use of 2,3-bis(diphenylphosphino)maleic anhydride (DP^Ph^) as a platform for simple preparation and ^99m^Tc radiolabeling of diphosphine–peptide conjugates.^16^ DP^Ph^ reacts with the primary amine of the pentapeptide, cyclic Arg-Gly-Asp-dPhe-Lys (RGD), to yield DP^Ph^-RGD. The conjugate DP^Ph^-RGD can be incorporated into “kits” containing DP^Ph^-RGD, reducing agent (stannous chloride), sodium tartrate, and sodium bicarbonate. The addition of ^99m^TcO_4_^–^ to these kits, followed by heating, produces a mixture of *cis*/*trans*-[^99m^TcO_2_(DP^Ph^-RGD)_2_]^+^ in high radiochemical yield (RCY >90%). [^99m^TcO_2_(DP^Ph^-RGD)_2_]^+^ shows high affinity and specificity for the target α_v_β_3_ integrin receptor, which is overexpressed in neovasculature, inflammation, and some cancers. We have also very recently shown that a diphosphine chelator derivatized with glucose units similarly coordinates the [^99m^TcO_2_]^+^ motif and that the resulting radiotracer is highly stable *in vivo* and exhibits favorable biodistribution properties, including fast renal clearance.^17^

**Scheme 1:**
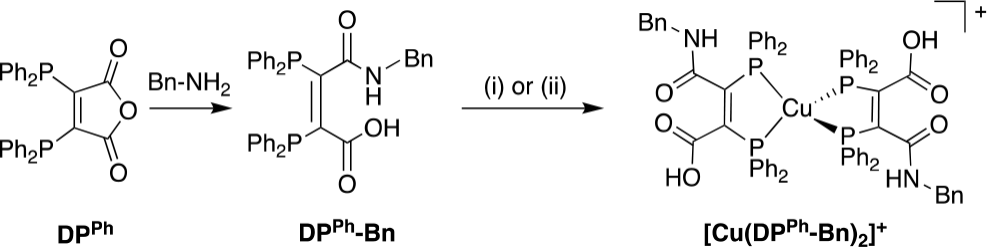
Preparation of [Cu(DP^Ph^-Bn)_2_]^+^ (i) CuCl; (ii) ^64^CuCl_2_.

Our work with DP^Ph^ builds upon others’ prior research, in which diphosphines^18,19^ including both DP^Ph^ and its benzylamine conjugate, DP^Ph^-Bn,^20,21^ were used to complex Cu^+^ to yield [Cu(DP^Ph^)_2_]^+^ and [Cu(DP^Ph^-Bn)_2_]^+^, respectively. Importantly, DP^Ph^-Bn could be radiolabeled with solutions of ^64^CuCl_2_ to give [^64^Cu(DP^Ph^-Bn)_2_]^+^ (Scheme 1). In these reactions, the excess of diphosphine acted as both a reducing agent, reducing ^64^Cu^2+^ to Cu^+^, and a bidentate chelator.

We postulated that bis(phosphino)maleic anhydride compounds could be versatile chemical platforms for radiolabeling with not only ^64^Cu but also ^99m^Tc. They could potentially (i) provide a flexible platform for appending receptor-targeted peptides/molecules to a diphosphine motif, (ii) enable simple, rapid, efficient, and stable radiolabeling of peptides with either the [^99m^TcO_2_]^+^ motif or ^64^Cu^+^, and (iii) allow improvement of the efficiency of radiolabeling protocols by varying phosphine substituents to increase the phosphine reactivity for complexation of [^99m^TcO_2_]^+^ or ^64^Cu^+^. To investigate this, we prepared and conjugated two bis(phosphino)maleic anhydride compounds to two different peptides: (a) “RGD” peptide, which targets the α_v_β_3_ integrin receptor overexpressed in neovasculature, inflammation, and many cancers; (b) “PSMAt”, which targets the prostate specific membrane antigen, overexpressed in prostate cancer. The new diphosphine–peptide conjugates were radiolabeled with both ^99m^Tc and ^64^Cu radionuclides. We also compared the electronic properties of these phosphine derivatives from the IR spectra of their [Mo(CO)_4_L] (L = bidentate diphosphine) complexes.

## Results

### Synthesis of DP^Ph^ and DP^Tol^

**Scheme 2:**
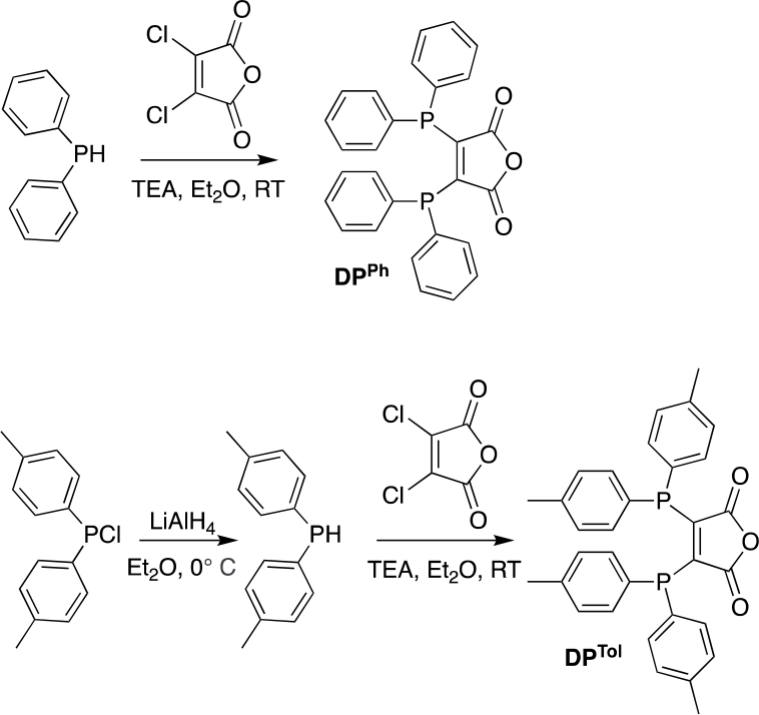
Preparation of DP^Ph^

The diphosphine compound DP^Ph^ has been used by us and others for diverse applications, including molecular imaging.^16,20−23^ It has been shown previously that DP^Ph^ can be prepared from diphenyl(trimethylsilyl)phosphine and dichloromaleic anhydride.^22,24^ Here, DP^Ph^ was instead prepared directly from diphenylphosphine and dichloromaleic anhydride in the presence of trimethylamine in diethyl ether (Scheme 2, top). Following isolation and removal of phosphine oxide side products, DP^Ph^ was obtained in 36% yield.

A second diphosphine derivative, 2,3-bis(di-*p*-tolylphosphino)maleic anhydride (DP^Tol^), has been prepared by a similar route (Scheme 2, bottom). Phosphine derivatives containing *p*-tolyl substituents in place of phenyl groups have demonstrated increased σ-donor capacity.^25,26^ We postulated that peptide derivatives of DP^Tol^ would provide increased RCYs in reactions with ^99m^Tc or ^64^Cu, relative to DP^Ph^ derivatives. DP^Tol^ was prepared in two steps from the commercial starting material bis(*p*-tolyl)chlorophosphine. First, bis(*p*-tolyl)phosphine was formed in high purity (>95%) by the reduction of bis(*p*-tolyl)chlorophosphine with lithium aluminum hydride. Bis(*p*-tolyl)phosphine was then reacted with dichloromaleic anhydride in the presence of triethylamine (TEA) in diethyl ether. Following isolation and removal of phosphine oxide side products, DP^Tol^ was obtained in 86% yield.

### Evaluating the Donor Properties of DP^Ph^, DP^Tol^, and Derivatives: IR Spectra of Mo Complexes

Complexes of the type *cis*-[Mo(CO)_4_L_2_]^27^ are widely used to assess the binding properties of a variety of ligands: IR stretching frequencies of CO ligands are a useful indicator of σ-donor/π-acceptor characteristics of ligand “L”. The diphosphines used in this study are primarily σ-donor ligands, and the stronger the σ donor, the greater the π-back-bonding from Mo to CO, and the lower the CO stretching frequency will be.

**Scheme 3:**
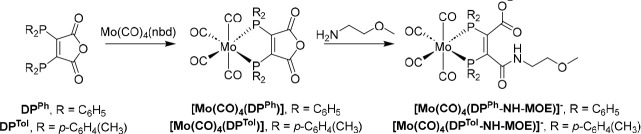
Mo Complexes of DP^Ph^ and DP^Tol^ Derivatives

[Mo(CO)_4_(nbd)] (nbd = norbornadiene) was reacted with either DP^Ph^ or DP^Tol^ at ambient temperature in dichloromethane (Scheme 3). The reactions were monitored by ^31^P{^1^H} NMR spectroscopy; [Mo(CO)_4_(DP^Tol^)] was formed in >95% yield within 1 day, while [Mo(CO)_4_(DP^Ph^)] required 3 days of reaction to achieve comparable yields. To generate a model for the DP–peptide conjugate species, [Mo(CO)_4_(DP^Ph^)] and [Mo(CO)_4_(DP^Tol^)] were each reacted with an excess of (2-methoxyethyl)amine in dichloromethane, yielding [Mo(CO)_4_(DP^Ph^-NH-MOE]^−^ and [Mo(CO)_4_(DP^Tol^-NH-MOE)]^−^, respectively (MOE = methoxyethane; Scheme 3). The ^31^P{^1^H} NMR spectra of the reaction mixtures revealed that these species were formed quantitatively and rapidly. These complexes were isolated as (2-methoxyethyl)ammonium salts.

IR and NMR spectra were acquired for all isolated Mo complexes (see Table 1 and the Supporting Information, SI). There was a decrease in ν_CO_ in the order [Mo(CO)_4_(nbd)] > [Mo(CO)_4_(DP^Ph^)] > [Mo(CO)_4_(DP^Tol^)] > [Mo(CO)_4_(DP^Ph^-NH-MOE)]^−^ > [Mo(CO)_4_(DP^Tol^-NH-MOE)]^−^. Importantly, the ν_CO_ values are lower for [Mo(CO)_4_(DP^Tol^)] relative to [Mo(CO)_4_(DP^Ph^)], and the ν_CO_ values are lower for [Mo(CO)_4_(DP^Tol^-NH-MOE)]^−^ relative to [Mo(CO)_4_(DP^Ph^-NH-MOE)]^−^. These observed reductions in ν_CO_ for DP^Tol^ complexes relative to DP^Ph^ complexes are consistent with DP^Tol^ derivatives possessing increased σ-donor capacities compared to DP^Ph^ derivatives. Additionally, ν_CO_ values of complexes [Mo(CO)_4_(DP^Ph^-NH-MOE)]^−^ and [Mo(CO)_4_(DP^Tol^-NH-MOE)]^−^ were lower than those of [Mo(CO)_4_(DP^Ph^)] and [Mo(CO)_4_(DP^Tol^)], indicating (as expected) that DP-NHR ligands are significantly better σ-donor ligands than the bis(phosphino)maleic anhydride precursors.

**Table 1:** Spectroscopic Data for Mo Complexes and DP^Ph^ and DP^Tol^ Ligands

compound	ν_CO_ (cm^–1^)	^31^P{^1^H} NMR (ppm)
DP^Ph^		–20.5 (s)^a^
DP^Tol^		–23.1 (s)^a^
[Mo(CO)_4_(nbd)]	2041 (s), 1980 (sh), 1951 (s), 1888 (s)	
[Mo(CO)_4_(DP^Ph^)]	2031 (s), ∼1938 (sh), 1920 (s), 1775 (s)	49.9 (s)^b^
[Mo(CO)_4_(DP^Tol^)]	2029 (s), ∼1935 (sh), 1916 (s), 1774 (s)	48.4 (s)^b^
[MOE-NH_3_][Mo(CO)_4_(DP^Ph^-NH-MOE]	2024 (s), 1931 (s), 1902 (s)	72.5 (d, *J* = 3.3 Hz), 70.2 (d, *J* = 3.3 Hz)^c^
[MOE-NH_3_][Mo(CO)_4_(DP^Tol^-NH-MOE)]	2022 (s), 1928 (s), 1900 (s)	70.8 (d, *J* = 2.4 Hz), 68.5 (d, *J* = 2.4 Hz)^c^

a 162 MHz, CDCl_3_.

b 122 MHz, CD_2_Cl_2_.

c 162 MHz, CD_2_Cl_2_.

### DP^Ph^ and DP^Tol^ Peptide Conjugates and Their Re and Tc Complexes

We next aimed to prepare diphosphine peptide conjugates by reacting DP^Ph^ and DP^Tol^ with peptides containing single primary amine groups. We have recently shown that the cyclic pentapeptide, c(RGDfK) (RGD), reacts with DP^Ph^ under basic conditions to give DP^Ph^-RGD.^16^ The reaction of DP^Tol^ under the same conditions yielded the analogous conjugate DP^Tol^-RGD in 57% yield.

**Scheme 4:**
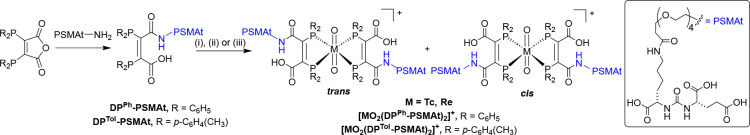
Preparation and Complexation of DP-PSMAt Conjugates (i) [ReO_2_I(PPh_3_)_2_] in DMF; (ii) [N^*t*^Bu_4_][^99g^TcOCl_4_] in DMF; (iii) ^99m^TcO_4_^–^, SnCl_2_, sodium tartrate, in water (pH 8).

The PSMAt peptide, which targets the prostate specific membrane antigen, has been clinically used to target imaging and therapeutic radioisotopes to prostate cancer. Here, a linker consisting of a tetraethylene glycol unit (to increase the water solubility of the resulting conjugates) with a single pendant primary amine was appended to the dipeptide PSMAt pharmacophore. The reaction of this PSMAt peptide with either DP^Ph^ or DP^Tol^ furnished DP^Ph^-PSMAt or DP^Tol^-PSMAt (Scheme 4), respectively, which were isolated using preparative reverse-phase high-performance liquid chromatography (HPLC) and characterized by ^1^H and ^31^P{^1^H} NMR spectroscopy and high-resolution electrospray ionization mass spectrometry (HR-ESI-MS; *vide infra*, SI). Both conjugates were obtained in over 60% yield and were freely soluble in water.

In the solid state, all three of these new conjugates—DP^Tol^-RGD, DP^Ph^-PSMAt, and DP^Tol^-PSMAt—were stable to oxidation of tertiary phosphine centers in air, although they slowly oxidized in solution to phosphine oxide derivatives under normal atmospheric conditions. For experimental purposes, the conjugates could be handled in air as dry material, in basic organic solutions, or in aqueous solutions at near-neutral pH. However, in acidic solutions, DP–peptide conjugates reformed the starting peptide and bis(phosphino)maleic anhydride.

DP^Ph^-PSMAt and DP^Tol^-PSMAt were each reacted with [ReO_2_I(PPh_3_)_2_] (Scheme 4), and the resulting [ReO_2_(DP^Ph^-PSMAt)_2_]^+^ and [ReO_2_(DP^Tol^-PSMAt)_2_]^+^ complexes were isolated and analyzed by HR-ESI-MS, ^1^H and ^31^P{^1^H} NMR spectroscopy, and reverse-phase HPLC. In the HR-ESI-MS spectra, signals consistent with [M + H]^2+^ ions were detected (*m*/*z* 1142.3402 where M = [ReO_2_(DP^Ph^-PSMAt)_2_]^+^ and *m*/*z* 1198.4039 where M = [ReO_2_(DP^Tol^-PSMAt)_2_]^+^).

The putative cis and trans isomers that are possible for each rhenium complex of the PSMAt conjugates possessed closely similar chromatographic behavior, and we were unable to isolate one isomer from another (as was previously achieved for cis and trans isomers of the homologous RGD-based complex,^16^ [ReO_2_(DP^Ph^-RGD)_2_]^+^).

**Figure 1:**
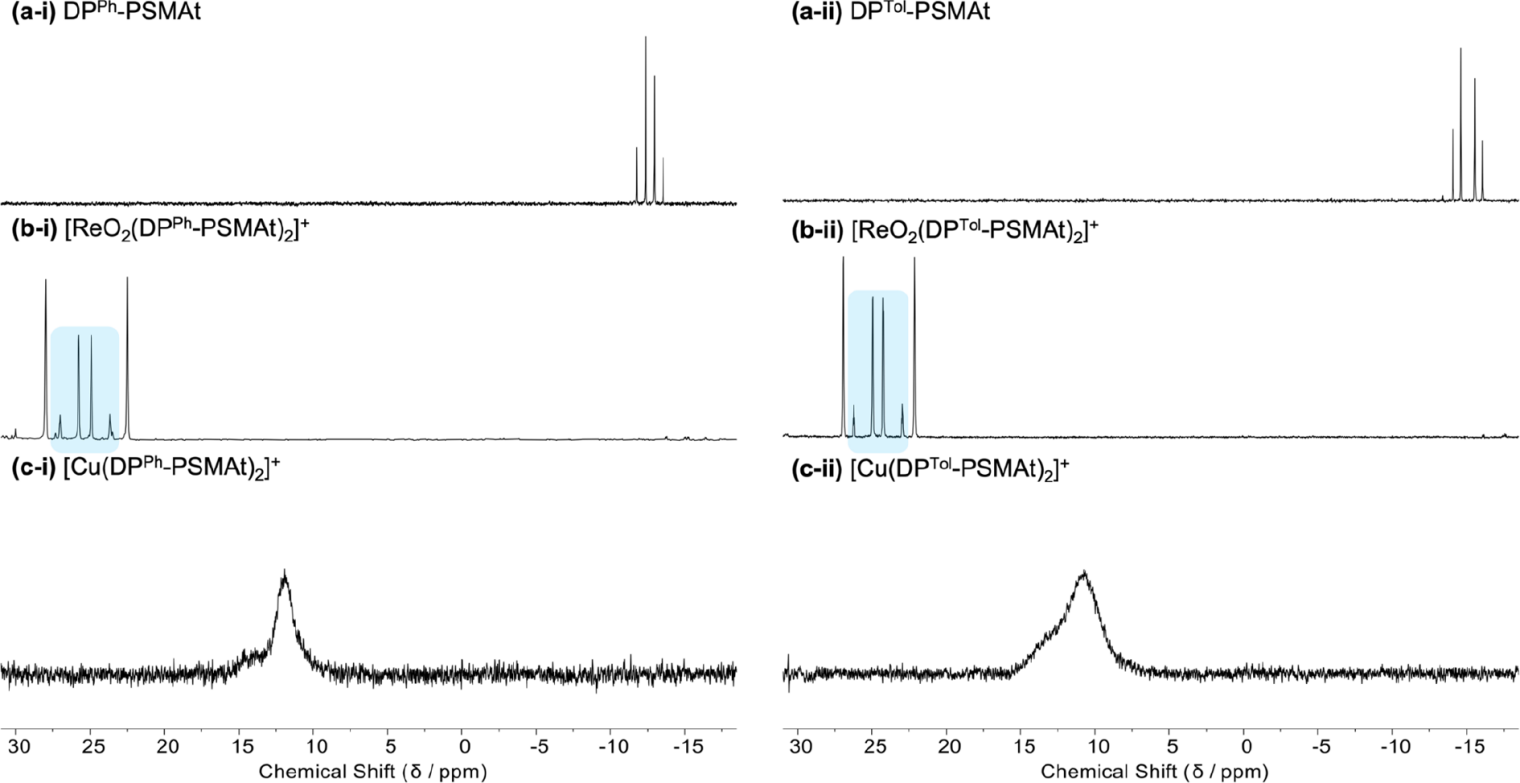
^31^P{^1^H} NMR spectra of (a-i) DP^Ph^-PSMAt, (a-ii) DP^Tol^-PSMAt, (b-i) [^nat^ReO_2_(DP^Ph^-PSMAt)_2_]^+^, (b-ii) [^nat^ReO_2_(DP^Tol^-PSMAt)_2_]^+^, (c-i) [^nat^Cu(DP^Ph^-PSMAt)_2_]^+^, and (c-ii) [^nat^Cu(DP^Tol^-PSMAt)_2_]^+^. Signals corresponding to *cis*-[^nat^ReO_2_(DP^Ph^-PSMAt)_2_]^+^ and *cis*-[^nat^ReO_2_(DP^Tol^-PSMAt)_2_]^+^ are highlighted in blue.

In the ^31^P{^1^H} NMR spectra of each of the free ligands, DP^Ph^-PSMA and DP^Tol^-PSMA, the two inequivalent P atoms produce an AB pattern (Figure 1a). Geometric cis and trans isomers of [ReO_2_(DP^Ph^-PSMAt)_2_]^+^ and [ReO_2_(DP^Tol^-PSMAt)_2_]^+^ are expected to exhibit ^31^P{^1^H} NMR splitting patterns of AA′BB′ spin systems. Acquired ^31^P{^1^H} NMR spectra exhibited two distinct pairs of signals typical of the presence of both cis and trans isomers (Figure 1b). In each spectrum, the pair of signals with a pseudo-AB coupling pattern and a large ^2^*J*(P_A_P_B_) (∼360 Hz) was assigned to the cis isomer (consistent with a large ^2^*J*(P_A_P_B_) expected for trans-inequivalent P atoms); the remaining pair of signals was assigned to the trans isomer. To support these assignments, ^31^P{^1^H} NMR spectra were simulated as AA′BB′ spin systems (Figures S42 and S43). The good agreement between the experimental and simulated spectra supports the assignment of the isomers and is consistent with our prior observations of similar systems.^16,17^ In the ^1^H NMR spectra, aromatic phenyl or tolyl signals shift upon Re^V^ binding (SI, section 3).

**Figure 2:**
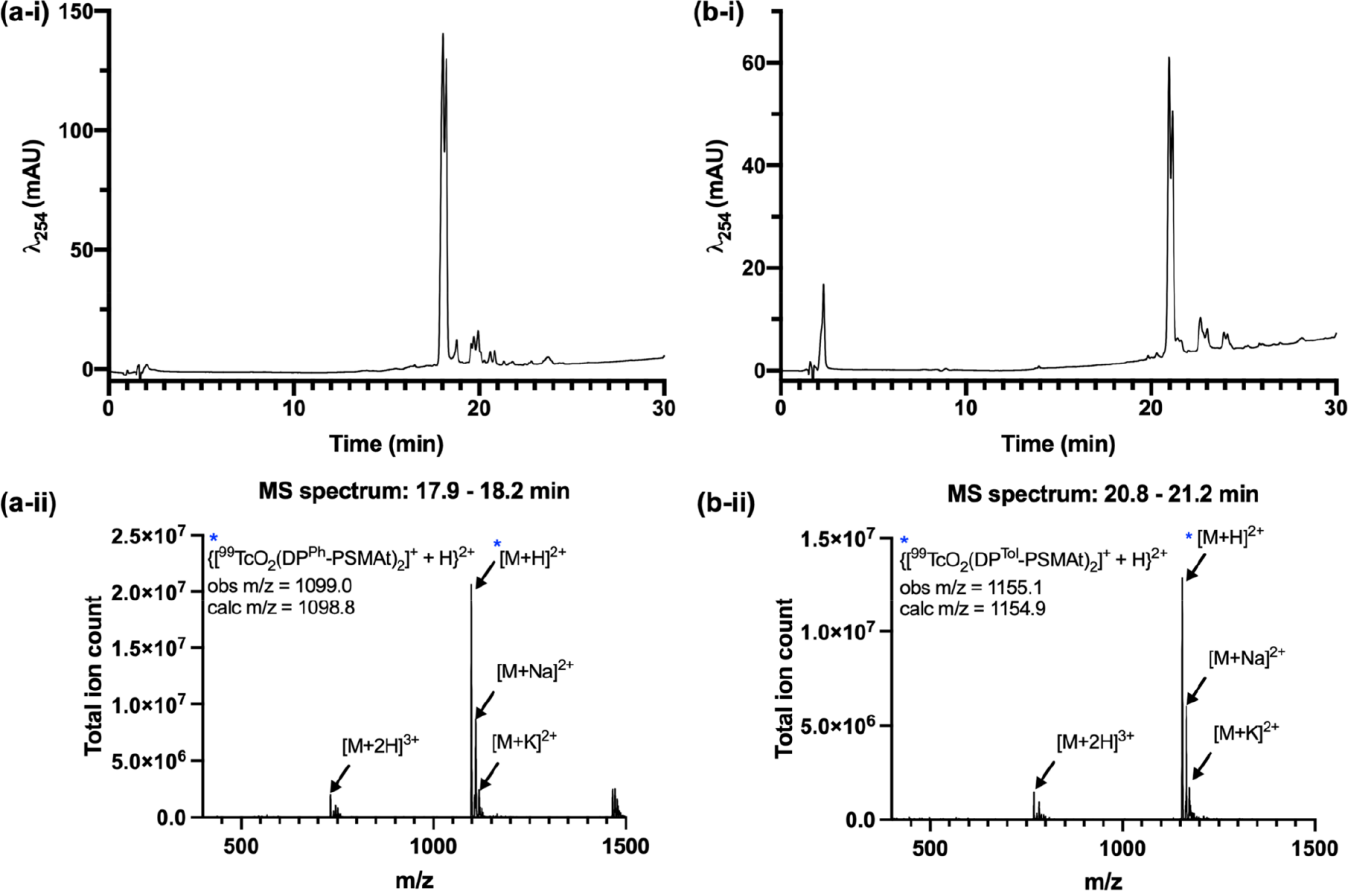
DP-PSMAt derivatives reacted with [N^*t*^Bu_4_][^99g^TcOCl_4_] to yield [^99g^TcO_2_(DP-PSMAt)_2_]^+^), which consists of both cis and trans isomers. (a-i) UV chromatogram of [^99g^TcO_2_(DP^Ph^-PSMAt)_2_]^+^); (a-ii) MS chromatogram of [^99g^TcO_2_(DP^Ph^-PSMAt)_2_]^+^; (b-i) UV chromatogram of [^99g^TcO_2_(DP^Tol^-PSMAt)_2_]^+^; (b-ii) MS chromatogram of [^99g^TcO_2_(DP^Tol^-PSMAt)_2_]^+^. For HPLC method 8, see the SI.

DP^Ph^-PSMAt and DP^Tol^-PSMAt were each reacted with [N^*t*^Bu_4_][^99g^TcOCl_4_] (^99g^Tc, *t*_1/2_ = 211000 years), and the resulting [TcO_2_(DP^Ph/Tol^-PSMAt)_2_]^+^ complexes were analyzed by reverse-phase C_18_ HPLC–LR-MS. For each compound, the UV chromatogram (λ = 254 nm) of the LC–MS showed two strongly absorbing signals that corresponded to species with a formula of [TcO_2_(DP^Ph/Tol^-PSMAt)_2_]^+^ (Figure 2). These isomeric pairs eluted within 0.25 min of each other and were attributed to the presence of cis and trans isomers for each complex. This chromatographic behavior is similar to that of *cis*-[MO_2_(DP^Ph^-RGD)_2_]^+^ and *trans*-[MO_2_(DP^Ph^-RGD)_2_]^+^ (M = ^99g^Tc, Re).

Lastly, the putative *cis*-[^99g^TcO_2_(DP^Ph/Tol^-PSMAt)_2_]^+^ and *trans*-[TcO_2_(^99g^DP^Ph/Tol^-PSMAt)_2_]^+^ species exhibited near-identical HPLC retention times to analogous Re complexes, indicative of the structural homology between Tc and Re species (Figure S57).

### ^99m^Tc Radiolabeling

To assess radiolabeling with ^99m^Tc, lyophilized, prefabricated kits were prepared, containing a diphosphine–peptide conjugate, a reducing agent (stannous chloride), a “weak” chelator to stabilize any Tc intermediates (sodium tartrate), and a sodium bicarbonate buffer. Generator-produced ^99m^TcO_4_^–^ (200 MBq) in a saline solution (300 μL) was then added to these kits, and the mixtures were heated at 100 °C for 5 min, prior to analysis by radio-iTLC and radio-HPLC. These reactions were also undertaken at ambient temperature for comparison. RCYs were determined by iTLC.

At both ambient temperature (20–25 °C) and 100 °C, both [^99m^TcO_2_(DP^Ph^-PSMAt)_2_]^+^ and [^99m^TcO_2_(DP^Tol^-PSMAt)_2_]^+^ could be prepared from kits in >75% RCY in 5 min (Table 2). For both [^99m^TcO_2_(DP^Ph^-PSMAt)_2_]^+^ and [^99m^TcO_2_(DP^Tol^-PSMAt)_2_]^+^, RCYs were higher at 100 °C compared to RCYs at ambient temperature. Under both conditions, the concomitant formation of ^99m^Tc-labeled colloidal material was the main factor that decreased RCY. As hypothesized, RCYs for [^99m^TcO_2_(DP^Tol^-PSMAt)_2_]^+^ were significantly higher than RCYs for [^99m^TcO_2_(DP^Ph^-PSMAt)_2_]^+^ at both ambient temperature and 100 °C. At 100 °C, the RCY for [^99m^TcO_2_(DP^Tol^-PSMAt)_2_]^+^ (88.0 ± 0.6%) was higher than that for [^99m^TcO_2_(DP^Ph^-PSMAt)_2_]^+^ (81.2 ± 1.8%, mean difference = 6.8%, and *p* = 0.007); at ambient temperature, the RCY for [^99m^TcO_2_(DP^Tol^-PSMAt)_2_]^+^ (83.5 ± 1.5%) was higher than that for [^99m^TcO_2_(DP^Ph^-PSMAt)_2_]^+^ (75.3 ± 3.0%, mean difference = 8.2%, and *p* = 0.026).

**Table 2:** RCYs (%) of [^99m^TcO_2_(DP^Ph^-PSMAt)_2_]^+^ and [^99m^TcO_2_(DP^Tol^-PSMAt)_2_]^+^ (Determined Using iTLC)^a^

	22 °C	100 °C
[^99m^TcO_2_(DP^Ph^-PSMAt)_2_]^+^	75.3 ± 3.0	81.2 ± 1.8
[^99m^TcO_2_(DP^Tol^-PSMAt)_2_]^+^	83.5 ± 1.5	88.0 ± 0.6

a Radiochemical reactions were performed in triplicate (±standard deviation).

**Figure 3:**
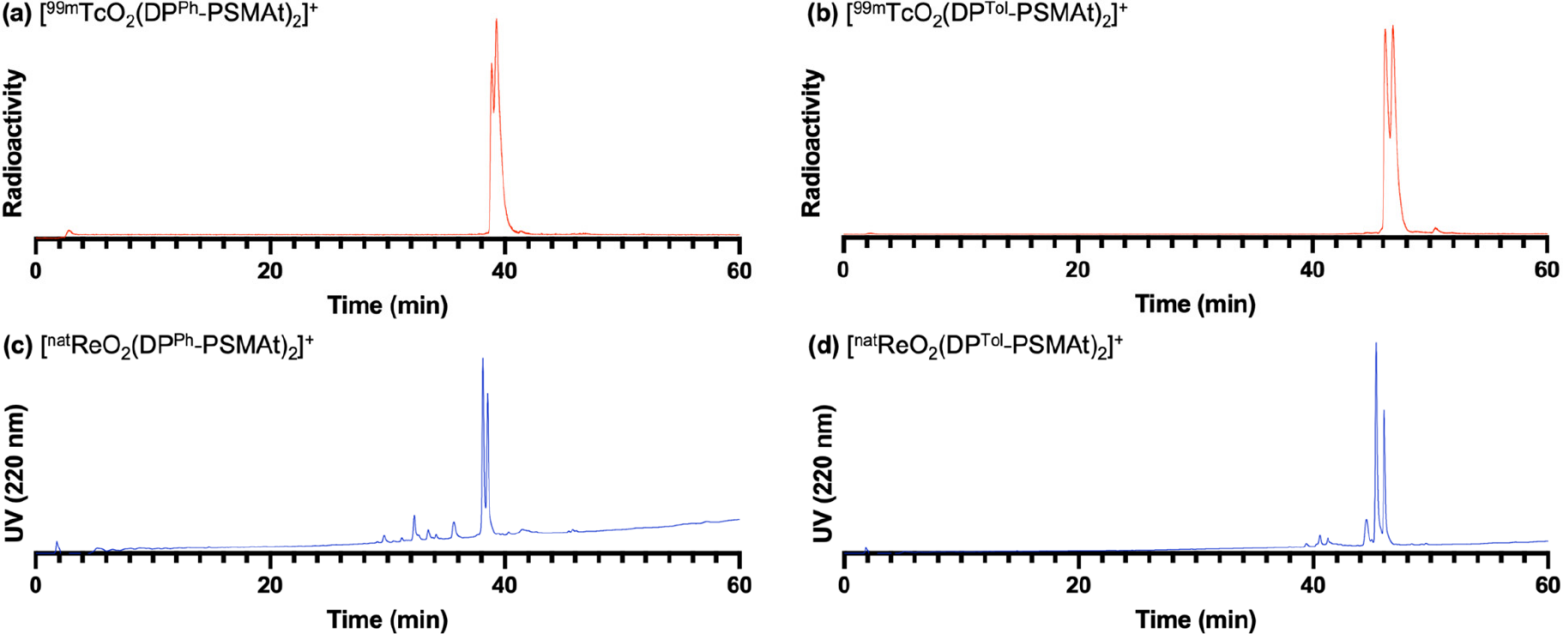
Putative cis and trans isomers of (a) [^99m^TcO_2_(DP^Ph^-PSMAt)_2_]^+^ and (b) [^99m^TcO_2_(DP^Tol^-PSMAt)_2_]^+^, separated on a shallow analytical C_18_ HPLC gradient. The radioactive signals were coincident with the UV signals of characterized (c) [^nat^ReO_2_(DP^Ph^-PSMAt)_2_]^+^ and (d) [^nat^ReO_2_(DP^Tol^-PSMAt)_2_]^+^. For HPLC method 10, see the SI.

The reaction products were also analyzed by analytical reverse-phase C_18_ HPLC. When these kit-based reactions were undertaken at 100 °C, aside from small amounts of unreacted ^99m^TcO_4_^–^ (eluting at 2.3 min), the only radiolabeled products observed in the radio chromatograms corresponded to putative cis and trans isomers of either [^99m^TcO_2_(DP^Ph^-PSMAt)_2_]^+^ or [^99m^TcO_2_(DP^Tol^-PSMAt)_2_]^+^ (Figure 3). Importantly, these radioactive signals were near-coincident with the UV signals of characterized [ReO_2_(DP^Ph^-PSMAt)_2_]^+^ and [ReO_2_(DP^Tol^-PSMAt)_2_]^+^ complexes. When these kit-based reactions were performed at ambient temperature, low amounts of additional ^99m^Tc-labeled products were observed in the radiochromatograms (4–5%), eluting at earlier retention times (Figure S55).

### Stability and Biodistribution of [^99m^TcO_2_(DP^Ph^-PSMAt)_2_]^+^ and [^99m^TcO_2_(DP^Tol^-PSMAt)_2_]^+^ in Healthy Mice

To evaluate the biological behavior of each radiotracer, kit-based radiolabeling solutions were purified using size-exclusion HPLC, enabling [^99m^TcO_2_(DP^Ph^-PSMAt)_2_]^+^ and [^99m^TcO_2_(DP^Tol^-PSMAt)_2_]^+^ to be isolated from unreacted ^99m^TcO_4_^–^, ^99m^Tc colloids, and unreacted DP-PSMAt conjugate.

[^99m^TcO_2_(DP^Ph^-PSMAt)_2_]^+^ and [^99m^TcO_2_(DP^Tol^-PSMAt)_2_]^+^ were each added to human serum and incubated at 37 °C for 24 h. Analytical reverse-phase radio-HPLC analysis of serum samples indicated that both [^99m^TcO_2_(DP^Ph^-PSMAt)_2_]^+^ and [^99m^TcO_2_(DP^Tol^-PSMAt)_2_]^+^ exhibited high stability, with over 90% of each radiotracer remaining intact over 24 h. With the exception of a species with a retention time of 2.5 min, which was attributed to dissociated, oxidized ^99m^TcO_4_^–^, no other degradation products were observed in radio-HPLC chromatograms (Table 3).

**Table 3:** Amount of Dissociated ^99m^Tc (%) after Incubation of [^99m^TcO_2_(DP^Ph^-PSMAt)_2_]^+^ and [^99m^TcO_2_(DP^Tol^-PSMAt)_2_]^+^ with Serum

incubation time (h)	[^99m^TcO_2_(DP^Ph^-PSMAt)_2_]^+^	[^99m^TcO_2_(DP^Tol^-PSMAt)_2_]^+^
1	0	0.1
4	0.7	1.6
24	4.2	6.5

The log *D*_OCT/PBS_ of [^99m^TcO_2_(DP^Ph^-PSMAt)_2_]^+^ was −2.45 and the log *D*_OCT/PBS_ of [^99m^TcO_2_(DP^Tol^-PSMAt)_2_]^+^ was −2.08, indicating that both are relatively hydrophilic, despite the multiple phenyl or tolyl groups present.

**Figure 4:**
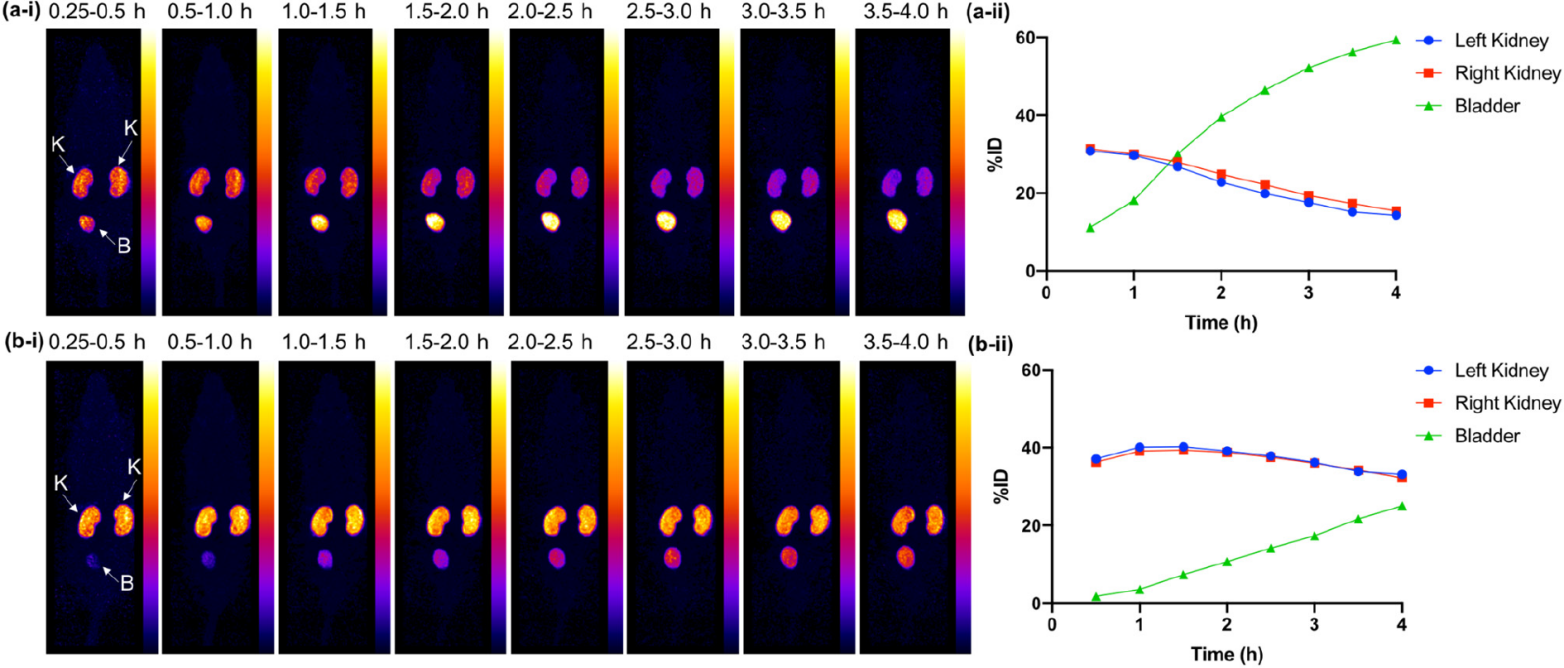
Maximum intensity projections of healthy male SCID Beige mice injected with (a-i) [^99m^TcO_2_(DP^Ph^-PSMAt)_2_]^+^ and (b-i) [^99m^TcO_2_(DP^Tol^-PSMAt)_2_]^+^ from 15 min to 4 h postinjection. Regions of interest were selected on VivoQuant (inviCRO, LLC, Boston, MA), and percentages of injected dose per milliliter (% ID/mL) were calculated for each of (a-ii) [^99m^TcO_2_(DP^Ph^-PSMAt)_2_]^+^ (*n* = 1) and (b-ii) [^99m^TcO_2_(DP^Tol^-PSMAt)_2_]^+^ (*n* = 1). K = kidneys; B = bladder.

**Figure 5:**
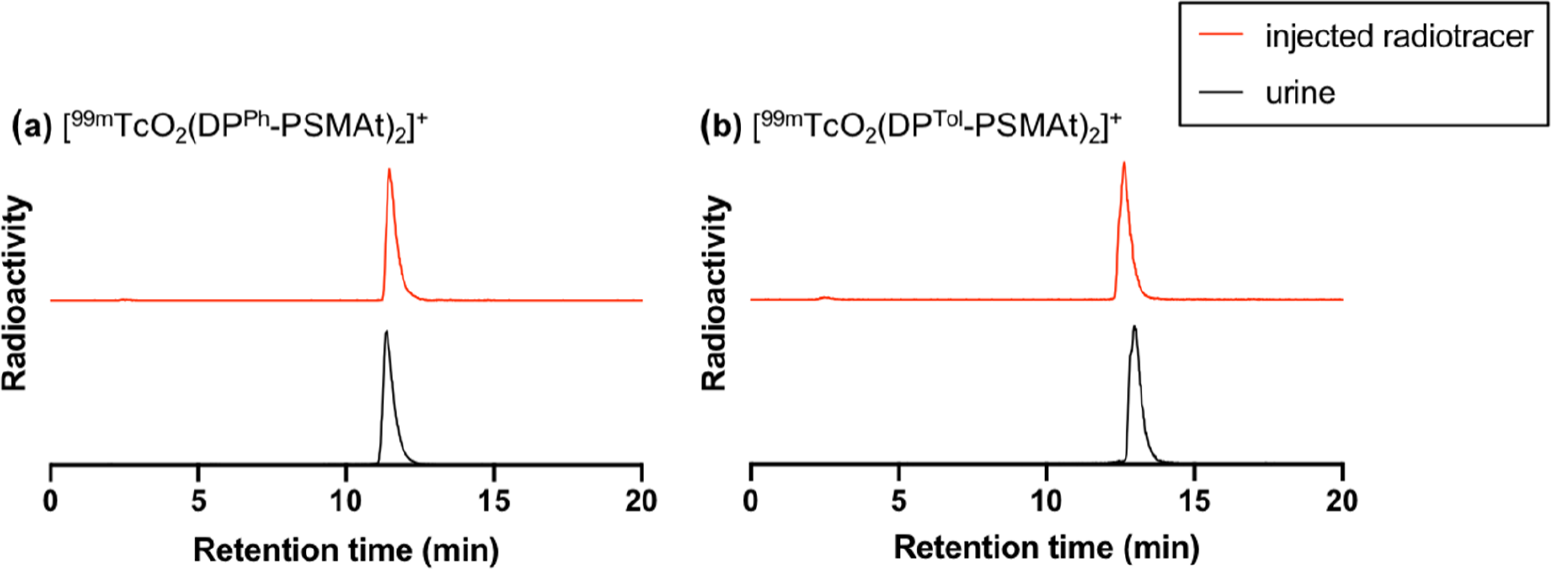
Radio-HPLC analysis of urine from healthy male SCID Beige mice intravenously administered with either (a) [^99m^TcO_2_(DP^Ph^-PSMAt)_2_]^+^ or (b) [^99m^TcO_2_(DP^Tol^-PSMAt)_2_]^+^. Radio-HPLC shows that both radiotracers are highly metabolically stable and are excreted intact. For HPLC method 2, see the SI.

In preliminary *in vivo* SPECT imaging studies assessing the biodistribution of these radiotracers, healthy male SCID Beige mice were administered either [^99m^TcO_2_(DP^Ph^-PSMAt)_2_]^+^ or [^99m^TcO_2_(DP^Tol^-PSMAt)_2_]^+^. SPECT imaging (Figure 4), undertaken 15 min to 4 h postinjection of each radiotracer, indicated that (i) both [^99m^TcO_2_(DP^Ph^-PSMAt)_2_]^+^ and [^99m^TcO_2_(DP^Tol^-PSMAt)_2_]^+^ cleared from circulation via a renal pathway with increasing amounts of ^99m^Tc radioactivity measured in urine over 4 h and (ii) [^99m^TcO_2_(DP^Ph^-PSMAt)_2_]^+^ cleared from the kidneys to the bladder faster than [^99m^TcO_2_(DP^Tol^-PSMAt)_2_]^+^. Urine was also collected (4 h postinjection) and analyzed by analytical reverse-phase radio-HPLC (Figure 5). Both [^99m^TcO_2_(DP^Ph^-PSMAt)_2_]^+^ and [^99m^TcO_2_(DP^Tol^-PSMAt)_2_]^+^ were excreted intact, with no other ^99m^Tc species detectable, indicating that the two radiotracers possess very high metabolic stability.

### Cu Complexes of DP-PSMAt Conjugates

**Scheme 5:**
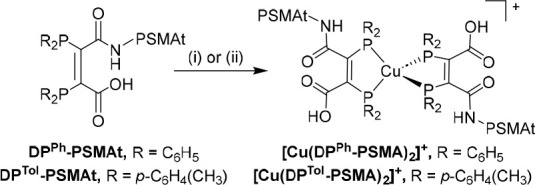
Reaction of DP-PSMAt Conjugates with Cu^+^ (i) [Cu(MeCN)_4_][PF_6_] in mixtures of water and acetonitrile; (ii) solutions of ^64^Cu^2+^ with a large excess of DP-PSMAt conjugate in an aqueous solution.

Prior studies^20,21^ have shown that DP^Ph^-Bn reacts with solutions of Cu^+^ to give [Cu(DP^Ph^-Bn)_2_]^+^. Here, each DP-PSMAt conjugate (2 equiv) was reacted with [Cu(MeCN)_4_][PF_6_] in mixtures of water and acetonitrile (ambient temperature, 30–60 min), with each reaction analyzed by analytical reverse-phase C_18_ HPLC. Each chromatographic trace (λ = 254 nm) showed a single, strongly-absorbing species. MS analysis of the reaction solutions was consistent with the formation of [Cu(DP^Ph^-PSMAt)_2_]^+^ (for [M + H]^2+^, *m*/*z* 1065.3402 (obsd) and 1065.3385 (calcd)) and [Cu(DP^Tol^-PSMAt)_2_]^+^ (for [M + H]^2+^, *m*/*z* 1121.3953 (obsd) and 1121.4012 (calcd); Scheme 5).

The ^31^P{^1^H} NMR spectrum of [Cu(DP^Ph^-PSMAt)_2_]^+^ exhibits a single broad, asymmetric peak at 11.94 ppm; for [Cu(DP^Tol^-PSMAt)_2_]^+^, a similar peak is observed at 10.81 ppm (Figure 1c). The broadness of these resonances obscures distinction of the two chemically inequivalent P atoms in each of these complexes. In the ^1^H NMR spectra of [Cu(DP^Ph^-PSMAt)_2_]^+^ and [Cu(DP^Tol^-PSMAt)_2_]^+^, the resonances of the diphenyl/ditolylphosphine protons and PEG linker protons that are in the closest vicinity to the Cu^+^ center are broad (Figure S8). In contrast, ^1^H signals for the PSMAt dipeptide motif are significantly sharper. These ^1^H and ^31^P{^1^H} NMR spectral line shapes are typical of tetrakis(phosphine) complexes of Cu^+^, in which fast quadrupolar relaxation times are associated with ^63^Cu and ^65^Cu, which both have nuclear spins of *I* = ^3^/_2_. This becomes particularly apparent in asymmetric complexes: similar spectral features have been described for Cu^+^ tetrahedral complexes, including those in which two unsymmetrical bidentate diphosphine ligands coordinate Cu^+^.^28,29^

### ^64^Cu Radiolabeling and Serum Stability

**Figure 6:**
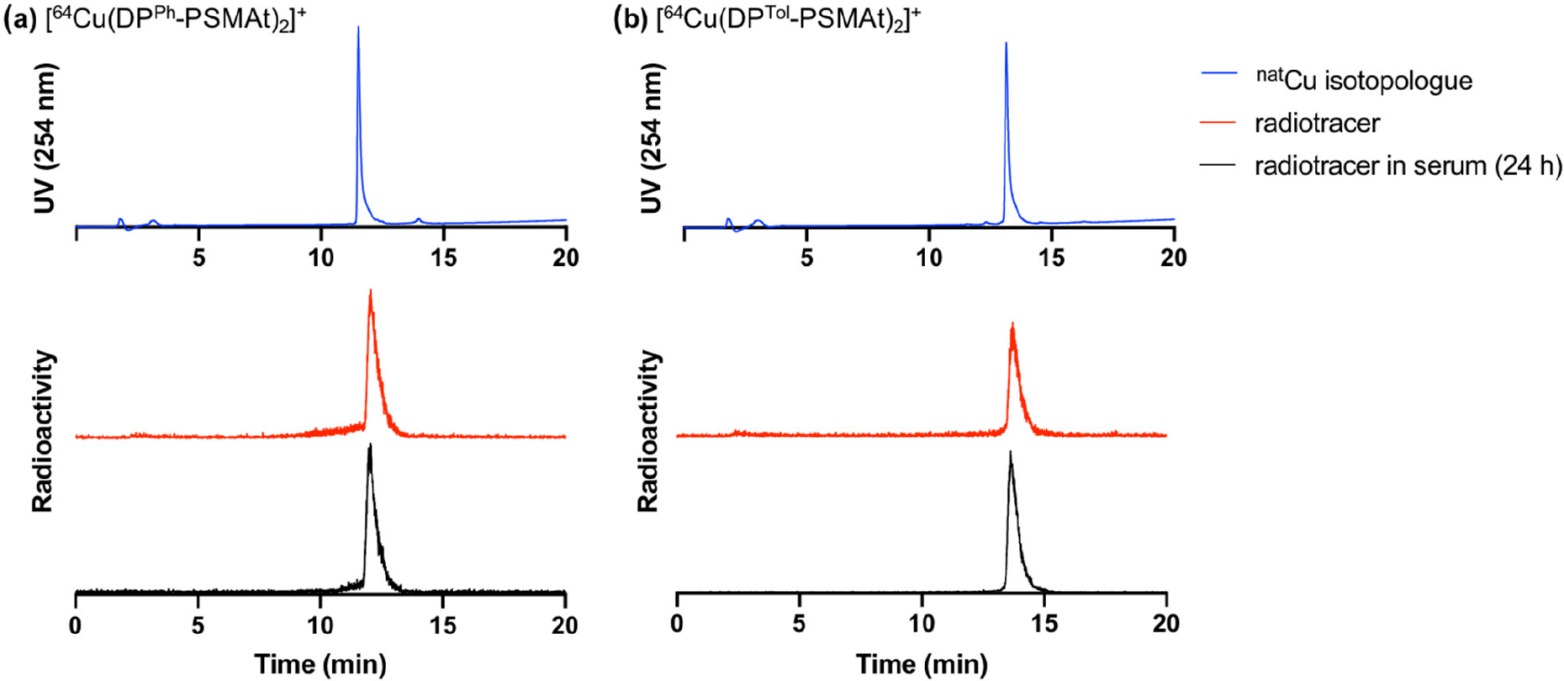
HPLC chromatograms of (a) [Cu(DP^Ph^-PSMAt)_2_]^+^ and (b) [Cu(DP^Tol^-PSMAt)_2_]^+^. DP-PSMAt derivatives were reacted with solutions of either [^nat^Cu(MeCN)_4_][PF_6_] (blue traces) or ^64^Cu^2+^ (red traces), with UV signals for [^nat^Cu(DP-PSMAt)_2_]^+^ derivatives coincident with radioactive signals for [^64^Cu(DP-PSMAt)_2_]^+^ (with slight differences in the retention times a result of the configuration of the UV and scintillation detectors in series). Analytical radio-HPLC analysis revealed that both radiotracers were stable in serum over 24 h (black traces). For HPLC method 2, see the SI.

DP^Ph^-PSMAt and DP^Tol^-PSMAt (50 μg) were each reacted with solutions of ^64^Cu^2+^ (5–10 MBq, in an aqueous solution of 0.1 M ammonium acetate, pH 7) at ambient temperature for 20 min. Analysis by analytical reverse-phase radio-HPLC showed that each reaction yielded only a single radiolabeled product, which was formed in >95% RCY (retention times of 12.0 and 13.7 min for DP^Ph^-PSMAt and DP^Tol^-PSMAt, respectively; Figure 6). Importantly, radioactive signals for these products were coincident with UV signals for characterized nonradioactive [^nat^Cu(DP^Ph^-PSMAt)_2_]^+^ or [^nat^Cu(DP^Tol^-PSMAt)_2_]^+^ isotopologues. We postulate that when present in a large excess, DP-PSMAt conjugates are capable of reducing ^64^Cu^2+^ to ^64^Cu^+^, enabling the formation of [^64^Cu(DP^Ph^-PSMAt)_2_]^+^ or [^64^Cu(DP^Tol^-PSMAt)_2_]^+^ (Scheme 5). This is similar to the radiochemical preparation of [^64^Cu(DP^Ph^-Bn)_2_]^+^ from solutions containing DP^Ph^-Bn and ^64^Cu^2+^.^20^

log *D*_OCT/PBS_ of [^64^Cu(DP^Ph^-PSMAt)_2_]^+^ measured −3.30 and log *D*_OCT/PBS_ of [^64^Cu(DP^Tol^-PSMAt)_2_]^+^ measured −3.01, suggesting that both are relatively hydrophilic.

To assess the stability of [^64^Cu(DP^Ph^-PSMAt)_2_]^+^ or [^64^Cu(DP^Tol^-PSMAt)_2_]^+^ in the presence of serum proteins, each species was added to human serum and incubated at 37 °C. Radiochromatograms of the serum samples showed that [^64^Cu(DP^Ph^-PSMAt)_2_]^+^ and [^64^Cu(DP^Tol^-PSMAt)_2_]^+^ were still present, even after 24 h of incubation in serum, with no other degradation products detectable (Figure 6).

## Discussion and Concluding Remarks

We have shown that the two bis(phosphino)maleic anhydride compounds, DP^Ph^ and DP^Tol^, are versatile platforms for the preparation of receptor-targeted radiotracers. Both compounds react readily with primary amine groups of either RGD peptide or PSMAt peptide, and we envisage that other targeted biomolecules could similarly be derivatized with a diphosphine.

Furthermore, these diphosphine–peptide conjugates can be very easily radiolabeled with either the SPECT isotope, ^99m^Tc, or the PET isotope, ^64^Cu. The new ^99m^Tc radiotracers, [^99m^TcO_2_(DP^Ph^-PSMAt)_2_]^+^ and [^99m^TcO_2_(DP^Tol^-PSMAt)_2_]^+^, have been prepared in high RCYs (>75%) in 5 min, at either ambient temperature or 100 °C, by the simple addition of a solution of ^99m^TcO_4_^–^ to single kits containing all necessary reagents. It is likely that varying the amounts of different kit reagents will lead to even higher RCYs, and we are currently optimizing the formulation of these kits to this end. The new ^64^Cu radiotracers, [^64^Cu(DP^Ph^-PSMAt)_2_]^+^ and [^64^Cu(DP^Tol^-PSMAt)_2_]^+^, have also been prepared in high RCYs (>95%) at ambient temperature, by the addition of a solution of ^64^Cu^2+^ to solutions containing DP^Ph/Tol^-PSMAt bioconjugate. For both ^99m^Tc and ^64^Cu radiotracers, two copies of a targeting peptide are incorporated into a molecular radiotracer, potentially enhancing the target receptor affinity of this class of radiotracer. Other tracers that incorporate multiple copies of a targeting peptide have demonstrated increased target tissue accumulation relative to their monomeric homologues.^30−32^ The ability of these conjugates to complex both ^64^Cu and ^99m^Tc enables their use in both PET and SPECT molecular imaging.

We postulated that, by modifying the substituents of the (diaryl)phosphine ligands, an increase in the donor capacity of diphosphine–peptide groups could be achieved, which would improve the radiolabeling efficiency. IR spectroscopic measurements of [Mo(CO)_4_L] (L = bidentate diphosphine) compounds, in which model DP^Ph^-NH-MOE and DP^Tol^-NH-MOE ligands coordinate to Mo, indicated a modest but significant increase in the donor capacity of DP^Tol^-NH-MOE compared with that of DP^Ph^-NH-MOE. In ^99m^Tc radiolabeling kit-based reactions, the increased donor strength of DP^Tol^-MOE indeed resulted in higher RCYs for [^99m^TcO_2_(DP^Tol^-PSMAt)_2_]^+^ compared with [^99m^TcO_2_(DP^Ph^-PSMAt)_2_]^+^ at both ambient temperature and 100 °C. This observed statistically significant increase in the RCY of 6–8% was modest. For ^64^Cu radiolabeling reactions, we did not observe differences in the RCYs between DP^Ph^ and DP^Tol^ derivatives (both >95%).

For clinical adoption in radiopharmacies, radiolabeling reactions of receptor-targeted tracers need to provide near-quantitative RCYs (>95%) at relatively low amounts of ligand. Achieving near-quantitative RCY obviates time-consuming purification steps to remove unreacted radiometal from the desired radiotracer. Additionally, in such clinical formulations, the excess of ligand is typically *not* removed from the labeled radiotracer and is administered to patients along with the radiotracer. If present in very high amounts, this excess ligand can compete with the radiotracer for binding to target receptors *in vivo*, compromising the diagnostic imaging scans.

In this context, seemingly incremental increases in the RCY of a tracer can influence whether or not a radiotracer is suitable for routine radiopharmaceutical production and clinical adoption. The increased ^99m^Tc RCY afforded by the DP^Tol^ derivative is important in determining the potential clinical utility of this new radiolabeling platform. Here, low amounts of diphosphine–peptide conjugate (110 nmol, 110–120 μg) were used in kit-based reactions, with radiolabeling conditions mimicking the typical radiopharmaceutical formulation protocols.

This is the first report detailing how the modification of phosphine substituents can improve the efficiency of radiolabeling reactions in a pharmaceutical context. Our results suggest that this is a viable strategy for increasing RCYs of ^99m^Tc compounds based on diphosphines. Further derivatization of this platform, for example, the use of alternative aryl substituents or the use of aliphatic substituents, could further improve ^99m^Tc radiolabeling efficiencies.

The new ^99m^Tc and ^64^Cu diphosphine-PSMAt radiotracers possess favorable properties for use as receptor-targeted imaging agents. All of the radiotracers exhibit requisite high stability when incubated in human serum, with either no or low dissociation of the radiometal from the diphosphine–peptide conjugate over 24 h. The measured partition coefficients indicate that these radiotracers are comparatively hydrophilic, with all log *D*_OCT/PBS_ values lower than −2.0, despite the presence of eight aromatic groups in each of these radiotracers. The hydrophilicity in receptor-targeted tracers is generally preferred; hydrophobic radiotracers often accumulate and are retained in off-target organs such as the liver and intestines. Indeed, our preliminary SPECT/CT imaging studies show that both [^99m^TcO_2_(DP^Ph^-PSMAt)_2_]^+^ and [^99m^TcO_2_(DP^Tol^-PSMAt)_2_]^+^ clear circulation rapidly, predominantly via a renal pathway (Figure 4). These properties are favorable for receptor-targeted imaging radiotracers because the low concentration of radioactivity in nontarget, healthy tissues contributes to high contrast images, allowing better delineation of diseased tissue. We are currently evaluating these new molecular ^99m^Tc and ^64^Cu radiotracers *in vitro* and *in vivo* in PSMA-expressing prostate cancer models.

The presence of two isomeric radiolabeled products for DP-peptide conjugates, *cis*-[^99m^TcO_2_(DP-peptide)_2_]^+^ and *trans*-[^99m^TcO_2_(DP-peptide)_2_]^+^, is potentially unfavorable. It is possible that, prior to any clinical application, cis and trans isomers would require separate evaluation to qualify that their target affinities, pharmacokinetics, and stabilities are biologically equivalent to each other. Interestingly, the PSMA-targeted PET imaging radiopharmaceutical, ^68^Ga-HBED-PSMA, consists of at least two distinguishable (and as yet undefined) chemical species.^33,34^ However, the biological profiles of each distinct ^68^Ga-HBED-PSMA species have not been elucidated, and this has not prevented its clinical adoption in prostate cancer clinical management. We have very recently prepared and isolated *cis*-[^99m^TcO_2_(DP-gly_2_)_2_]^+^ and *trans*-[^99m^TcO_2_(DP-gly_2_)_2_]^+^ isomeric complexes.^17^ In this study, the bioconjugate DP-gly_2_ also consists of an asymmetric bidentate diphosphine, with one phosphine derivatized with two glucose substituents and the other phosphine with two phenyl substituents. Importantly, *cis*-[^99m^TcO_2_(DP-gly_2_)_2_]^+^ and *trans*-[^99m^TcO_2_(DP-gly_2_)_2_]^+^ exhibited near-identical biodistribution and clearance properties in a healthy mouse model. We anticipate that *cis*-[^99m^TcO_2_(DP-peptide)_2_]^+^ and *trans*-[^99m^TcO_2_(DP-peptide)_2_]^+^ derivatives, which all exhibit very similar chromatographic behavior, are likely to possess near-identical biological properties.

Lastly, we have shown that the new diphosphine–peptide conjugates coordinate to both [TcO_2_]^+^ and [ReO_2_]^+^ motifs to yield isostructural complexes. The generator-produced, β^–^-emitting isotope, ^188^Re, has demonstrated efficacy in systemic radiotherapy of liver, skin, and bone cancers.^35,36^ The ability to prepare pairs of chemically and biologically analogous ^99m^Tc and ^188^Re molecular radiopharmaceuticals will allow the clinical development of economical generator-based, dual diagnostic/therapeutic or “theranostic” radiopharmaceuticals for receptor-targeted molecular treatments. In addition to further synthetic *in vitro* and *in vivo* biological evaluation of our new diphosphine technology and ^99m^Tc radiotracers in prostate cancer models, we are also undertaking exploratory ^188^Re radiolabeling experiments.

In summary, this diphosphine chelator platform enables the simple and versatile development of new molecular radiopharmaceuticals: it is facile to derivatize with amine-containing targeting moieties, it allows radiolabeling with SPECT (^99m^Tc), PET (^64^Cu), and likely radiotherapeutic isotopes (^188^Re), and phosphine substituents can be tuned to increase the chelator binding and potentially the lipophilicity/hydrophilicity.

## Experimental Section

General experimental considerations are included in the SI.

### Synthesis

NMR and HRMS data and spectra are included in the SI.

#### DP^Ph^

Diphenylphosphine (2.2 equiv, 5.04 mmol, 0.88 mL) was added to a solution of dichloromaleic anhydride (1 equiv, 2.42 mmol, 404 mg) in degassed diethyl ether (15 mL) to give a pale-yellow solution. Triethylamine (TEA; 2.2 equiv, 5.04 mmol, 0.7 mL) was added dropwise and the dark-yellow suspension stirred for 2 h at ambient temperature until a compact sludge had formed. The solids, which contained product, were isolated by filter cannula and washed with ice cold diethyl ether (3 × 10 mL). The crude product was redissolved in dichloromethane and passed through a silica plug, after which the solvent was removed under reduced pressure to yield a yellow solid. This product was recrystallized from chloroform/diethyl ether, furnishing crystalline yellow needles (391 mg, 838 μmol, 35%).

#### Bis(*p*-tolyl)phosphine

Bis(*p*-tolyl)chlorophosphine (1 equiv, 4.02 mmol, 0.9 mL) in degassed diethyl ether (5 mL) was added dropwise to a slurry of lithium aluminum hydride (3.2 equiv, 13.0 mmol, 494 mg) in degassed diethyl ether (20 mL) at 0 °C. The gray suspension was stirred first at 0 °C for 30 min and then at ambient temperature for 22 h. The reaction was quenched by the dropwise addition of degassed water (0.5 mL), then a degassed aqueous solution of sodium hydroxide [0.5 mL, 15% (w/v)], and finally degassed water (1.5 mL), all at 0 °C. The white precipitate was removed from the filtrate (that contained the product) by filter cannula. The precipitate was then washed with diethyl ether (2 × 10 mL), and these washes were combined with the filtrate. The resulting solution was dried on magnesium sulfate and reisolated by filter cannula, washing the magnesium sulfate with diethyl ether (2 × 10 mL) and combining the filtrate and washes. The solvent was removed under reduced pressure to yield the product as a clear liquid (593 mg, 2.77 mmol, 69%), which crystallized below 20 °C. When the reaction scale was doubled, the crude product was purified by distillation at 200 °C and 2.5 × 10^–1^ mbar.

#### DP^Tol^

TEA (2.2 equiv, 1.00 mmol, 0.14 mL) was added to a solution of bis(*p*-tolyl)phosphine (2.05 equiv, 0.996 mmol, 207 mg) in dry, degassed diethyl ether (3.0 mL). A solution of dichloromaleic anhydride (1.0 equiv., 0.471 mmol, 78.7 mg) in dry, degassed diethyl ether (1.6 mL) was added dropwise, which resulted in an immediate color change from a colorless to deep-red solution with an orange precipitate. Once the reaction had reached completion, as monitored by ^31^P{^1^H} NMR spectroscopy, the volatiles were removed under reduced pressure. The crude product was dissolved in ethyl acetate and passed through a silica plug eluting with ethyl acetate and concentrated to dryness. Residual bis(*p*-tolyl)phosphine was removed by dissolving the crude product in ethyl acetate (1 mL), followed by the addition of hexane (5 mL) to afford an orange precipitate. The supernatant was removed and the precipitate washed with further hexane (2 × 3 mL) to give the product (214 mg, 0.407 mmol, 86%) as an orange solid. Any residual oxidized DP^Tol^ could be removed by crystallizing DP^Tol^ from chloroform and diethyl ether.

#### [Mo(CO)_4_(DP^X^)] (X = Ph, Tol)

A solution of either DP^Ph^ (16 mg, 33 μmol) or DP^Tol^ (17 mg, 33 μmol) in dichloromethane (0.3 mL) was added to a suspension of (norbornadiene)tetracarbonylmolybdenum(0) (10 mg, 34 μmol) in dichloromethane (0.25 mL). The reaction was left at room temperature for either 20 h (DP^Tol^) or 3 days (DP^Ph^) until reaction completion, determined by *in situ*^31^P{^1^H} NMR spectroscopy. The solvent was removed *in vacuo* to yield a purple solid, which was azeotroped with toluene (1.5 mL) and then washed with hexane (2 mL). Finally, the solid was isolated by cannula filtration and dried to yield the product. [Mo(CO)_4_(DP^Ph^)]: 19 mg, 0.028 mmol, 84%, pale purple. [Mo(CO)_4_(DP^Tol^)]: 24 mg, 32.6 μmol, 99%, pale brown.

#### [MOE-NH_3_][Mo(CO)_4_(DP^X^-NH-MOE)]^−^ (X = Ph, Tol)

A solution of (2-methoxyethyl)amine (3 equiv, 6–7 mg) in dichloromethane (0.12 mL) was added to a solution of either [Mo(CO)_4_(DP^Ph^)] (18 mg, 26.7 μmol) or [Mo(CO)_4_(DP^Tol^)] (23 mg, 31.1 μmol) in dichloromethane (0.4 mL), leading to the immediate formation of [Mo(CO)_4_(DP^X^-NH-MOE)]^−^ (X = Ph, Tol), as determined by *in situ*^31^P{^1^H} NMR spectroscopy and a color change (to pale yellow/orange). The solvent was removed, and the resulting product was washed with hexane (0.6 mL). The solid was isolated by cannula filtration and dried. [MOE-NH_3_][Mo(CO)_4_(DP^Ph^-NH-MOE)]: 12 mg, 14.2 μmol, 53%. [MOE-NH_3_][Mo(CO)_4_(DP^Tol^-MOE)]: 9 mg, 10.2 μmol, 33%.

#### DP^Tol^-RGD

Under a stream of N_2_, DP^Tol^ (4.1 mg, ∼8 μmol) and *N*,*N*-diisopropylethylamine (DIPEA, 6 μL) were added to a solution of cyclic RGDfK peptide (4.6 mg, ∼8 μmol) in degassed *N*,*N*-dimethylformamide (DMF; 200 μL). The resulting dark-orange solution was left to react at ambient temperature for 20 min, resulting in a pale-orange solution. The reaction solution was applied to a reverse-phase C_18_ semipreparative HPLC column and purified by HPLC (method 5). An aqueous ammonium bicarbonate solution (0.125 M, 15 μL/mL elute) was added to fractions containing the desired product. These solutions were lyophilized to yield DP^Tol^-RGD (4.9 mg, 4.35 μmol, 57%) as a solid.

#### DP^Ph^-PSMAt and DP^Tol^-PSMAt

Under a stream of N_2_, DP^Ph^ or DP^Tol^ (4–5 mg, 1 equiv) in degassed DMF (100 μL) and Lys-((PEG)_4_-NH_2_)-uredo-Glu [Lys(PEG4)-CO-Glu; 4–6 mg, 1 equiv] in degassed DMF (100 μL) were combined and DIPEA (6 μL) was added. The solution was agitated at room temperature for 15–20 min. The reaction solution was then applied to a reverse-phase C_18_ semipreparative HPLC column and purified by HPLC (method 6). An aqueous ammonium bicarbonate solution (0.125 M, 15 μL/mL elute) was added to fractions containing the desired product. These solutions were lyophilized to yield either DP^Ph^-PSMAt or DP^Tol^-PSMAt (>60%).

#### [ReO_2_(DP^Ph^-PSMAt)_2_]^+^ and [ReO_2_(DP^Tol^-PSMAt)_2_]^+^

In initial experiments in which we monitored the reaction of [ReO_2_I(PPh_3_)_2_] with DP^X^-PSMAt (X = Ph, Tol) species by LC–MS, we observed that an *excess* of [ReO_2_I(PPh_3_)_2_] complex favored formation of the desired products. Therefore, in subsequent experiments, we elected to use only a single equivalent of the DP-peptide ligands compared to [ReO_2_I(PPh_3_)_2_]. A solution of [ReO_2_I(PPh_3_)_2_] (∼14–17 mg, 16.6–19.6 μmol, 1 equiv) in DMF (200 μL) was combined with a solution of either DP^Ph^-PSMAt (20.2 mg, 19.6 μmol, 1 equiv) or DP^Tol^-PSMAt (18.2 mg, 16.7 μmol, 1 equiv) and DIPEA (9 μL) in DMF (300 μL). The resulting dark-brown solution was left to react at room temperature for 2–3 h. The reaction solution was applied to a reverse-phase C_18_ semipreparative HPLC column and purified by HPLC (method 6). The highest-purity fractions containing the desired product were lyophilized to yield [ReO_2_(DP^Ph^-PSMAt)_2_]^+^ (7.6 mg, 3.3 μmol, 34%) and [ReO_2_(DP^Tol^-PSMAt)_2_]^+^ (7.5 mg, 3.1 μmol, 38%) as solids.

### Radiolabeling and Radiotracer Characterization

#### Kit Preparation

An aqueous stock solution was prepared containing the required amounts of sodium bicarbonate, tin chloride, and sodium tartrate. The pH was adjusted to 8–8.5 by the dropwise addition of an aqueous solution of sodium hydroxide (0.1 M). Aliquots of the stock solution were mixed with the required amount of DP^Ph^-PSMAt or DP^Tol^-PSMAt [dissolved in a mixture of water/ethanol (50%/50%)] to form the kit solutions outlined in Table 4, which were immediately frozen and lyophilized using a freeze-dryer. The lyophilized kits were stored in a freezer prior to use.

**Table 4:** Lyophilized Kit Formulations for DP^Ph^-PSMAt and DP^Tol^-PSMAt for Radiolabeling

	kit composition			
	DP^Ph^-PSMAt kit		DP^Tol^-PSMAt kit	
component	amount (μmol)	mass (mg)	amount (μmol)	mass (mg)
DP^Ph^-PSMAt	0.11	0.11		
DP^Tol^-PSMAt			0.11	0.12
SnCl_2_·2H_2_O	0.11	0.03	0.11	0.03
sodium tartrate	1.15	0.26	1.15	0.26
NaHCO_3_	10.71	0.90	10.71	0.90

#### Radiolabeling of DP^Ph^-PSMAt and DP^Tol^-PSMAt with ^99m^TcO_4_^–^

DP^Ph^-PSMAt and DP^Tol^-PSMAt were radiolabeled with generator-produced ^99m^TcO_4_^–^ (200 MBq) in a saline solution (500 μL, 0.9% NaCl in water, w/v), using the lyophilized kits described in Table 4. The radiolabeling reaction mixtures were either left to react at ambient temperature (∼22 °C) for 5 min or heated at 100 °C for 5 min. Aliquots were analyzed by iTLC and analytical C_18_ HPLC to determine the RCYs. The species were attributed as [^99m^TcO_2_(DP^Ph^-PSMAt)_2_]^+^ eluted at 11.0–12.5 min and [^99m^TcO_2_(DP^Tol^-PSMAt)_2_]^+^ eluted at 12.5–14.0 min.

Two separate iTLC analyses were undertaken, to enable quantification of ^99m^Tc colloids and unreacted ^99m^TcO_4_^–^ and [^99m^TcO_2_(DP-PSMAt)_2_]^+^. To quantify the amounts of unreacted ^99m^TcO_4_^–^, acetone was used as a mobile phase. *R*_f_ values: ^99m^TcO_4_^–^ > 0.9, ^99m^Tc colloids < 0.1, and [^99m^TcO_2_(DP-PSMAt)_2_]^+^ < 0.1. To quantify ^99m^Tc-colloid formation, a 1:1 mixture of methanol and a 2 M aqueous ammonium acetate solution was used as a mobile phase: ^99m^TcO_4_^–^ > 0.9, ^99m^Tc colloids < 0.1, and [^99m^TcO_2_(DP-PSMAt)_2_]^+^ > 0.9.

For *in vitro* and *in vivo* studies, these kit-based reaction solutions were further purified. Solutions of either [^99m^TcO_2_(DP^Ph^-PSMAt)_2_]^+^ or [^99m^TcO_2_(DP^Tol^-PSMAt)_2_]^+^ prepared from kits were applied to a SE-HPLC column (method 7), using an aqueous mobile phase of phosphate-buffered saline. Fractions containing either [^99m^TcO_2_(DP^Ph^-PSMAt)_2_]^+^ or [^99m^TcO_2_(DP^Tol^-PSMAt)_2_]^+^ (>95% radiochemical purity) eluted at 10–12 min. Other reaction components, including unreacted starting materials and impurities, also eluted at distinct retention times: unlabeled DP^Ph^-PSMAt ligand eluted at 16–17 min, unlabeled DP^Tol^-PSMAt eluted at 27–28 min, ^99m^TcO_4_^–^ eluted at 14–15 min, and ^99m^Tc colloid was trapped on the column.

#### Preparation of [^99g^TcO_2_(DP^Ph^-PSMAt)_2_]^+^ and [^99g^TcO_2_(DP^Tol^-PSMAt)_2_]^+^

The ^99g^Tc(V) precursor [N^*t*^Bu_4_][^99g^TcOCl_4_] was prepared following a previously described method.^37^ A solution of either DP^Ph^-PSMAt or DP^Tol^-PSMAt (1.0 mg, ∼1 μmol, 2 equiv) dissolved in methanol (300 μL, degassed) was combined with a solution of [N^*t*^Bu_4_][^99g^TcOCl_4_] (0.25 mg, 0.46 μmol, 1 equiv) in methanol (50 μL). The resulting pale-yellow solution was left to react at ambient temperature for 15 min. An aliquot was then analyzed by LC-MS-ESI^+^ (method 8) and HR-ESI-MS.

#### [^99g^TcO_2_(DP^Ph^-PSMAt)_2_]^+^

LR-MS-ESI (*m*/*z*): [M + H]^2+^ 1099.0 (calcd for C_102_H_125_N_8_O_32_P_4_Tc 1098.5), [M + Na]^2+^ 1110.0 (calcd for C_102_H_124_N_8_O_32_P_4_TcNa 1109.5), [M + K]^2+^ 1117.7 (calcd for C_102_H_124_N_8_O_32_P_4_TcK 1117.5), [M + 2H]^3+^ 732.7 (calcd for C_102_H_126_N_8_O_32_P_4_Tc 732.7), [M + H + K]^3+^ 745.2 (calcd for C_102_H_126_N_8_O_32_P_4_TcK 745.3).

#### [^99g^TcO_2_(DP^Tol^-PSMAt)_2_]^+^

LR-MS-ESI (*m*/*z*): [M + H]^2+^ 1155.0 (calcd for C_110_H_141_N_8_O_32_P_4_Tc 1154.5), [M + Na]^2+^ 1165.8 (calcd for C_110_H_140_N_8_O_32_P_4_TcNa 1165.5), [M + K]^2+^ 1173.8 (calcd for C_110_H_140_N_8_O_32_P_4_TcK 1173.5), [M + 2H]^3+^ 770.3 (calcd for C_110_H_142_N_8_O_32_P_4_Tc 770.0), [M + H + K]^3+^ 782.8 (calcd for C_110_H_141_N_8_O_32_P_4_TcK 782.7).

#### log _7.4_*D* of [^99m^TcO_2_(DP^Ph^-PSMAt)_2_]^+^ and [^99m^TcO_2_(DP^Tol^-PSMAt)_2_]^+^

The following procedure was carried out in triplicate. A solution containing either [^99m^TcO_2_(DP^Ph^-PSMAt)_2_]^+^ or [^99m^TcO_2_(DP^Tol^-PSMAt)_2_] (0.25 MBq in 1 μL) was combined with phosphate-buffered saline (pH 7.4, 500 μL) and octanol (500 μL), and the mixture was agitated for 30 min. The mixture was then centrifuged (10 000 rpm, 10 min), and aliquots of octanol and aqueous phosphate-buffered saline solution were analyzed for radioactivity using a γ counter. log _7.4_*D* of [^99m^TcO_2_(DP^Ph^-PSMAt)_2_]^+^ = −2.45 ± 0.20; log _7.4_*D* of [^99m^TcO_2_(DP^Tol^-PSMAt)_2_]^+^ = −2.08 ± 0.30.

#### Serum Stability of [^99m^TcO_2_(DP^Ph^-PSMAt)_2_]^+^ and [^99m^TcO_2_(DP^Tol^-PSMAt)_2_]^+^

A solution containing either [^99m^TcO_2_(DP^Ph^-PSMAt)_2_]^+^ or [^99m^TcO_2_(DP^Tol^-PSMAt)_2_]^+^ (100 μL, 80 MBq) was added to filtered human serum (Sigma-Aldrich, 900 μL) and incubated at 37 °C. At 1, 4, and 24 h, aliquots were taken. Each aliquot (300 μL) was treated with ice-cold acetonitrile (300 μL) to precipitate and remove serum proteins. Acetonitrile in the supernatant was then removed by evaporation under a stream of N_2_ gas (40 °C, 30 min). This final supernatant solution was then analyzed by reverse-phase analytical HPLC (method 2).

#### *In Vivo* Imaging of [^99m^TcO_2_(DP^Ph^-PSMAt)_2_]^+^ and [^99m^TcO_2_(DP^Tol^-PSMAt)_2_]^+^ in Healthy Mice

Animal imaging studies were ethically reviewed and carried out in accordance with the Animals (Scientific Procedures) Act 1986 (ASPA) U.K. Home Office regulations governing animal experimentation. Mice were purchased from Charles River (Margate, U.K.). A male SCID Beige mouse (approximately 3 months old, *n* = 1) was anaesthetized [2.5% (v/v) isofluorane, 0.8–1.0 L/min O_2_ flow rate] and injected intravenously via the tail vein with [^99m^TcO_2_(DP^Ph^-PSMAt)_2_]^+^ (100 μL, 26 MBq, >99% RCP, 0–5 μg PSMAt peptide in phosphate-buffered saline) or [^99m^TcO_2_(DP^Tol^-PSMAt)_2_]^+^ (160 μL, 30 MBq, >99% RCP, 0–5 μg PSMAt peptide in phosphate-buffered saline), followed immediately by CT acquisition and SPECT scanning. Following completion of the scan, mice were culled and urine was collected for HPLC analysis. For the sake of time efficiency during *in vivo* experimentation, we elected to use a shorter analytical HPLC method (HPLC method 2) to determine the purity of the radiotracers and subsequently to analyze the urine samples.

#### ^64^Cu Radiolabeling of DP^Ph^-PSMAt and DP^Tol^-PSMAt

^64^Cu was produced by a ^64^Ni(p,n)^64^Cu nuclear reaction on a CTI RDS 112 11 MeV cyclotron and purified to give ^64^Cu^2+^ in 0.1 M HCl solutions used for radiolabeling.^38,39^ The ^64^Cu^2+^ solutions (in 0.1 M HCl) were dried under a flow of N_2_ with heating at 100 °C, and the residue was redissolved in an ammonium acetate solution (0.1 M, pH 7). An aliquot of an ammonium acetate solution containing ^64^Cu^2+^ (10 MBq, 50–100 μL) was added to either DP^Ph^-PSMAt (50 μg) or DP^Tol^-PSMAt (50 μg) dissolved in an aqueous ammonium acetate (0.1 M) to give a final radiolabeling solution of 200 μL volume. The radiolabeling mixtures were left to react at ambient temperature (∼22 °C) for 20 min. Aliquots were analyzed by iTLC and analytical HPLC to determine the RCYs. By C_18_ analytical HPLC (method 2), the species attributed as [^64^Cu(DP^Ph^-PSMAt)_2_]^+^ eluted at 12.0–13.0 min; [^64^Cu(DP^Tol^-PSMAt)_2_]^+^ eluted at 13.5–14.5 min; unreacted ^64^Cu^2+^ eluted with the solvent front at 2.0–3.5 min.

iTLC analysis was undertaken to enable the quantification of unreacted ^64^Cu^2+^ and [^64^Cu(DP-PSMAt)_2_]^+^. Citrate buffer (0.1 M, pH 5) was used as a mobile phase. *R*_f_ values: unreacted ^64^Cu^2+^ > 0.9, and [^64^Cu(DP-PSMAt)_2_]^+^ < 0.1.

#### Preparation of [Cu(DP^Ph^-PSMAt)_2_]^+^ and [Cu(DP^Tol^-PSMAt)_2_]^+^

A solution of either DP^Ph^-PSMAt or DP^Tol^-PSMAt (1.0 mg, ∼1 μmol, 2 equiv) in saline (500 μL) was added to a solution of [Cu(MeCN)_4_][PF_6_] (170–180 μg, ∼0.5 μmol, 1 equiv) in acetonitrile (dry, deoxygenated, 500 μL). The reaction mixture was left to react at ambient temperature for 60 min. The product was isolated by semipreparative HPLC (method 6), lyophilizing the product fractions eluting at either ∼46–47 min ([Cu(DP^Ph^-PSMAt)_2_]^+^) or 56–57 min ([Cu(DP^Tol^-PSMAt)_2_]^+^). Yield = 30–40%. Aliquots of [Cu(DP^Ph^-PSMAt)_2_]^+^ or [Cu(DP^Tol^-PSMAt)_2_]^+^ were analyzed by analytical HPLC (method 2).

#### log _7.4_*D* of [^64^Cu(DP^Ph^-PSMAt)_2_]^+^ and [^64^Cu(DP^Tol^-PSMAt)_2_]^+^

The following procedure was carried out in triplicate. A solution containing either [^64^Cu(DP^Ph^-PSMAt)_2_]^+^ or [^64^Cu(DP^Tol^-PSMAt)_2_] (0.5 MBq in 20 μL) was combined with phosphate-buffered saline (pH 7.4, 480 μL) and octanol (500 μL), and the mixture was agitated for 30 min. The mixture was then centrifuged (10 000 rpm, 10 min), and aliquots of octanol and aqueous phosphate-buffered saline were analyzed for radioactivity using a γ counter. log _7.4_*D* of [Cu(DP^Ph^-PSMAt)_2_]^+^ = −3.30 ± 0.03; log _7.4_*D* of [Cu(DP^Tol^-PSMAt)_2_]^+^ = −3.01 ± 0.06.

#### Serum Stability of [^64^Cu(DP^Ph^-PSMAt)_2_]^+^ and [^64^Cu(DP^Tol^-PSMAt)_2_]^+^

A sample of [Cu(DP^Ph^-PSMAt)_2_]^+^ (>99.0% RCP, 1.7 MBq, 5 μg DP^Ph^-PSMAt ligand) or ^64^Cu-DP^Tol^-PSMAt (>99.0% RCP, 1.7 MBq, 5 μg DP^Tol^-PSMAt ligand) in an aqueous solution of ammonium acetate (20 μL, 0.1 M) was added to filtered human serum from a healthy volunteer (180 μL) and incubated at 37 °C. At 1, 4, and 24 h, aliquots were taken. Each aliquot (300 μL) was treated with ice-cold acetonitrile (300 μL) to precipitate and remove serum proteins. Acetonitrile in the supernatant was then removed by evaporation under a stream of N_2_ gas (40 °C, 30 min). The final solution was then analyzed by reverse-phase analytical HPLC (method 2).

## Supporting Information Available

General experimental and instrumentation details, NMR and ESI-MS, ^31^P{^1^H} NMR spectrum simulations, IR spectroscopy, and HPLC (PDF)
