# Supplementary material for: Versatile Diphosphine Chelators for Radiolabeling Peptides with 99mTc and 64Cu
Source: Inorg Chem. 2023 Mar 27;62(50):20608–20. doi: 10.1021/acs.inorgchem.3c00426 (PMC10731650; doi:10.1021/acs.inorgchem.3c00426)
Supplement: Supplementary file 1 — ic3c00426_si_001.pdf [file ic3c00426_si_001.pdf]

Supporting Information

Versatile diphosphine chelators for radiolabelling peptides with ^{99m}Tc and ^{64}Cu

Ingebjørg N. Hungnes,[1] Truc Thuy Pham,[1] Charlotte Rivas,[1] James A. Jarvis,[2] Rachel E. Nuttall,[1,3] Saul M. Cooper,[4] Jennifer D. Young,[1] Philip J. Blower,[1] Paul G. Pringle,[3] Michelle T. Ma[1]**

[1] King's College London, School of Biomedical Engineering and Imaging Sciences, 4th Floor Lambeth Wing, St Thomas' Hospital, London, United Kingdom, SE1 7EH

[2] King's College London, Randall Centre of Cell and Molecular Biophysics and Centre for Biomolecular Spectroscopy, London, United Kingdom, SE1 9RT

[3] University of Bristol, School of Chemistry, Cantock's Close, Bristol, United Kingdom, BS8 1TS

[4] Imperial College London, Department of Chemistry, Molecular Sciences Research Hub, London, United Kingdom, W12 0BZ

**michelle.ma@kcl.ac.uk, paul.pringle@bristol.ac.uk*

1. **General experimental considerations** 4

2. **NMR and HR-MS-ESI characterisation of reported compounds** 6

3. **NMR Spectra** 10

Figure S1: ^{31}P{^{1}H} NMR spectra of DP^{Tol} and precursors 10
Figure S2: ^{31}P{^{1}H} NMR of DP^{Ph} and DP^{Tol}. 10
Figure S3: ^{31}P{^{1}H} NMR of [Mo(CO)$_4$(DP^{Ph})] and [Mo(CO)$_4$(DP^{Tol})]. 11
Figure S4: ^{31}P{^{1}H} NMR spectra of [Mo^0(CO)$_4$(DP^{Ph}-NHR)]$^-$ and [Mo^0(CO)$_4$(DP^{Tol}-NHR)]$^-$ 11
Figure S5: ^{13}C NMR of DP^{Ph}, (RNH$_3$)[Mo^0(CO)$_4$(DP^{Ph})] and (RNH$_3$)[Mo^0(CO)$_4$(DP^{Ph}-NHR)] 12
Figure S6: ^{13}C NMR of DP^{Tol}, [Mo^0(CO)$_4$(DP^{Tol})] and (RNH$_3$)[Mo^0(CO)$_4$(DP^{Tol}-NHR)] 12
Figure S7: ^{13}C NMR of [Mo^0(CO)$_4$(DP)] and (RNH$_3$)[Mo^0(CO)$_4$(DP-NHR)] (DP = DP^{Ph} or DP^{Tol}). 13
Figure S8: ^{1}H NMR of DP^{Tol}-PSMAt and [Cu(DP^{Tol}-PSMAt)$_2$]$^+$. 13
Figure S9: Structure of [Cu(DP$^{Ph/Tol}$-PSMAt)$_2$]$^+$ and o DP^{Ph}-PSMAt and DP^{Tol}-PSMAt ligands 14
Figure S10: ^{1}H NMR spectrum of DP^{Tol} 15
Figure S11: ^{13}C NMR spectrum of DP^{Tol} 15
Figure S12: Magnifications of ^{13}C NMR spectrum of DP^{Tol} 16
Figure S13: ^{1}H-^{13}C HSQC NMR spectrum of DP^{Tol} 17
Figure S14: ^{31}P{^{1}H} NMR spectrum of DP^{Tol} 17
Figure S15: ^{1}H NMR spectrum of [Mo(CO)$_4$(DP^{Ph})]. 18
Figure S16: ^{1}H NMR spectrum of [Mo(CO)$_4$(DP^{Tol})] 18
Figure S17: ^{1}H NMR spectrum of (RNH$_3$)[Mo(CO)$_4$(DP^{Ph}-NHR)] 19
Figure S18: ^{1}H-^{1}H-TOCSY NMR spectrum of (RNH$_3$)[Mo(CO)$_4$(DP^{Ph}-NHR)] 19
Figure S19: ^{1}H NMR spectrum of (RNH$_3$)[Mo(CO)$_4$(DP^{Ph}-NHR)] 20
Figure S20: ^{1}H-^{1}H-TOCSY NMR spectrum of (RNH$_3$)[Mo(CO)$_4$(DP^{Tol}-NHR)] 20
Figure S21: ^{1}H NMR spectrum of DP^{Tol}-RGD 21
Figure S22: ^{13}C NMR spectrum of DP^{Tol}-RGD 21
Figure S23: ^{31}P{^{1}H} NMR spectrum of DP^{Tol}-RGD 22
Figure S24: ^{1}H-^{1}H-TOCSY and ^{1}H-^{13}C HSQC NMR spectra of DP^{Tol}-RGD 23

Figure S25: ^{1}H NMR spectrum of DP^{Ph}-PSMAt 24
Figure S26: ^{13}C NMR spectrum of DP^{Ph}-PSMAt 24
Figure S27: ^{1}H-^{1}H-TOCSY and ^{1}H-^{13}C HSQC NMR spectrum of DP^{Ph}-PSMAt 25
Figure S28: ^{1}H NMR spectrum of DP^{Tol}-PSMAt........ 26
Figure S29: ^{13}C NMR spectrum of DP^{Tol}-PSMAt 26
Figure S30: ^{1}H-^{1}H-TOCSY and ^{1}H-^{13}C HSQC NMR spectrum of DP^{Tol}-PSMAt........ 27
Figure S31: ^{1}H NMR spectrum of $[ReO_2(DP^{Ph}\text{-PSMAt})_2]^+$ 28
Figure S32: ^{13}C NMR spectrum of $[ReO_2(DP^{Ph}\text{-PSMAt})_2]^+$ 28
Figure S33: ^{1}H-^{1}H-TOCSY NMR spectrum of $[ReO_2(DP^{Ph}\text{-PSMAt})_2]^+$ 29
Figure S34: ^{1}H-^{13}C HSQC NMR spectrum of $[ReO_2(DP^{Ph}\text{-PSMAt})_2]^+$ 29
Figure S35: ^{1}H NMR spectrum of $[ReO_2(DP^{Tol}\text{-PSMAt})_2]^+$........ 30
Figure S36: ^{13}C NMR spectrum of $[ReO_2(DP^{Tol}\text{-PSMAt})_2]^+$........ 30
Figure S37: ^{1}H-^{1}H-TOCSY NMR spectrum of $[ReO_2(DP^{Tol}\text{-PSMAt})_2]^+$ 31
Figure S38: ^{1}H-^{13}C HSQC NMR spectrum of $[ReO_2(DP^{Tol}\text{-PSMAt})_2]^+$........ 31
Figure S39: ^{1}H NMR spectrum of $[Cu(DP^{Ph}\text{-PSMAt})_2]^+$ 32
Figure S40: ^{1}H-^{1}H-TOCSY NMR spectrum of $[Cu(DP^{Ph}\text{-PSMAt})_2]^+$. 32
Figure S41: ^{1}H-^{1}H-TOCSY NMR spectrum of $[Cu(DP^{Tol}\text{-PSMAt})_2]^+$........ 33

4. **$^{31}P\{^{1}H\}$ NMR spectrum simulations** 34
Figure S42: Experimental and simulated $^{31}P\{^{1}H\}$ NMR spectra of cis/trans-$[^{nat}ReO_2(DP^{Tol}\text{-PSMAt})_2]^+$ 34
Figure S43: Experimental and simulated $^{31}P\{^{1}H\}$ NMR spectra of cis/trans-$[^{nat}ReO_2(DP^{Ph}\text{-PSMAt})_2]^+$ 35

5. **High resolution mass spectrometry results** 36
Figure S44: HRMS-ESI+ spectrum of DP^{Tol}........ 36
Figure S45: HRMS-ESI- spectrum of $(RNH_3)[Mo(CO)_4(DP^{Ph}\text{-NHR})]$ (R = $-CH_2CH_2OCH_3$). 36
Figure S46: HRMS-ESI- spectrum of of $(RNH_3)[Mo(CO)_4(DP^{Tol}\text{-NHR})]$ (R = $-CH_2CH_2OCH_3$). 37
Figure S47: HRMS-ESI+ spectra of DP^{Tol}-RGD........ 37
Figure S48: HRMS-ESI+ spectra of DP^{Ph}-PSMAt. 38
Figure S49: HRMS-ESI+ spectra of DP^{Tol}-PSMAt........ 38
Figure S50: HRMS-ESI+ spectrum of $[ReO_2(DP^{Ph}\text{-PSMAt})_2]^+$. 39
Figure S51: HRMS-ESI+ spectrum of $[ReO_2(DP^{Tol}\text{-PSMAt})_2]^+$........ 40
Figure S52: HRMS-ESI+ spectrum of $[CuO_2(DP^{Ph}\text{-PSMAt})_2]^+$. 41
Figure S53: HRMS-ESI+ spectrum of $[CuO_2(DP^{Tol}\text{-PSMAt})_2]^+$........ 42

6. **Infra-red spectroscopy** 43
Figure S54: Infra-red spectra of $[Mo(CO)_4(nbd)]$, $[Mo(CO)_4(DP^{Ph/Tol})]$ and $[Mo(CO)_4(DP^{Ph/Tol}\text{-NHR})]^-$ 43

7. **HPLC and low-resolution LCMS-ESI results** 44
Figure S55: Radio-HPLC traces of $[^{99m}TcO_2(DP^{Ph}\text{-PSMAt})_2]^+$ and $[^{99m}TcO_2(DP^{Tol}\text{-PSMAt})_2]^+$ 44
Figure S56: Radio-LCMS of $[^{99m/99}TcO_2(DP^{Tol}\text{-RGD})_2]^+$. 44
Figure S57: UV chromatogram and mass spectrum of $[MO_2(DP^{Ph}\text{-PSMAt})_2]^+$)
and $[MO_2(DP^{Tol}\text{-PSMAt})_2]^+$ (M = ^{99g}Tc/^{nat}Re). 45

8. **References** 46

1. General experimental considerations

All chemicals were supplied by Sigma-Aldrich or Fisher Scientific if not otherwise specified. Cyclic RGDfK pentapeptide and PSMAt dipeptide (Lys((PEG)$_4$)-uredo-Glu) were purchased from Peptide Synthetics (Hampshire, UK). [^{99m}Tc]pertechnetate in saline, eluted from a UTK generator (Curium), was supplied by the Guy's and St Thomas' Hospital Nuclear Medicine Services, London. Ammonium [^{99}Tc]pertechnetate was supplied by Amersham International plc (Amersham UK, 1991). Synthesis of non-peptidic phosphines and molybdenum complexes was carried out under an inert atmosphere using standard Schlenk line (nitrogen) and glove-box (argon) techniques. Acetonitrile, toluene, dichloromethane, tetrahydrofuran, hexane and diethyl ether were collected from a Grubbs-type solvent system and deoxygenated by three successive freeze–pump–thaw cycles and stored over 4 Å molecular sieves. Deuterated dichloromethane and chloroform were dried over activated 4 Å molecular sieves and deoxygenated by three successive freeze–pump–thaw cycles. All commercial reagents were purchased from Sigma-Aldrich and used as supplied unless otherwise stated. Dichloromaleic anhydride was sublimated under reduced pressure (10^{-1} mbar) at 120 ˚C prior to use.

Nuclear magnetic resonance (NMR) data was acquired on Bruker Avance III 400 spectrometer equipped with a QNP probe or a Bruker Avance III 700 spectrometer equipped with an AVIII console and a quadruple-resonance QCI cryoprobe or Jeol ECP (Eclipse) 300, Jeol ECS 400 and Bruker cryo 500 spectrometers. Chemical shifts were referenced to residual solvent (^{1}H and ^{13}C) or 85% H_3PO_4 (^{31}P{^{1}H}). Acquired NMR spectra were analysed using TopSpin® 3.5pl7 or 4.0.3 (Biospin Ltd) or MestReNova 11.0.4 (Mestrelab Research SL). Infrared spectra recorded on a PerkinElmer FT-IR. Spectra were recorded in dichloromethane at a compound concentration of approximately 1 mg mL^{-1} at room temperature. High resolution mass spectrometry (MS) was performed by the King's College London Mass Spectrometry Facilities, using a high resolution Thermo Exactive mass spectrometer in positive electrospray mode. Samples were infused to the ion source at a rate of 10 ul/min using a syringe pump. Low resolution MS data was collected with an Advion Expression Compact Mass Spectrometer, using an ESI probe. Mass spectra of non-peptidic phosphine and molybdenum compounds were acquired by the University of Bristol Mass Spectrometry Service on a Synapt G2S (IMS-Q-TOF, ES+; Waters) or Orbitrap Elite (ESI; Thermo Scientific) spectrometer.

Instant thin layer chromatography (iTLC) used iTLC SGI0001 strips (Varian Medical Systems, Crawley, UK). The iTLC plates were scanned with a Perkin Elmer Storage Phosphor System (Cyclone) or a LabLogic miniScan TLC reader equipped with Laura software.

High performance liquid chromatography (HPLC) was carried out on an Agilent 1200 HPLC system with Laura software, a Rheodyne sample loop (200 µL) and ultraviolet (UV) spectroscopic detection at 214, 220 nm, 254 nm or 280 nm. HPLC methods 5 and 6 were undertaken on an Agilent Prostar HPLC with prepLC software (Agilent), a Rheodyne sample loop (2 mL) and UV spectroscopic detection at 214 and 254 nm. HPLC-MS was carried out on an Agilent 1260 Infinity II HPLC system coupled to an Advion Expression Compact Mass Spectrometer. The HPLC was attached to a LabLogic Flow-Count detector with a sodium iodide probe (B-FC-3200) for radiation detection. Semi-preparative (9.4 x 250 mm, 5 µm) and analytical (4.6 x 150 mm, 5 µm) Agilent Zorbax Eclipse XDB-C18 columns were used for reverse-phase chromatography with purified water (A) and acetonitrile (B), both containing trifluoroacetic acid (TFA), acetic acid (AA) or formic acid (FA) as outlined in the methods, as mobile phases. A BioSep™ SEC-s2000 LC Column (300 x 7.8 mm, 5 µm, 145 Å, Phenomenex®) was used for size-exclusion chromatography (SEC), using aqueous phosphate buffered saline solution as mobile phase. **Method 1** (C_{18} semi-preparative, 0.005% TFA, 214 nm): 100 min, 1% min^{-1} linear increase from 100% A to 0% A, flow rate of 3 mL min^{-1}. **Method 2** (C_{18} analytical, 0.1% TFA, 220 nm): 20 min, 5% min^{-1} linear increase from 100% A to 0% A, flow rate of 1

mL min^{-1}. **Method 3** (C_{18} semi-preparative, 0.005% TFA, 220 nm): 190 min, 0.5% min^{-1} linear increase from 95% A to 0% A, flow rate of 3 mL min^{-1}. **Method 4** (C_{18} analytical, 0.1% TFA, 220 nm): 55 min, 2.5% min^{-1} linear increase from 100% A to 75% A, then 0.33% min^{-1} linear increase from 75% A to 60% A, flow rate of 1 mL min^{-1}. **Method 5** (C_{18} semi-preparative, 0.005% TFA, 214 and 254 nm): 90 min, isocratic flow at 95% A for 5 min, then 0.93% min^{-1} linear increase from 95% A to 25% A, followed by 2.5% min^{-1} linear increase from 25% A to 0% A, flow rate of 3 mL min^{-1}. **Method 6** (C_{18} semi-preparative, 0.01% AA, 214 and 254 nm): Same gradient and flow as Method 5. **Method 7** (SEC, phosphate buffered saline, 280 nm): Isocratic gradient, flow rate of 1 mL min^{-1}. **Method 8** (C_{18} analytical LCMS, 0.1% FA, 254 nm): 35 min, isocratic flow at 100% A for 5 min, then 3% min^{-1} linear increase from 100% A to 5% A, flow rate of 1 mL min^{-1}. **Method 9** (C_{18} semi-preparative, 0.1% TFA, 214 nm): 70 min, 1% min^{-1} linear increase from 100% A to 30% A, flow rate of 3 mL min^{-1}. **Method 10** (C_{18} analytical, 0.1% TFA, 220 nm): 80 min, 1% min^{-1} linear increase from 100% A to 20% A, flow rate of 1 mL min^{-1}.

SPECT/CT imaging was accomplished using a pre-clinical nanoScan SPECT/CT Silver Upgrade instrument (Mediso), calibrated for technetium-99m. The SPECT scans were acquired by helical SPECT (4-head scanner with 4 × 9 pinhole collimators), and CT scans by helical CT (55 kVP X-ray source, 1000 ms exposure time in 180 projections over 9 min). 1.0 mm pinhole collimators were used. SPECT acquisition was done in eight segments: the first segment was acquired at 15-30 min post injection (frame time of 12s; 9 min acquisition time), followed by seven imaging segments of 30 min each (frame time of 33 s; 24.75 min acquisition time) up until 4 h post-injection. At the end of the imaging procedure, the mouse was culled by cervical dislocation and a sample of the urine analysed by analytical HPLC (method 2). SPECT images were reconstructed at 0.3 mm isotropic voxel size with the HiSPECT (Scivis GmbH) reconstruction software package using standard reconstruction with 35% smoothing and nine iterations. The CT and SPECT images were further processed and analysed using VivoQuant software (inviCRO, USA).

2. NMR and HR-MS-ESI characterisation of reported compounds

DP^{Ph}: ^{1}H NMR (400 MHz, acetonitrile-d$_3$, 298 K): δ (ppm) 7.38-7.42 (m, 12 H, H_{meta} and H_{para}), 7.34-7.30 (m, 8 H, H_{ortho}); **^{13}C NMR** (100 MHz, acetonitrile-d$_3$, 298 K): δ (ppm) 163.22 (m, $C_{carbonyl}$), 153.50 (m, C_{alkene}), 134.12 (m, C_{ortho}), 133.00 (m, C_{subst}), 129.84 (m, C_{para}), 128.73 (m, C_{meta}); **^{31}P{^{1}H} NMR** (162 MHz, acetonitrile-d$_3$, 298 K): δ (ppm) -18.37; **^{31}P{^{1}H} NMR** (162 MHz, DMF-d$_7$, 298 K): δ (ppm) -19.07; **^{31}P{^{1}H} NMR** (162 MHz, chloroform-d$_3$, 298 K): δ (ppm) -20.53; **HR-MS-ESI** m/z: $[M + H]^+$ 467.0954 (calculated for $C_{28}H_{21}O_3P_2$ 467.0960); **IR** (solid) λ_{max} (cm^{-1}) 3054 (w), 1834 (m), 1811 (m), 1757 (s), 1496 (w), 1484(w), 1435 (m), 1244 (s), 913 (s); **M.p.** 149.6 ˚C (lit.[1] 147 ˚C). Experimental values consistent with literature data.[1,2]

Bis(*p*-tolyl)phosphine: ^{1}H NMR (400 MHz, Chloroform-*d*) δ 7.48 – 7.36 (m, 4H, H_b), 7.22 – 7.12 (m, 4H, H_c), 5.25 (d, $J_{H\text{-}P}$ = 174.9 Hz, 1H, PH), 2.38 (s, 6H, H_e); **^{31}P{^{1}H} NMR** (162 MHz, Chloroform-*d*) δ -41.93; **^{31}P NMR** (162 MHz, Chloroform-*d*) δ_P -41.92 (d, *J* = 174.9 Hz). Experimental values were consistent with literature data.[3,4]

DP^{Tol}: ^{1}H NMR (500 MHz, Chloroform-*d*) δ_H 7.21 (dt, *J* = 8.6, 4.4 Hz, 8H, H_d), 7.08 (d, *J* = 7.7 Hz, 8H, H_e), 2.34 (s, 12H, H_g); **^{13}C{^{1}H} NMR** (125 MHz, Chloroform-*d*): δ_C (ppm) 162.84 (t, *J* = 2.86, C_a), 155.03 (m, C_b), 140.06 (s, C_f), 134.24 (m, C_d), 129.60 (t, *J* = 4.40, C_e), 129.04 (m, C_c), 21.55 (s, C_g); **^{31}P{^{1}H} NMR** (162 MHz, Chloroform-*d*) δ_P -23.08 (s); **HR-MS (ES):** m/z [M+H]+ 523.1602 (calculated for $C_{32}H_{28}O_3P_2$ 523.1592).

[Mo(CO)$_4$(DP^{Ph})]: ^{1}H NMR (500 MHz, dichloromethane-*d$_2$*) δ_H (ppm) 7.60 (m, 8H, H_d), 7.53 (m, 4H, H_f), 7.50 (m, 8H, H_e); **^{13}C{^{1}H} NMR** (125 MHz, dichloromethane-*d$_2$*): δ_C (ppm) 215.20 (m, CO_{eq}), 208.89 (t, J = 8.46 Hz, CO_{ax}), 166.24 (m, C_a), 159.01 (m, C_b), 133.05 (t, J = 8.67 Hz, C_d), 132.29 (m, C_c), 131.76 (s, C_f), 129.59 (t, J = 5.15 Hz, C_e); **^{31}P{^{1}H}** NMR (162 MHz, chloroform-*d*) δ_P (ppm) 51.68 (s); **^{31}P{^{1}H}** NMR (122 MHz, dichloromethane-*d$_2$*) δ_P (ppm) 49.91 (s).

[Mo(CO)$_4$(DP^{Tol})]: ^{1}H NMR (500 MHz, Chloroform-*d*) δ_H (ppm) 7.46 (m, 8H, H_d), 7.29 (dd, J = 8.13, J = 1.89, 8H, H_e), 2.41 (s, 12H, H_g); **^{13}C{^{1}H} NMR** (125 MHz, Chloroform-*d*): δ_C (ppm) 215.46 (m, CO_{eq}), 208.97 (t, J = 8.50, CO_{ax}), 165.82 (m, C_a), 159.11 (m, C_b), 142.25 (s, C_f), 132.84 (t, J = 6.96, C_d), 130.11 (t, J = 5.26, C_e), 128.85 (m, C_c), 21.56 (s, C_g); **^{31}P{^{1}H} NMR** (122 MHz, dichloromethane-*d$_2$*) δ_P (ppm) 48.37 (s).

(RNH$_3$)[Mo^0(CO)$_4$(DP^{Ph}-NHR)] (R = -CH$_2$CH$_2$OCH$_3$): ^{1}H NMR (500 MHz, dichloromethane-*d*) δ_H (ppm) 7.70 (m, 4H, $H_{d/d'}$), 7.62 (ddd, *J* = 11.1, 7.9, 1.7 Hz, 4H, $H_{d/d'}$), 7.35 (m, 12H, $H_{e/e'}$ and $H_{f/f'}$), 6.08 (t, *J* = 5.1 Hz, 1H, H_{amide}), 3.28 (t, *J* = 5.1 Hz, 2H, $H_{i'}$), 3.21 (s, 3H, $H_{j'}$), 3.07 (s, 3H, H_j), 2.98 (m, 4H, H_h and H_i), 2.64 (t, *J* = 5.1 Hz, 2H, $H_{h'}$); **^{13}C{^{1}H} NMR** (125 MHz, dichloromethane-*d*): δ_C (ppm) 217.86 (m, M-CO_{ax}), 210.03 (t, *J* = 8.6 Hz, M-CO_{eq}), 172.09 (d, *J* = 18.9 Hz, $C_{a/a'}$), 168.05 (d, *J* = 21.5 Hz, $C_{a/a'}$), 161.16 (dd, J_1 = 26.7, J_2 = 14.7 Hz, $C_{b/b'}$), 147.78 (dd, J_1 = 31.2, J_2 = 18.3 Hz, $C_{b/b'}$), 137.09 (dd, *J* = 36.3, 2.4 Hz, $C_{c/c'}$), 136.34 (dd, *J* = 35.9, 2.5 Hz, $C_{c/c'}$), 133.93 (d, *J* = 13.6 Hz, $C_{d/d'}$), 133.51 (d, *J* = 13.4 Hz, $C_{d/d'}$), 130.67 (d, *J* = 1.9 Hz, $C_{f/f'}$), 130.57 (d, *J* = 1.9 Hz, $C_{f/f'}$), 128.79 (d, *J* = 9.8 Hz, $C_{e/e'}$), 128.63 (d, *J* = 9.8 Hz, $C_{e/e'}$), 71.21 (s, C_i), 68.89 (s, $C_{i'}$), 59.34 (s, $C_{j'}$), 58.91 (s, C_j), 39.83 (s, C_h), 39.78 (s, $C_{h'}$); **^{31}P{^{1}H} NMR** (162 MHz, dichloromethane-*d*) δ_P (ppm) 72.54 (d, *J* = 3.3 Hz), 70.19 (d, *J* = 3.3 Hz); **HR-MS (ESI-):** m/z calculated for $C_{35}H_{29}NO_8P_2Mo$ $[M - H]^-$ = 750.0339; obs. = 750.0375; $[M\text{-}H\text{-}CO_2]^-$ = 706.0440; obs. = 706.0469; $\boldsymbol{\nu_{CO}}$ (cm^{-1}) 2023.60.

(RNH$_3$)[Mo^0(CO)$_4$(DP^{Tol}-NHR)] (R = -CH$_2$CH$_2$OCH$_3$): ^{1}H NMR (500 MHz, dichloromethane-*d$_2$*) δ 7.57 (dd, *J* = 10.9, 7.9 Hz, 4H, $H_{d/d'}$), 7.48 (dd, *J* = 10.9, 7.9 Hz, 4H, $H_{d/d'}$), 7.15 (ddd, *J* = 12.2, 8.1, 2.0 Hz, 8H, $H_{e/e'}$), 6.06 (t, *J* = 4.8 Hz, 1H, H_{amide}), 3.30 (t, *J* = 5.0 Hz, 2H, $H_{i'}$), 3.22 (s, 3H, $H_{j'}$), 3.07 (s, 3H, H_j), 2.98 (m, 4H, H_h and H_i), 2.69 (t, *J* = 5.1 Hz, 2H, $H_{h'}$), 2.35 (s, 6H, $H_{g/g'}$), 2.32 (s, 7H, $H_{g/g'}$); **^{13}C NMR** (126 MHz, dichloromethane-*d$_2$*) δ 217.85 (m, *J* = 33.4, 26.1, 8.1 Hz, CO_{eq}), 209.82 (t, *J* = 8.7 Hz, CO_{ax}),

171.91 (d, J = 18.9 Hz, $C_{a/a'}$), 167.91 (d, J = 21.4 Hz, $C_{a/a'}$), 161.02 (dd, J_1 = 26.9, J_2 = 14.6 Hz, $C_{b/b'}$), 147.44 (dd, J_1 = 31.5, J_2 = 18.4 Hz, $C_{b/b'}$), 140.53 (d, J = 1.8 Hz, $C_{f/f'}$), 140.39 (d, J = 1.8 Hz, $C_{f/f'}$), 133.53 (dd, J_1 = 37.8, J_2 = 2.2 Hz, $C_{c/c'}$), 133.51 (d, J = 13.9 Hz, $C_{d/d'}$), 133.07 (d, J = 13.6 Hz, $C_{d/d'}$), 132.71 (dd, J_1 = 37.4, J_2 = 2.3 Hz, $C_{c/c'}$), 129.10 (d, J = 10.1 Hz, $C_{e/e'}$), 128.92 (d, J = 10.2 Hz, $C_{e/e'}$), 70.79 (s, C_i), 68.56 (s, $C_{i'}$), 58.91 (s, $C_{j'}$), 58.52 (s, C_j), 39.48 (s, C_h), 39.39 (s, $C_{h'}$), 21.46 (s, $C_{g/g'}$); **$^{31}P\{^1H\}$ NMR** (162 MHz, dichloromethane-d_2) δ 70.78 (d, J = 2.4 Hz), 68.49 (d, J = 2.4 Hz); **HR-MS (ESI-):** m/z $[M-H-CO_2]^-$ 762.1070 (calculated for $C_{39}H_{37}NO_8P_2Mo^-$ 762.1066).

DP^{Tol}-RGD: ^1H NMR (700 MHz, DMF-d_7 with DIPEA, 298 K): δ (ppm) 1.205 (m, 1H, Lys, H_{γ}), 1.307 (m, 1H, Lys, H_{γ}), 1.314 (m, 2H, Lys, H_{δ}), 1.554 (m, 2H, Arg, H_{γ}), 1.593 (m, 1H, Lys, H_{β}), 1.669 (m, 1H, Lys, H_{β}), 1.758 (m, 1H, Arg, H_{β}), 1.833 (m, 1H, Arg, H_{β}), 2.258 (s, 3H, DP^{Tol}, $H_{g/g'}$), 2.267 (s, 3H, DP^{Tol} , $H_{g/g'}$), 2.281 (s, 6H, DP^{Tol}, $H_{g/g'}$), 2.427 (m, 1H, Asp, H_{β}), 2.678 (dd, J_1 = 13.52 Hz, J_2 = 8.88 Hz, 1H, Phe, H_{β}), 2.797 (m, 2H, Lys, H_{ε}), 2.845 (m, 1H, Asp, H_{β}), 3.102 (m, 1H, Arg, H_{δ}), 3.141 (m, 1H, Arg, H_{δ}), 3.304 (m, 1H, Phe, H_{β}), 3.400 (dd, J_1 = 16.26 Hz, J_2 = 2.87 Hz, 1H, Gly, H_{α}), 4.291 (dd, J_1 = 16.23 Hz, J_2 = 8.91 Hz, 1H, Gly, H_{α}), 4.328 (dd, J_1 = 14.96 Hz, J_2 = 8.27 Hz, 1H, Lys, H_{α}), 4.409 (s br, 1H, Arg, H_{α}), 4.467 (s br, 1H, Asp, H_{α}), 4.813 (td, J_1 = 8.83 Hz, J_2 = 5.81 Hz, 1H, Phe, H_{α}), 6.999-7.039 (m, 8H, DP^{Ph} $H_{e/e'}$), 7.228-7.263 (m, 4H, Phe, H_o and H_m), 7.197 (m, 1H, Lys, NH_{ζ}), 7.162 (tt, J_1 = 6.60 Hz, J_2 = 2.06 Hz, 1H, Phe, H_p), 7.303 (dt, J_1 = 19.15 Hz, J_2 = 7.51 Hz, 4H, DP^{Tol} $H_{d/d'}$), 7.466 (t, J = 7.58 Hz, 4H, DP^{Tol} $H_{d/d'}$), 7.900 (m, 1H, Phe, NH), 8.068 (d, J = 7.76 Hz, 1H, Gly, NH), 8.236 (m, 1H, Asp, NH), 8.244 (m, 1H, Lys, NH), 8.646 (s br, 1H, Arg, NH); **^{13}C NMR** (176 MHz, DMF-d_7 with DIPEA, 298 K): δ (ppm) 20.644 (s, DP^{Tol}, $C_{g/g'}$), 20.657 (s, DP^{Tol}, $C_{g/g'}$), 20.681 (s, DP^{Tol}, $C_{g/g'}$), 23.430 (s, Lys, C_{γ}), 25.260 (s, Arg, C_{γ}), 27.268 (s, Arg, C_{β}), 28.889 (s, Lys, C_{δ}), 32.873 (m weak, Lys, C_{β}), 36.898 (s, Phe, C_{β}), 38.034 (hidden, Asp, C_{β}), 38.513 (s, Lys, C_{ε}), 40.946 (s, Arg, C_{δ}), 43.180 (s, Gly, C_{α}), 49.411 (s, Asp, C_{α}), 51.878 (s, Arg, C_{α}), 53.675 (s, Phe, C_{α}), 55.472 (s, Lys, C_{α}), 126.134 (s, Phe, C_p), 128.143 (s, Phe, $C_{o/m}$), 128.40 (dd, J_1 = 6.9, J_2 = 2.2 Hz, DP^{Tol}, $C_{e/e'}$), 128.47 (d, J = 7.4 Hz, DP^{Tol}, $C_{e/e'}$), 129.43 (s, Phe, $C_{o/m}$), 133.85 (t, J = 19.7 Hz, DP^{Tol}, $C_{d/d'}$), 134.56 (d, J = 20.7 Hz, DP^{Tol}, $C_{d/d'}$), 137.467 (d, J = 6.7 Hz, DP^{Tol}, $C_{c/c'}$), 137.863 (s, DP^{Tol}, $C_{f/f'}$), 138.878 (s, Phe, C_s), quaternary carbons: 158.380 (s), 158.787 (s), 158.833 (s), 159.009 (s), 171.169 (s), 171.471 (s), 171.950 (s), 172.015 (s), 172.929 (s), remaining signals corresponding to quaternary carbons could not be distinguished from noise; **$^{31}P\{^1H\}$ NMR** (283 MHz, DMF-d_7 with DIPEA, 298 K): δ (ppm) -15.156 (d, J = 161.00 Hz), -13.837 (d, J = 161.00 Hz); **HRMS-ESI** m/z: $[M + H]^+$ 1126.4713 (calculated for $C_{59}H_{70}O_{10}N_9P_2$ 1126.4715), $[M + MeOH + H]^+$ 1158.4520 (calculated for $C_{60}H_{74}O_{11}N_9P_2$ 1158.4980).

DP^{Ph}-PSMAt: ^1H NMR (700 MHz, DMF-d_7 with DIPEA, 298 K): δ (ppm) 1.382-1.436 (m, 2H, Lys, H_{γ}), 1.445-1.504 (m, 2H, Lys, H_{δ}), 1.608-1.660 (m, 1H, Lys, H_{β}), 1.742-1.795 (m, 1H, Lys, H_{β}), 1.842-1.893 (m, 1H, Glu, H_{β}), 1.986-2.039 (m, 1H, Glu, H_{β}), 2.324-2.364 (m, 1H, Glu, H_{γ}), 2.386 (t, J = 6.24 Hz, 2H, PEG, H_o), 2.455 (dt, J_1 = 14.68 Hz, J_2 = 8.39 Hz, 1H, Glu, H_{γ}), 2.955-2.983 (m, 2H, PEG, H_h), 3.014-3.045 (m, 2H, PEG, H_i), 3.128 (dd, J_1 = 12.81 Hz, J_2 = 6.46 Hz, 2H, Lys, H_{ε}), 3.417-3.431 (m, 2H, PEG, H_{j-o}), 3.520-3.591 (m, 10H, PEG, H_{j-o}), 3.683 (t, J = 6.24 Hz, 2H, PEG, H_p), 4.204-4.233 (m, 1H, Lys, H_{α}), 4.261-4.285 (m, 1H, Glu, H_{α}), 6.543 (m, 1H, Glu, NH), 6.639 (d, J = 7.48 Hz, 1H, Lys, NH), 7.208-7.257 (m, 12H, DP^{Ph}, $H_{e/f}$), 7.431-7.466 (m, 4H, DP^{Ph}, $CH_{d/d'}$), 7.574-7.601 (m, 4H, DP^{Ph}, $H_{d/d'}$), 7.806 (t, J = 5.44 Hz, 1H, PEG, NH), 7.828 (t, J = 5.67 Hz, 1H, Lys, NH_{ζ}); **^{13}C NMR** (176 MHz, DMF-d_7 with DIPEA, 298 K): δ (ppm), 23.063 (s, Lys, C_{γ}), 29.320 (Lys, C_{δ}), 29.840 (Glu, C_{β}), 32.208 (s, Glu, C_{γ}), 32.673 (s, Lys, C_{β}), 36.718 (s, PEG, C_q), 38.739 (s, PEG, C_h), 38.834 (s, Lys, C_{ε}), 53.364 (s, Glu, C_{α}), 53.498 (s, Lys, C_{α}), 67.398 (s, PEG, C_p), 69.051 (s, PEG, C_i), 70.096 (s, PEG, C_{j-o}), 70.217 (s, PEG, C_{j-o}), 70.292 (s, PEG, C_{j-o}), 70.419 (s, PEG, C_{j-o}), 70.430 (s, PEG, C_{j-o}), 127.814 (d, J = 6.78 Hz, DP^{Ph}, $C_{e/e'}$), 127.870 (d, J = 7.35 Hz, DP^{Ph}, $C_{e/e'}$), 128.150 (s, DP^{Ph}, $C_{f/f'}$), 128.379 (s, DP^{Ph}, $C_{f/f'}$), 134.035 (dd, J_1 = 19.47 Hz, J_2 = 5.82 Hz, DP^{Ph}, $C_{d/d'}$), 134.653 (d, J = 20.35 Hz, DP^{Ph}, $C_{d/d'}$), 136.936 (m, DP^{Ph}, $C_{c/c'}$), 137.650 (m, DP^{Ph}, $C_{c/c'}$), quaternary carbons: 157.065 (s), 170.452 (s), 174.729 (s), 175.006 (s), 175.232 (s), remaining signals corresponding to quaternary carbons could not be distinguished from noise; **$^{31}P\{^1H\}$ NMR** (283 MHz,

DMF-d_7 with DIPEA, 298 K): δ (ppm) -13.18 (d, J = 162.7 Hz), -12.15 (d, J = 162.7 Hz); **HR-MS-ESI** m/z: $[M + H]^+$ 1033.3759 (calculated for $C_{51}H_{63}O_{15}N_4P_2$ 1033.3760), $[M + Na]^+$ 1055.3579 (calculated for $C_{51}H_{62}O_{15}N_4P_2Na$ 1055.3579).

DP^{Tol}-PSMAt: ^{1}H NMR (700 MHz, DMF-d_7 with DIPEA, 298 K): δ (ppm) 1.389-1.444 (m, 2H, Lys, H_γ), 1.453-1.503 (m, 2H, Lys, H_δ), 1.618-1.670 (m, 1H, Lys, H_β), 1.752-1.801 (m, 1H, Lys, H_β), 1.899-1.949 (m, 1H, Glu, H_β), 1.983-2.035 (m, 1H, Glu, H_β), 2.262 (s, 6H, DP^{Tol}, $H_{g/g'}$), 2.293 (s, 6H, DP^{Tol}, $H_{g/g'}$), 2.345-2.384 (m, 1H, Glu, H_γ), 2.389 (t, J = 6.25 Hz, 2H, PEG, H_o), 2.451 (dt, J_1 = 15.00 Hz, J_2 = 8.17 Hz, 1H, Glu, H_γ), 2.962-3.008 (m, 4H, PEG, $H_{h/i}$), 3.139 (hidden, 2H, Lys, H_ε), 3.407-3.421 (m, 2H, PEG, H_{j-o}), 3.524-3.591 (m, 10H, PEG, H_{j-o}), 3.685 (t, J = 6.25 Hz, 2H, PEG, H_p), 4.217-4.247 (m, 1H, Lys, H_α), 4.268-4.294 (m, 1H, Glu, H_α), 6.559 (d, J = 4.84 Hz, 1H, Glu, NH), 6.631 (d, J = 7.72 Hz, 1H, Lys, NH), 7.005 (d, J = 7.63 Hz, 4H, DP^{Ph}, H_e), 7.036 (d, J = 7.62 Hz, 4H, DP^{Ph}, H_e), 7.325 (dd, J_1 = 14.22 Hz, J_2 = 7.36 Hz, 4H, DP^{Ph}, $CH_{d/d'}$), 7.427 (t, J = 7.68 Hz, 4H, DP^{Ph}, $H_{d/d'}$), 7.799-7.824 (m, 1H, PEG, NH), 7.799-7.824 (m, 1H, Lys, NH_ζ); **^{13}C NMR** (176 MHz, DMF-d_7 with DIPEA, 298 K): δ (ppm) 20.667 (s, DP^{Tol}, $C_{g/g'}$), 20.686 (s, DP^{Tol}, $C_{g/g'}$), 23.064 (s, Lys, C_γ), 29.306 (hidden, Lys, C_δ), 29.680 (hidden, Glu, C_β), 31.929 (s, Glu, C_γ), 32.656 (s, Lys, C_β), 36.726 (s, PEG, C_q), 38.703 (s, PEG, C_h), 38.840 (s, Lys, C_ε), 53.328 (s, Glu, C_α), 53.453 (s, Lys, C_α), 67.389 (s, PEG, C_p), 69.058 (s, PEG, C_i), 70.176 (s, PEG, C_{j-o}), 70.223 (s, PEG, C_{j-o}), 70.308 (s, PEG, C_{j-o}), 70.436 (s, PEG, C_{j-o}), 70.454 (s, PEG, C_{j-o}), 128.505 (d, J = 7.07 Hz, DP^{Tol}, $C_{e/e'}$), 128.568 (d, J = 7.35 Hz, DP^{Tol}, $C_{e/e'}$), 134.197 (d, J = 19.96 Hz, DP^{Tol}, $C_{d/d'}$), 134.675 (d, J = 20.96 Hz, DP^{Tol}, $C_{d/d'}$), 137.682 (s, DP^{Tol}, $C_{c/c'}$), 137.949 (s, DP^{Tol}, $C_{f/f'}$), quaternary carbons: 158.097 (s), 170.435 (s), 174.665 (s), 175.003 (s), 175.179 (s), remaining signals corresponding to quaternary carbons could not be distinguished from noise; **^{31}P{^{1}H} NMR** (283 MHz, DMF-d_7 with DIPEA, 298 K): δ (ppm) -15.776 (d, J = 151.40 Hz), -14.392 (d, J = 151.40 Hz); **HR-MS-ESI** m/z: $[M + H]^+$ 1089.4373 (calculated for $C_{55}H_{71}O_{15}N_4P_2$ 1089.4386), $[M + Na]^+$ 1111.4193 (calculated for $C_{55}H_{70}O_{15}N_4P_2Na$ 1111.4205), $[M + MeOH + H]^+$ 1121.4275 (calculated for $C_{56}H_{75}O_{16}N_4P_2$ 1121.4648).

[^{nat}ReO$_2$(DP^{Ph}-PSMAt)$_2$]$^+$: ^{1}H NMR (700 MHz, ammonium acetate-d$_7$ in D_2O, pH 4.5-5, 298 K): δ (ppm) 1.26-1.31 (m, 4H, Lys, H_γ), 1.39-1.45 (m, 4H, Lys, H_δ), 1.54-1.59 (m, 2H, Lys, H_β), 1.65-1.71 (m, 2H, Lys, H_β), 1.79-1.84 (m, 2H, Glu, H_β), 1.98-2.03 (m, 2H, Glu, H_β), 2.29-2.34 (m, 4H, Glu, H_γ), 2.41 (t, J = 6.09 Hz, 4H, PEG, H_q), 2.98-2.99 (m, 2H, PEG, H_i), 3.01-3.03 (m, 2H, PEG, H_h), 3.06 (br s, 4H, PEG, H_{h-i}), 3.09 (t, J = 6.89 Hz, 4H, Lys, H_ε), 3.29-3.33 (m, 4H, PEG, H_j), 3.40-3.42 (m, 4H, PEG, H_k), 3.50-3.57 (m, 16H, PEG, H_{l-o}), 3.68 (t, J = 5.97 Hz, 4H, PEG, H_p), 3.98 (dd, J_1 = 8.39 Hz, J_2 = 5.09 Hz, 2H, Lys, H_α), 4.02 (dd, J_1 = 8.48 Hz, J_2 = 4.93 Hz, 2H, Glu, H_α), 6.56 (m, urea-NH), 6.65 (m, urea-NH), 6.90-7.03 (m, 12 H, DP^{Ph}, $H_{d/d'}$), 7.13-7.18 (m, 12 H, DP^{Ph}, $H_{e/e'}$), 7.42-7.47 (m, 7 H, DP^{Ph}, $H_{f/f'}$); **^{13}C NMR** (176 MHz, ammonium acetate-d$_7$ in D_2O, 298 K): δ (ppm) 22.41 (s, Lys, C_γ), 27.88 (s, Glu, C_β), 27.96 (s, Lys, C_δ), 31.31 (s, Glu, C_γ), 31.54 (s, Lys, C_β), 38.94 (s, PEG, C_h), 39.01 (s, PEG, C_h), 36.08 (s, PEG, C_q), 39.14 (s, Lys, C_ε), 54.33 (s, Glu, C_α), 54.68 (s, Lys, C_α), 66.84 (s, PEG, C_p), 68.16 (s, PEG, C_i), 68.23 (s, PEG, C_i), 69.21 (s, PEG, C_j), 69.48-69.56 (overlapping signals, PEG, C_{k-o}), 125.92-126.49 (m, DP^{Ph}, $C_{c/c'}$), 128.50 (br d, J = 32.6 Hz, DP^{Ph}, $C_{e/e'}$), 132.32 (d, J = 18.4 Hz, DP^{Ph}, $C_{f/f'}$), 133.86 (br s, DP^{Ph}, $C_{d/d'}$), 134.11 (br s, DP^{Ph}, $C_{d/d'}$), 145.79-146.47 (m, DP^{Ph}, $C_{b/b'}$), 158.43-158.72 (m, DP^{Ph}, $C_{b/b'}$), 159.14 (s, C_{urea}), 165.95-166.05 (m, DP^{Ph}, $C_{a/a'}$), 168.19-168.26 (m, DP^{Ph}, $C_{a/a'}$), 173.75 (s, C_r), 178.66 (br s, $C_{carbonyl}$), 178.93 (s, $C_{carbonyl}$), 179.43 (s, $C_{carbonyl}$); **^{31}P{^{1}H} NMR** (283 MHz, ammonium acetate-d$_7$ in D_2O, 298 K): δ (ppm) 22.50 (sharp m, *trans*-Re-DP^{Ph}-PSMAt), 24.49 (dm, J_1 = 357.43 Hz, *cis*-Re-DP^{Ph}-PSMAt), 25.37 (dm, J_1 = 357.43 Hz, *cis*-Re-DP^{Ph}-PSMAt), 27.99 (sharp m, *trans*-Re-DP^{Ph}-PSMAt); **Simulated ^{31}P{^{1}H} NMR** (***cis*-[^{nat}ReO$_2$(DP^{Ph}-PSMAt)$_2$]$^+$**, 283 MHz, ammonium acetate-d$_7$ in D_2O, pH 4.5-5, 298 K): δ (ppm) 24.4 (m, $J_{AB'} = J_{A'B}$ = 360 Hz, $J_{AB} = J_{A'B'}$ = 15.0 Hz, $J_{BB'}$ = 0.25 Hz), 26.2 (m, $J_{AB'} = J_{A'B}$ = 360 Hz, $J_{AB} = J_{A'B'}$ = 15.0 Hz, $J_{AA'}$ = 0.25 Hz); **Simulated ^{31}P{^{1}H} NMR** (***trans*-[^{nat}ReO$_2$(DP^{Ph}-PSMAt)$_2$]$^+$**, 283 MHz, ammonium acetate-d$_7$ in D_2O, pH 4.5-5, 298 K): δ (ppm) 22.5 (m, $J_{BB'}$ = 360 Hz, $J_{AB} = J_{A'B'}$ = 15.0 Hz, $J_{AB'} = J_{A'B}$ = 0.25 Hz), 28.0 (m, $J_{AA'}$ = 360 Hz, $J_{AB} = J_{A'B'}$ = 15.0 Hz, $J_{AB'} = J_{A'B}$ = 0.25 Hz); **HR-MS-ESI** m/z: $[M + H]^{2+}$ 1142.3402 (calculated for $C_{102}H_{125}O_{32}N_8P_4Re$ 1142.3454).

[^{nat}ReO$_2$(DP^{Tol}-PSMAt)$_2$]$^+$: ^{1}H NMR (700 MHz, ammonium acetate-d$_7$ in D$_2$O, pH 4.5-5, 298 K): δ (ppm) 1.26-1.30 (m, 4H, Lys, H$_\gamma$), 1.39-1.44 (m, 4H, Lys, H$_\delta$), 1.53-1.59 (m, 2H, Lys, H$_\beta$), 1.65-1.70 (m, 2H, Lys, H$_\beta$), 1.78-1.84 (m, 2H, Glu, H$_\beta$), 1.97-2.02 (m, 2H, Glu, H$_\beta$), 2.29 (br s, 12H, DP^{Tol}, H$_{g/g'}$), 2.31 (br s, 12H, DP^{Tol}, H$_{g/g'}$), 2.31 (hidden, 2H, Glu, H$_\gamma$), 2.39-2.41 (m, 4H, PEG, H$_q$), 2.98-3.00 (m, 2H, PEG, H$_i$), 3.02-3.06 (m, 6H, PEG, H$_{h\text{-}i}$), 3.107-3.10 (m, 4H, Lys, H$_\varepsilon$), 3.27-3.31 (m, 4H, PEG, H$_j$), 3.39-3.41 (m, 4H, PEG, H$_k$), 3.50-3.56 (overlapping m, 16 H, PEG, H$_{l\text{-}o}$), 3.66-3.68 (m, 4H, PEG, H$_p$), 3.95-3.97 (m, 2H, Lys, H$_\alpha$), 4.00-4.02 (m, 2H, Glu, H$_\alpha$), 6.79-6.89 (4 overlapping m, DP^{Tol}, > 13H, H$_{d/d'}$), 6.95 (br d, *J* = 7.89 Hz, > 6H, DP^{Tol}, H$_{e/e'}$), 6.97 (br d, *J* = 7.57 Hz, > 6H, DP^{Tol}, H$_{e/e'}$); **^{13}C NMR** (176 MHz, ammonium acetate-d$_7$ in D$_2$O, 298 K): *δ* (ppm) 20.54 (s, DP^{Tol}, C$_{g/g'}$), 20.58 (s, DP^{Tol}, C$_{g/g'}$), 22.42 (s, Lys, C$_\gamma$), 27.98 (s, Glu, C$_\beta$), 28.03 (s, Lys, C$_\delta$), 31.50 (s, Glu, C$_\gamma$), 31.62 (s, Lys, C$_\beta$), 36.08 (s, PEG, C$_q$), 38.91 (s, PEG, C$_h$), 38.97 (s, PEG, C$_h$), 39.14 (s, Lys, C$_\varepsilon$), 54.45 (s, Glu, C$_\alpha$), 54.80 (s, Lys, C$_\alpha$), 66.85 (s, PEG, C$_p$), 68.21 (s, PEG, C$_i$), 68.27 (s, PEG, C$_i$), 69.24 (s, PEG, C$_{j\text{-}k}$), 69.44 (s, PEG, C$_{j\text{-}k}$), 69.49-69.56 (overlapping signals, PEG, C$_{h\text{-}o}$), 122.80-123.30 (m, DP^{Tol}, C$_{c/c'}$), 128.94 (br d, *J* = 39.0 Hz, DP^{Tol}, C$_{e/e'}$), 133.85 (br s, D DP^{Tol}, C$_{d/d'}$), 134.09 (br s, DP^{Tol}, C$_{d/d'}$), 143.18 (d, *J* = 19.8 Hz, DP^{Tol}, C$_{f/f'}$), 145.88-146.40 (m, DP^{Tol}, C$_{b/b'}$), 158.53-158.77 (m, DP^{Tol}, C$_{b/b'}$), 159.13 (s, C$_{urea}$), 166.24-166.34 (m, DP^{Tol}, C$_{a/a'}$), 168.63-168.69 (m, DP^{Tol}, C$_{a/a'}$), 173.73 (s, C$_r$), 178.82 (s, C$_{carbonyl}$), 179.05 (s, C$_{carbonyl}$), 179.18 (s, C$_{carbonyl}$), 179.59 (s, C$_{carbonyl}$); **^{31}P{^{1}H} NMR** (283 MHz, ammonium acetate-d$_7$ in D$_2$O, 298 K): δ (ppm) 22.12 (sharp m, *trans*-Re-DP^{Tol}-PSMAt), 23.82 (dm, *J*$_1$ = 359.95 Hz, *cis*-Re-DP^{Tol}-PSMAt), 25.35 (dm, *J*$_1$ = 359.95 Hz, *cis*-Re-DP^{Tol}-PSMAt), 26.91 (sharp m, *trans*-Re-DP^{Tol}-PSMAt); **Simulated ^{31}P{^{1}H} NMR** (***cis*-[^{nat}ReO$_2$(DP^{Tol}-PSMAt)$_2$]$^+$**, 283 MHz, ammonium acetate-d$_7$ in D$_2$O, pH 4.5-5, 298 K): δ (ppm) 23.8 (m, $J_{AB'} = J_{A'B}$ = 360 Hz, $J_{AB} = J_{A'B'}$ = 15.0 Hz, $J_{BB'}$ = 0.25 Hz), 25.35 (m, $J_{AB'} = J_{A'B}$ = 360 Hz, $J_{AB} = J_{A'B'}$ = 15.0 Hz, $J_{AA'}$ = 0.25 Hz); **Simulated ^{31}P{^{1}H} NMR** (***trans*-[^{nat}ReO$_2$(DP^{Tol}-PSMAt)$_2$]$^+$**, 283 MHz, ammonium acetate-d$_7$ in D$_2$O, pH 4.5-5, 298 K): δ (ppm) 22.12 (m, $J_{BB'}$ = 360 Hz, $J_{AB} = J_{A'B'}$ = 15.0 Hz, $J_{AB'} = J_{A'B}$ = 0.25 Hz), 26.91 (m, $J_{AA'}$ = 360 Hz, $J_{AB} = J_{A'B'}$ = 15.0 Hz, $J_{AB'} = J_{A'B}$ = 0.25 Hz); **HR-MS-ESI** m/z: [M + H]$^{2+}$ 1198.4039 (calculated for $C_{110}H_{141}O_{32}N_8P_4Re$ 1198.4080).

[Cu(DP^{Ph}-PSMAt)$_2$]$^+$: ^{1}H NMR (700 MHz, DMF-*d*$_7$, 298 K): δ (ppm) 1.410 (m, 4H, Lys, H$_\gamma$), 1.477 (m, ≥ 3H, Lys, H$_\delta$), 1.642 (m, partially hidden, 2H, Lys, H$_\beta$), 1.770 (m, 2H, Lys, H$_\beta$), 1.891 (m, 2H, Glu, H$_\beta$), 2.113 (m, 1H, Glu, H$_\beta$), 2.324-2.364 (m, 1H, Glu, H$_\gamma$), 2.407 (t, *J* = 6.24 Hz, 4H, PEG, H$_o$), 2.429 (m, 4H, Glu, H$_\gamma$), 3.135 (m, 4H, Lys, H$_\varepsilon$), 3.349 (m, ≥ 17H, PEG, H$_{h\text{-}o}$), 3.694 (t, *J* = 6.35 Hz, 2H, PEG, H$_p$), 4.235 (m, 1H, Lys, H$_\alpha$), 4.277 (m, 1H, Lys, H$_\alpha$), 4.362(m, 2H, Glu, H$_\alpha$), 6.610 (m, ≥ 2H, Lys and Glu, NH), 7.065-7.380 (3x overlapping br m, ≥ 29H, H$_{d/d'}$, H$_{e/e'}$ and H$_{f/f'}$), 7.845 (m, 2H, Lys, NH$_\zeta$); **^{31}P{^{1}H} NMR** (283 MHz, DMF-*d*$_7$, 298 K): δ (ppm) 11.94 (br s; with shoulder towards higher ppm); **HR-MS-ESI** m/z: [M + H]$^{2+}$ 1065.3402 (calculated for $C_{102}H_{125}O_{30}N_8P_4Cu$ 1065.3385).

[Cu(DP^{Tol}-PSMAt)$_2$]$^+$: ^{1}H NMR (700 MHz, DMF-*d*$_7$, 298 K): δ (ppm) 1.412 (m, 4H, Lys, H$_\gamma$), 1.479 (m, 4H, Lys, H$_\delta$), 1.641 (m, 2H, Lys, H$_\beta$), 1.776 (m, 2H, Lys, H$_\beta$), 2. 895 (m, 2H, Glu, H$_\beta$), 2.115 (m, 2H, Glu, H$_\beta$), 2.296 (br s, ≥ 10H, DP^{Tol}, H$_{g/g'}$), 2.331 (br s, 6H, DP^{Tol}, H$_{g/g'}$), 2.335 (br s, 6H, DP^{Tol}, H$_{g/g'}$), 2.407 (t, *J* = 6.28 Hz, 4H, PEG, H$_o$), 2.426 (m, 4H, Glu, H$_\gamma$), 3.137 (m, 4H, PEG, H$_{h/i}$), 3.271 (m, 2H, Lys, H$_\varepsilon$), 3.552 (br m, ≥ 14H, PEG, H$_{j\text{-}o}$), 3.693 (t, *J* = 6.38 Hz, 4H, PEG, H$_p$), 4.243 (m, 1H, Lys, H$_\alpha$), 4.280 (m, 1H, Lys, H$_\alpha$), 4.364 (m, 2H, Glu, H$_\alpha$), 6.608 (m, 4H, Lys and Glu, NH), 6.893-7.255 (3x overlapping br m, ≥ 20H, DP^{Tol}, H$_{d/d'}$ and H$_{e/e'}$), 7.830 (s, 1H, Lys, NH$_\zeta$), 7.868 (m, 1H, Lys, NH$_\zeta$); **^{31}P{^{1}H} NMR** (283 MHz, DMF-d$_7$, 298 K): δ (ppm) 10.81 (br s; with shoulder towards higher ppm); **HR-MS-ESI** m/z: [M + H]$^{2+}$ 1121.3953 (calculated for $C_{110}H_{141}O_{30}N_8P_4Cu$ 1121.4012).

3. NMR Spectra:

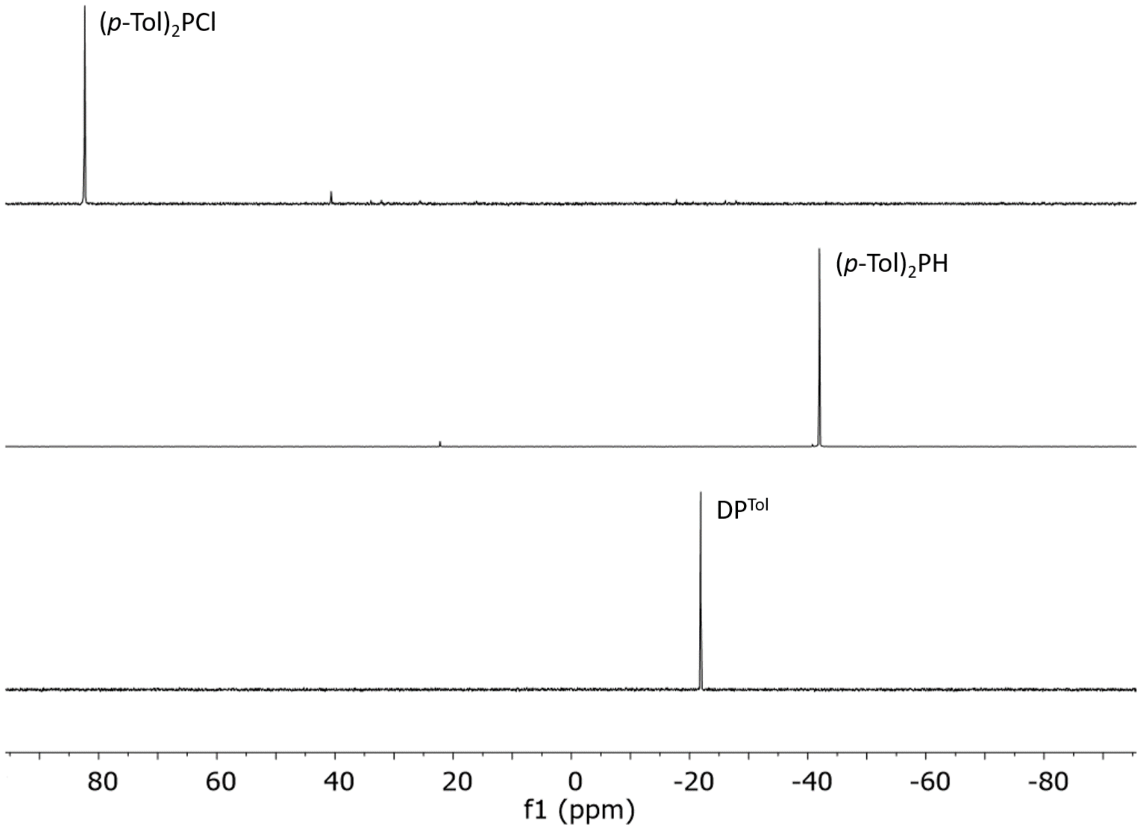

*Figure S1: $^{31}P\{^{1}H\}$ NMR spectra of DP^{Tol} (-23.1 ppm, **bottom**) and the precursors di-p-tolylphosphine (-41.9 ppm, **middle**) and bis(p-tolyl)chlorophosphine (82.4 ppm, **top**) in chloroform-d.*

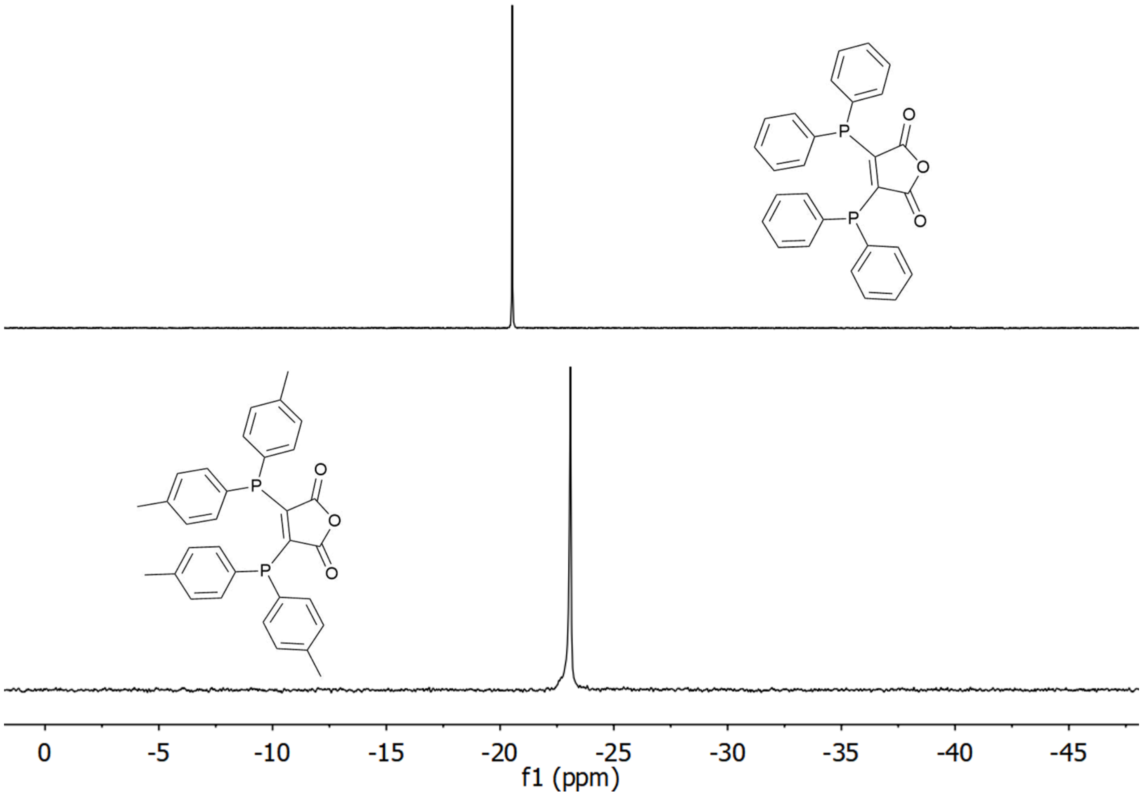

Figure S2: Comparison of the $^{31}P\{^{1}H\}$ NMR signals of DP^{Ph} and DP^{Tol} in chloroform-d.

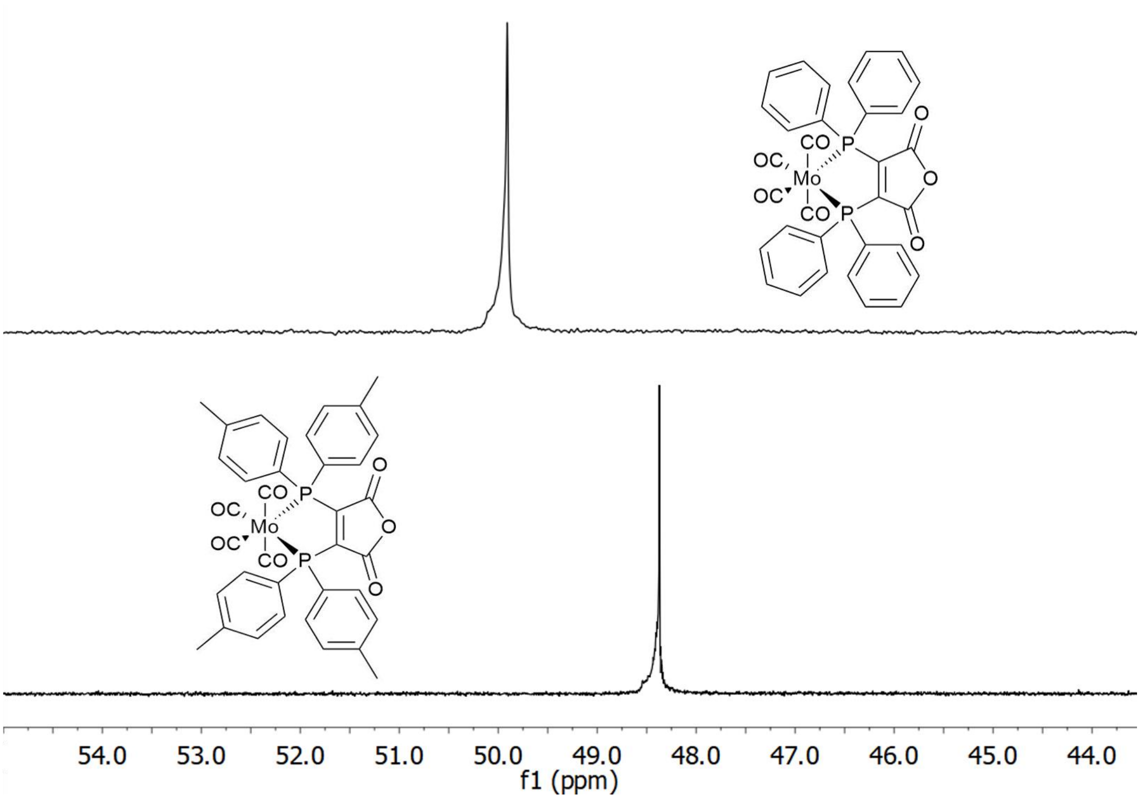

*Figure S3: $^{31}P\{^{1}H\}$ NMR signals for $[Mo(CO)_4(DP^{Ph})]$ (**top**) and $[Mo(CO)_4(DP^{Tol})]$ (**bottom**) in dichloromethane-d_2.*

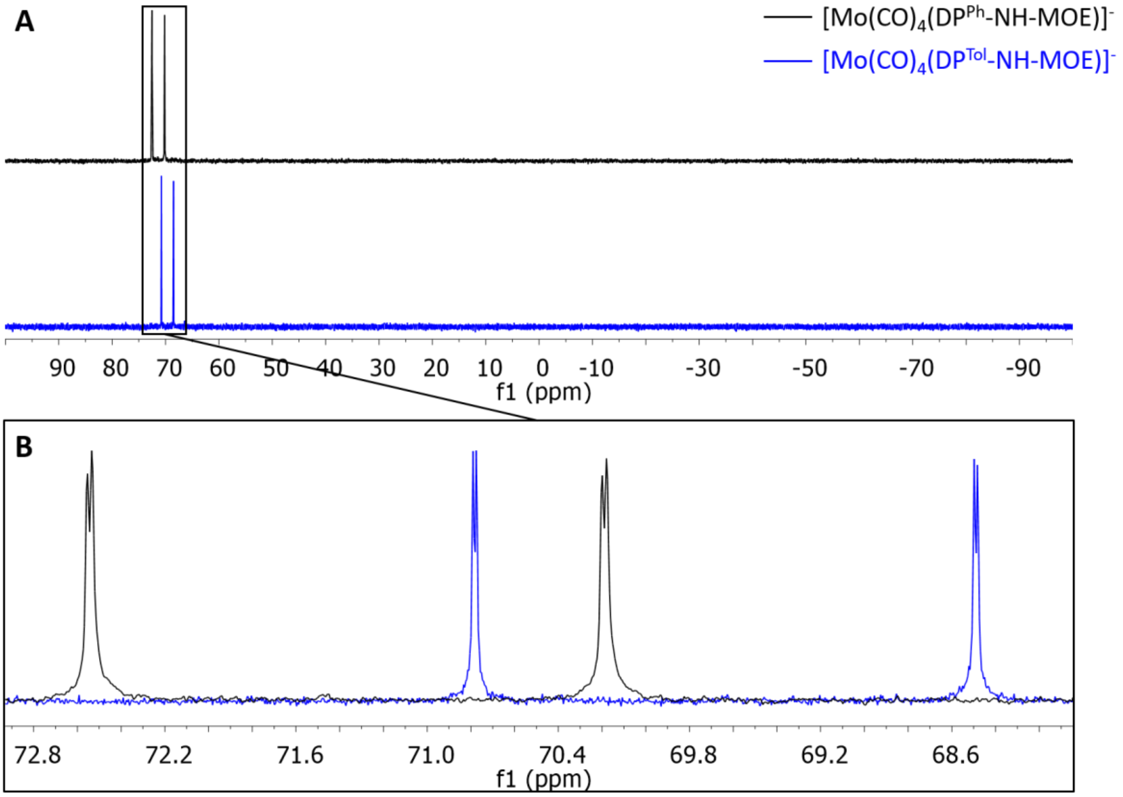

*Figure S4: Full $^{31}P\{^{1}H\}$ NMR spectra (**top, A**) and peak magnification with superimposed spectra (**bottom, B**) for $[Mo^0(CO)_4(DP^{Ph}\text{-}NHR)]^-$ (black, two doublets at 72.54 ppm and at 70.19 ppm, $J_{P\text{-}P}$ = 3.3 Hz) and $[Mo^0(CO)_4(DP^{Tol}\text{-}NHR)]^-$ (blue, two doublets at δ 70.78 ppm and at 68.49 ppm, $J_{P\text{-}P}$ = 2.4 Hz; R = $-CH_2CH_2OCH_3$) in dichloromethane-d_2.*

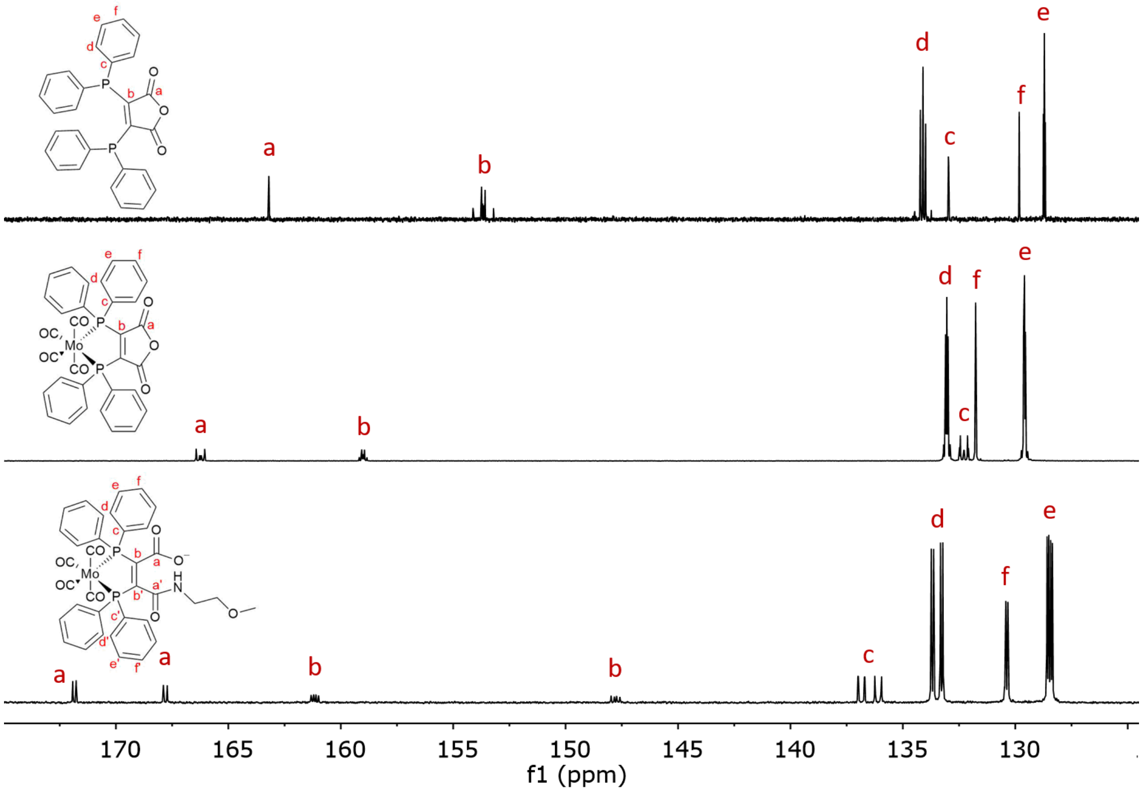

Figure S5: ^{13}C NMR signals for DP^{Ph} in chloroform-d, and of $(RNH_3)[Mo^0(CO)_4(DP^{Ph})]$ and $(RNH_3)[Mo^0(CO)_4(DP^{Ph}$-$NHR)]$ (R = $-CH_2CH_2OCH_3$/MOE) in dichloromethane-d_2.

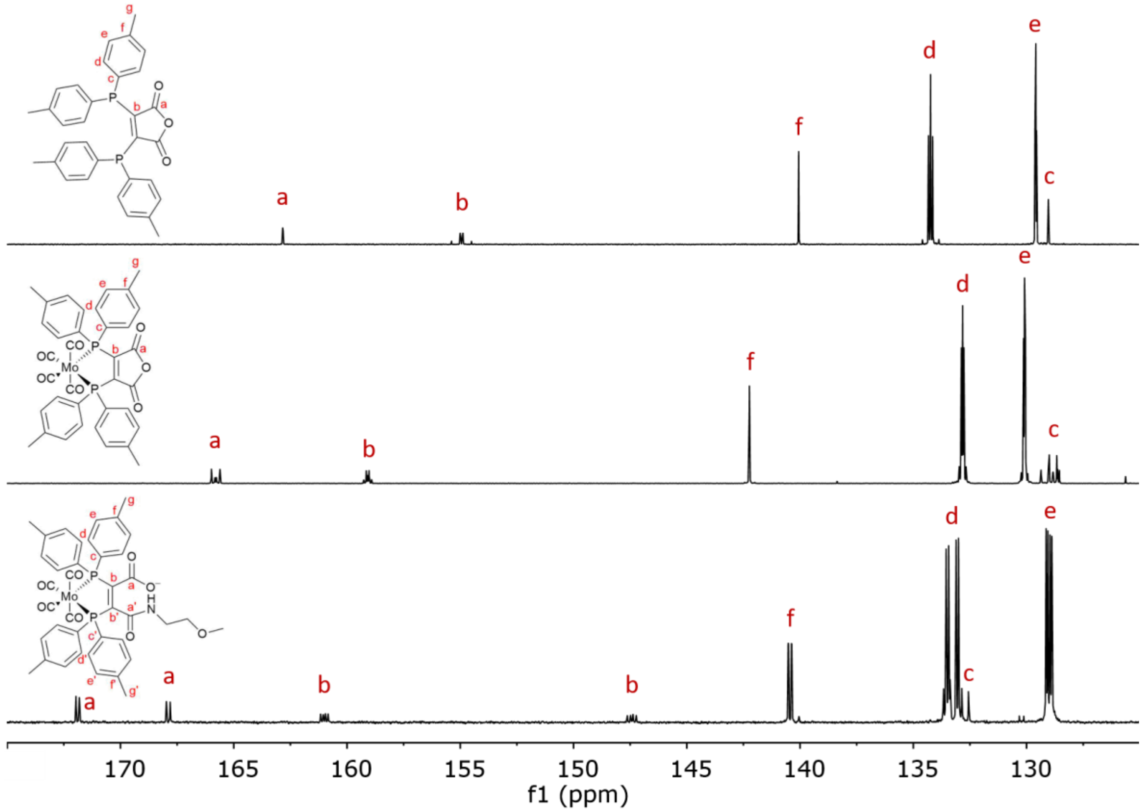

Figure S6: ^{13}C NMR signals for DP^{Tol} in chloroform-d, and of $[Mo^0(CO)_4(DP^{Tol})]$ and $(RNH_3)[Mo^0(CO)_4(DP^{Tol}$-$NHR)]$ (R = $-CH_2CH_2OCH_3$/MOE) in dichloromethane-d_2.

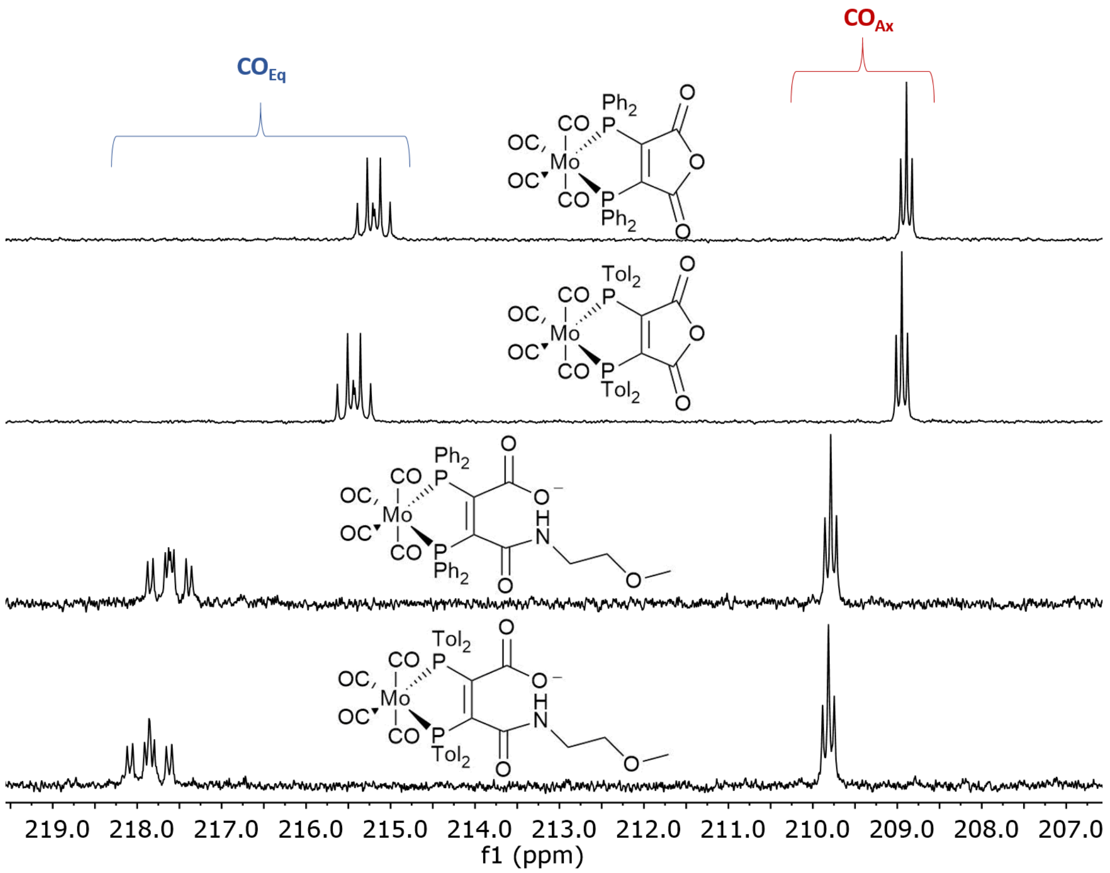

Figure S7: ^{13}C NMR signals for metal carbonyls in $[Mo^0(CO)_4(DP)]$ and $(RNH_3)[Mo^0(CO)_4(DP\text{-}NHR)]$ (DP = DP^{Ph} or DP^{Tol}; R = $-CH_2CH_2OCH_3$) in dichloromethane-d_2.

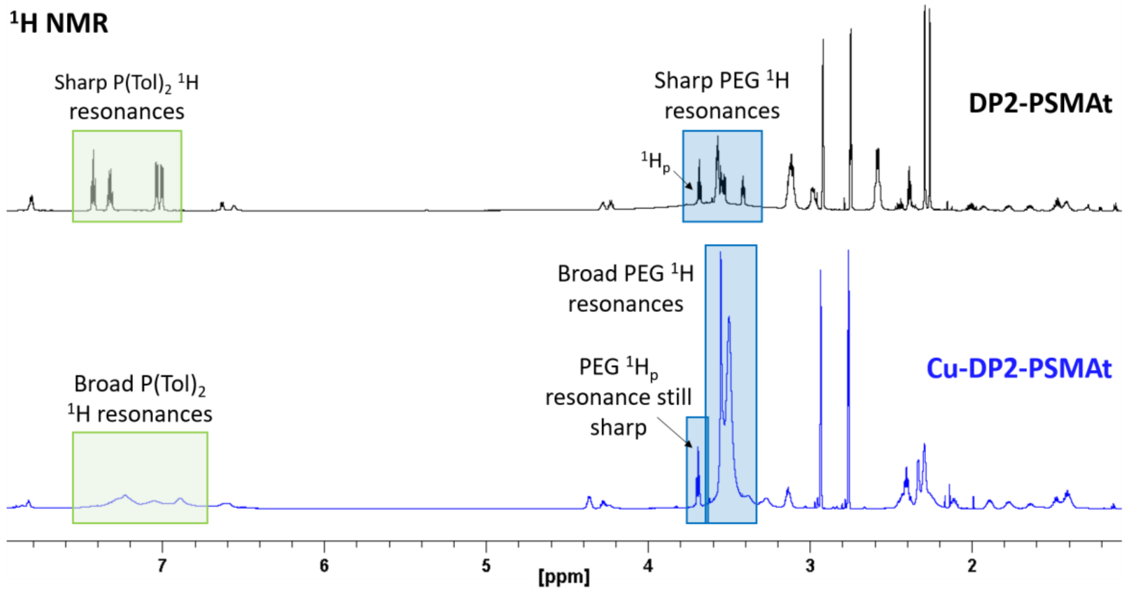

Figure S8: Comparative ^{1}H NMR spectrum of DP^{Tol}-PSMAt and $[Cu(DP^{Tol}\text{-}PSMAt)_2]^+$ in DMF-d_7 with DIPEA base. Coordination leads to broadening of signals corresponding to protons in close proximity to the Cu(I) center (aromatic signals, the two p-tolyl CH_3 peaks at approx. 2.3 ppm, and most PEG-linker protons).

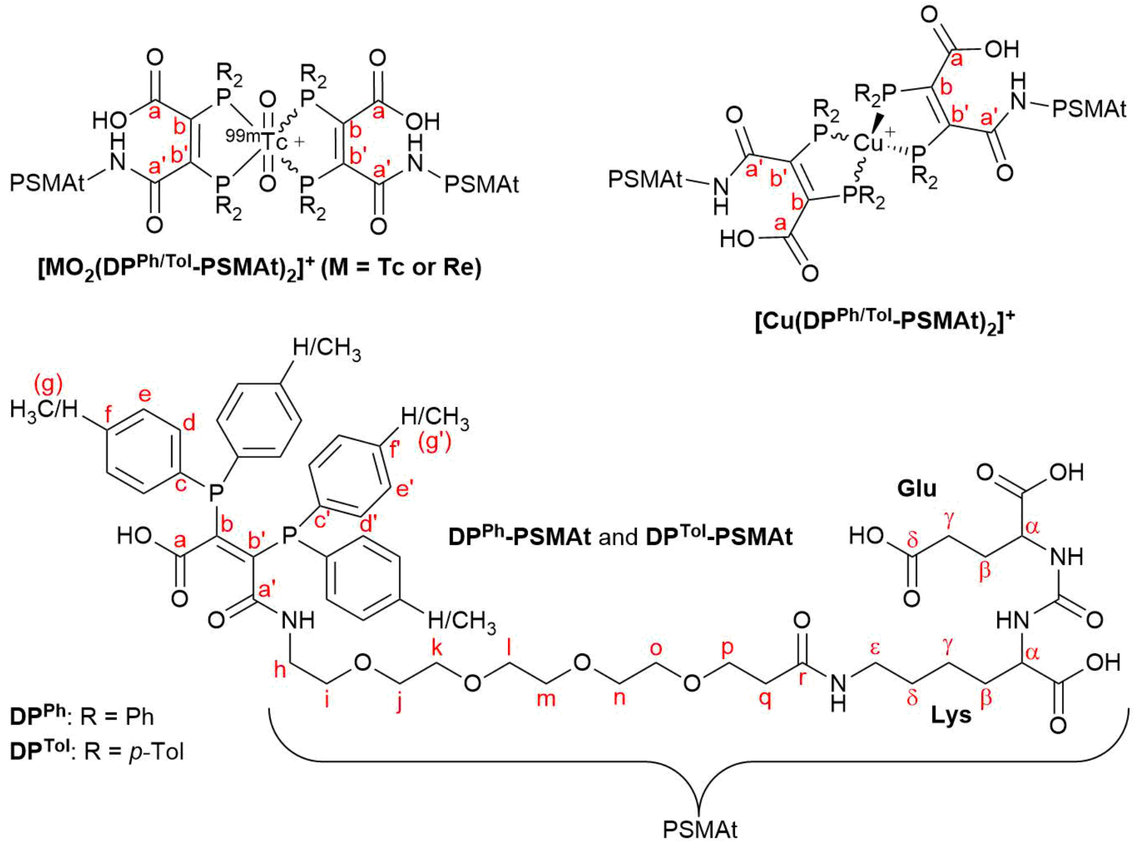

Figure S9: General structure of [ReO₂(DP^{Ph/Tol}-PSMAt)₂]⁺ (top left), [Cu(DP^{Ph/Tol}-PSMAt)₂]⁺ (top right) and of the attached DP^{Ph}-PSMAt and DP^{Tol}-PSMAt ligands (bottom) for NMR assignments.

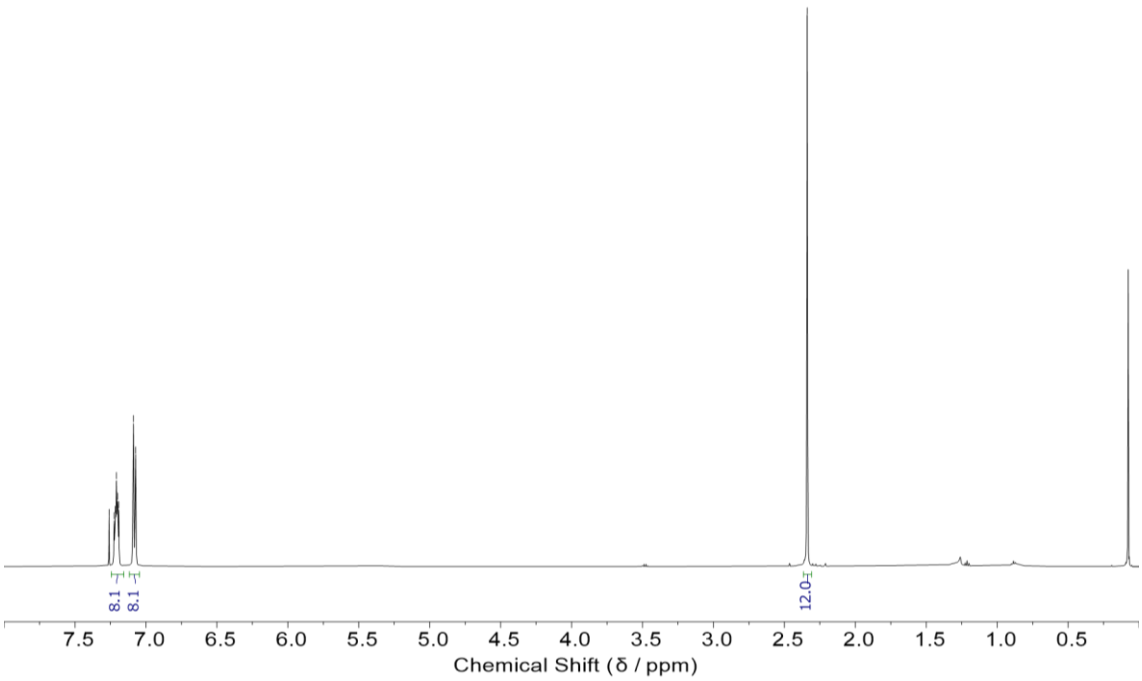

Figure S10: ^{1}H NMR spectrum of DP^{Tol} in chloroform-d.

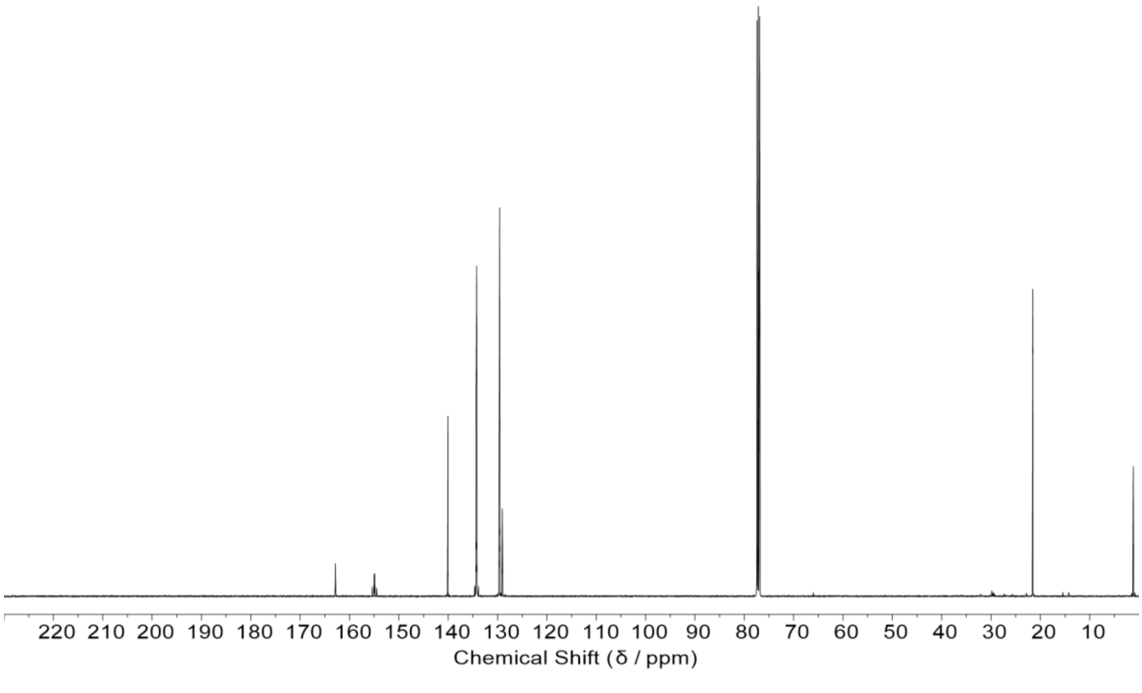

Figure S11: ^{13}C NMR spectrum of DP^{Tol} in chloroform-d.

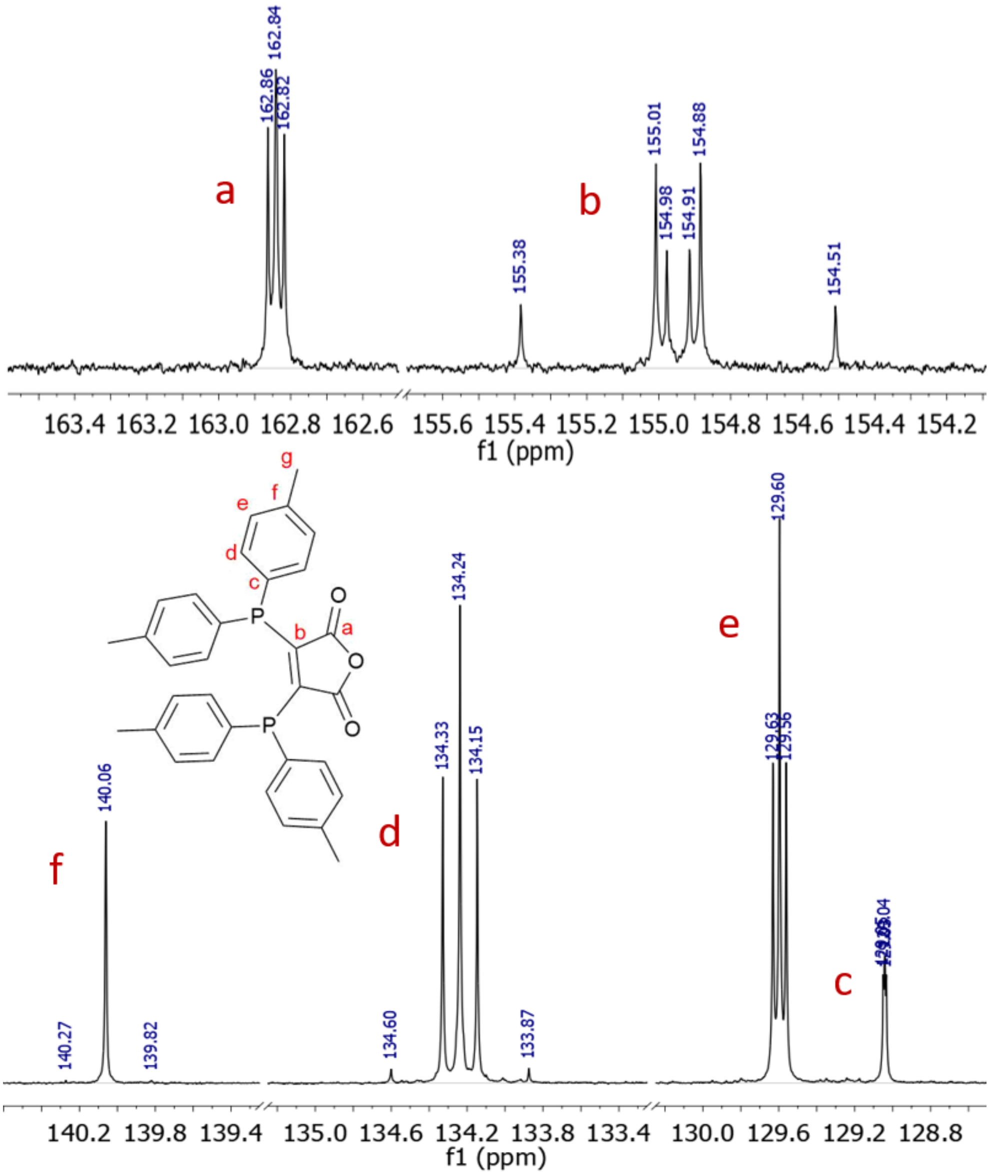

Figure S12: Magnifications of the sp^2-carbon signals in the ^{13}C NMR spectrum of DP^{Tol} in chloroform-d.

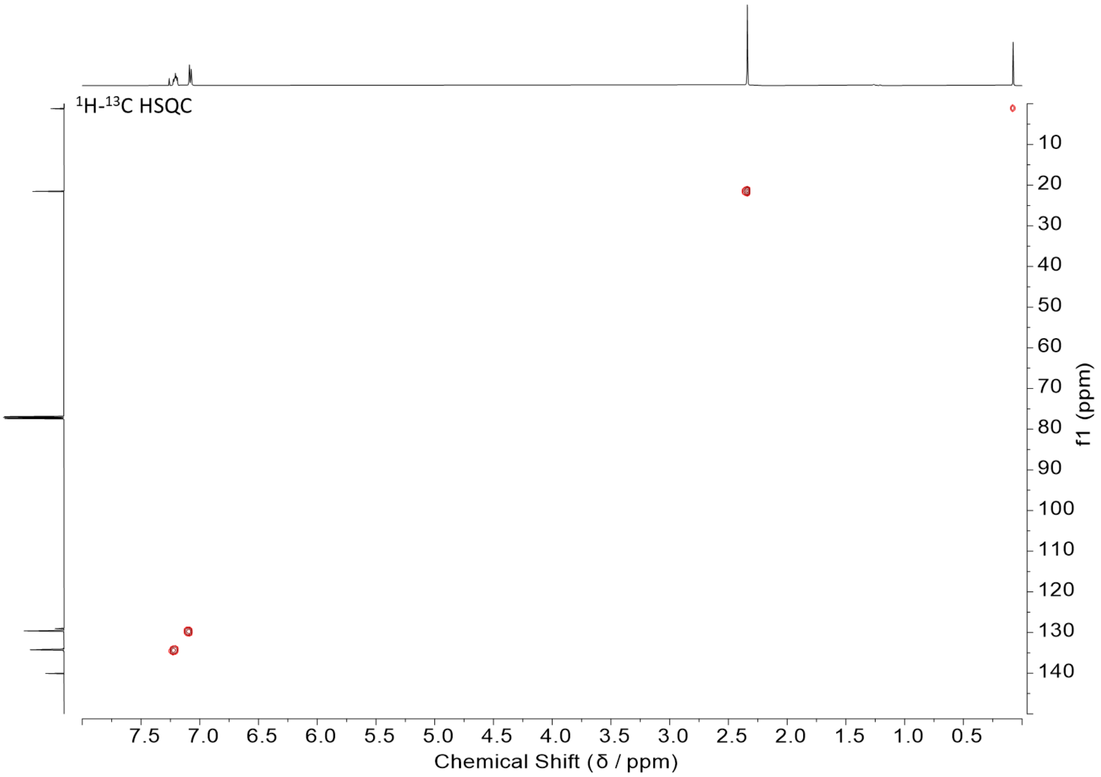

Figure S13: ^{1}H-^{13}C HSQC NMR spectrum of DP^{Tol} in chloroform-d.

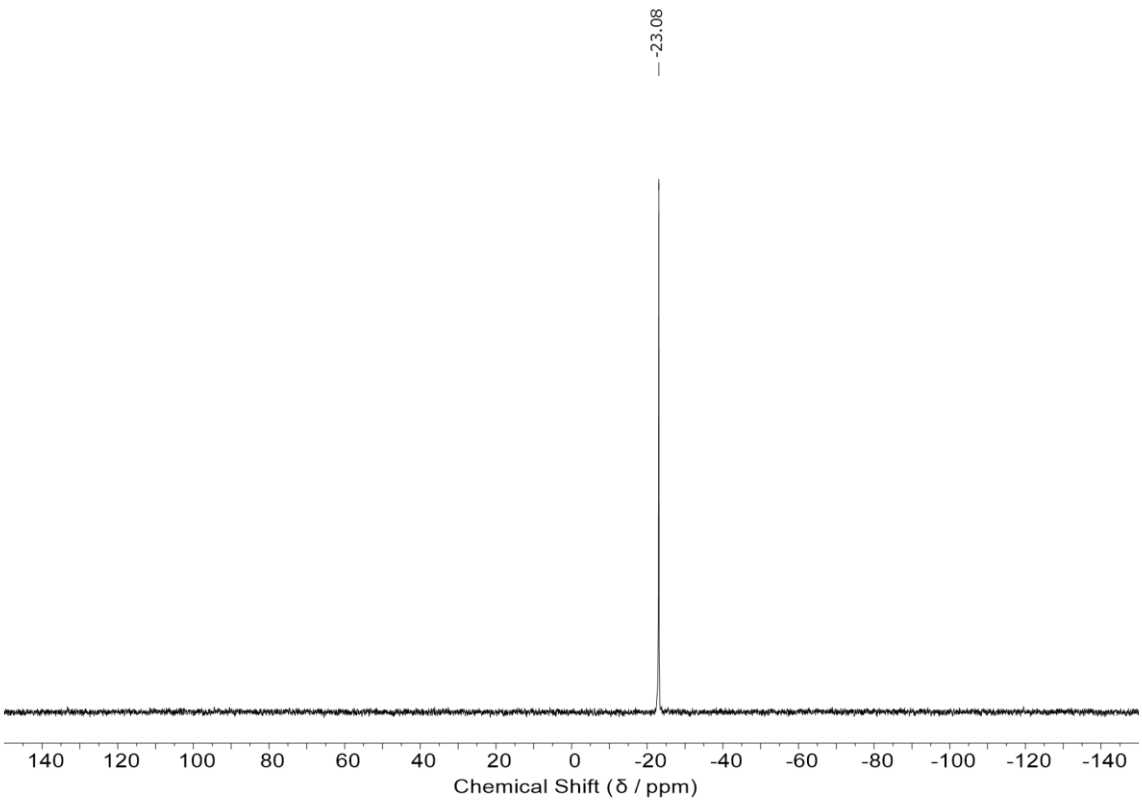

Figure S14: ^{31}P{^{1}H} NMR spectrum of DP^{Tol} in chloroform-d.

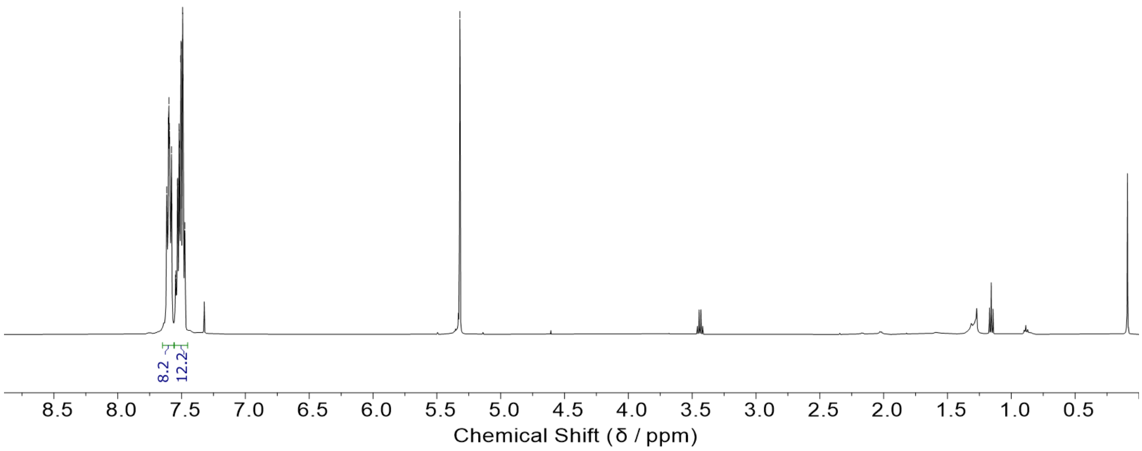

Figure S15: ^{1}H NMR spectrum of [Mo(CO)$_4$(DP^{Ph})] in dichloromethane-d$_2$.

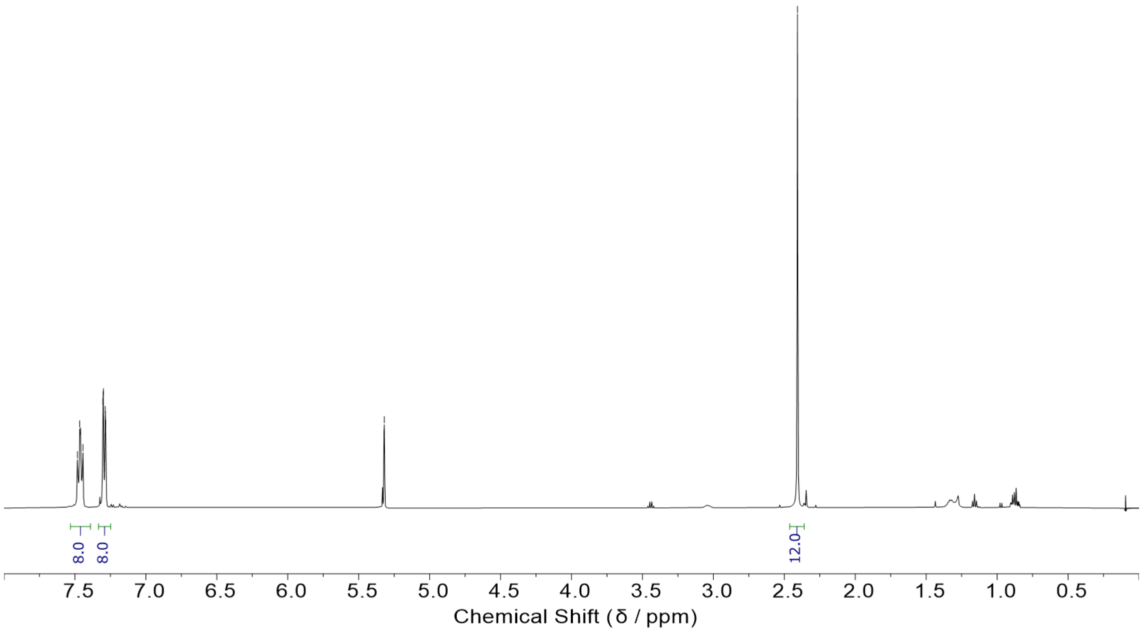

Figure S16: ^{1}H NMR spectrum of [Mo(CO)$_4$(DP^{Tol})] in dichloromethane-d$_2$.

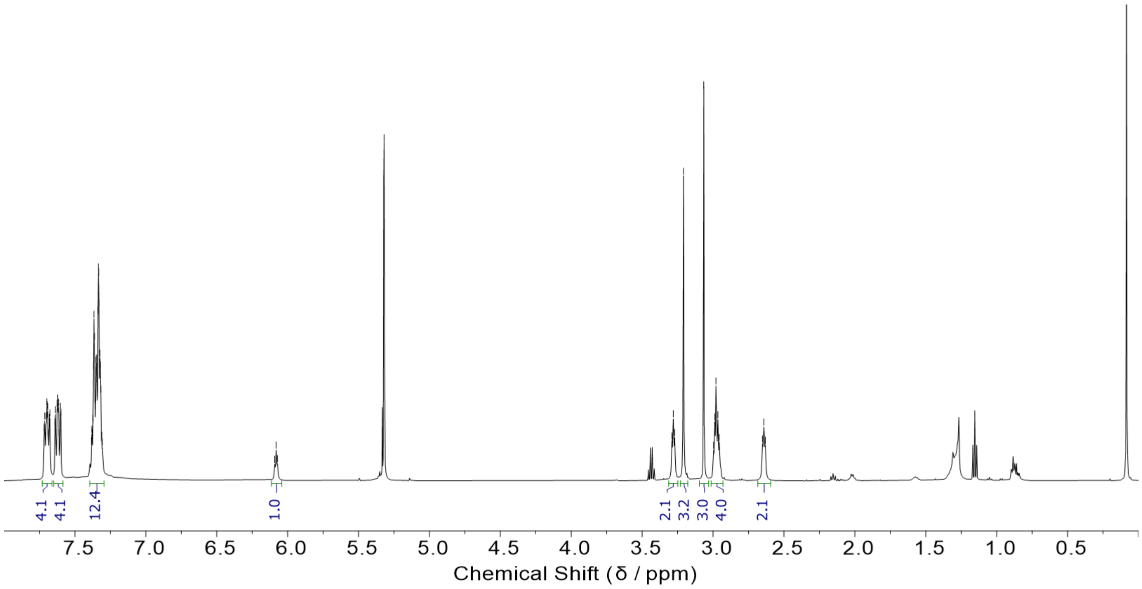

Figure S17: ^{1}H NMR spectrum of (RNH$_3$)[Mo(CO)$_4$(DP^{Ph}-NHR)] (R = -CH$_2$CH$_2$OCH$_3$) in dichloromethane-d$_2$.

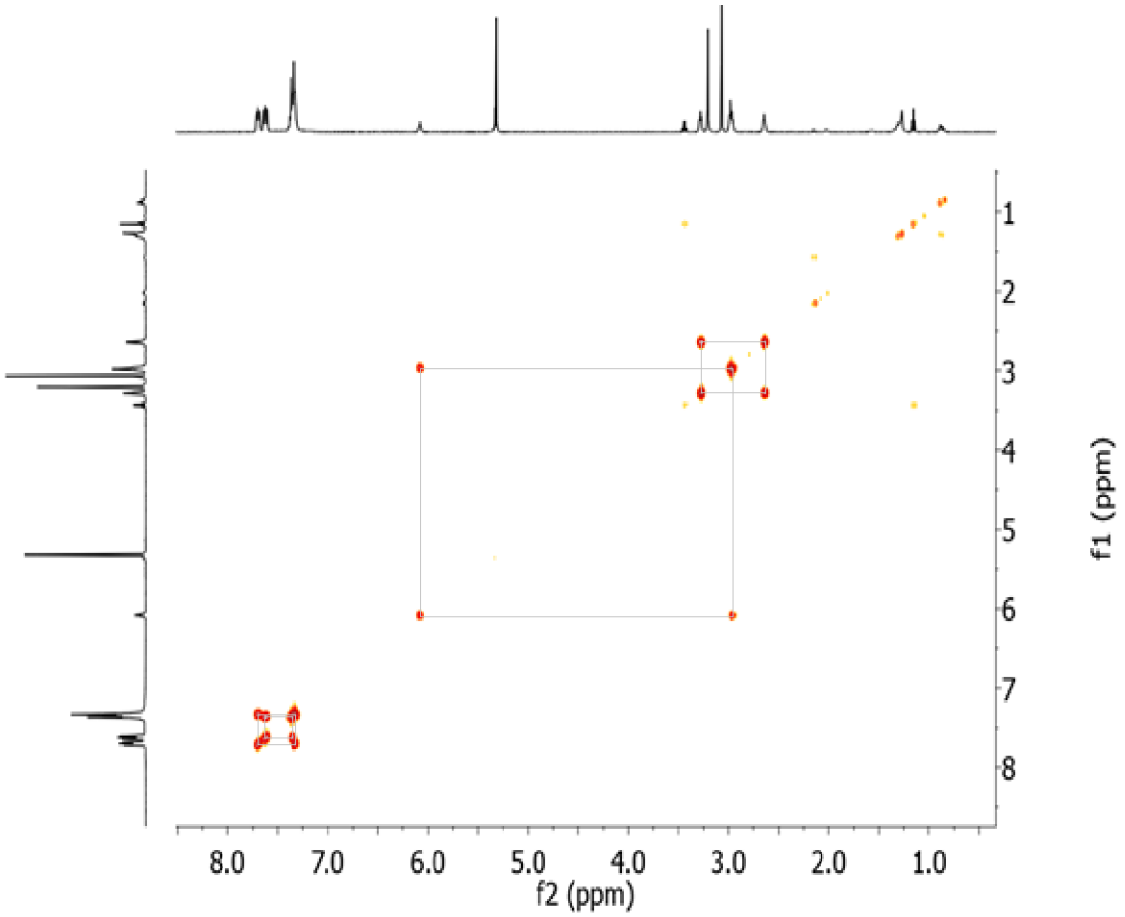

Figure S18: ^{1}H-^{1}H-TOCSY NMR spectrum of (RNH$_3$)[Mo(CO)$_4$(DP^{Ph}-NHR)] (R = -CH$_2$CH$_2$OCH$_3$) in dichloromethane-d$_2$.

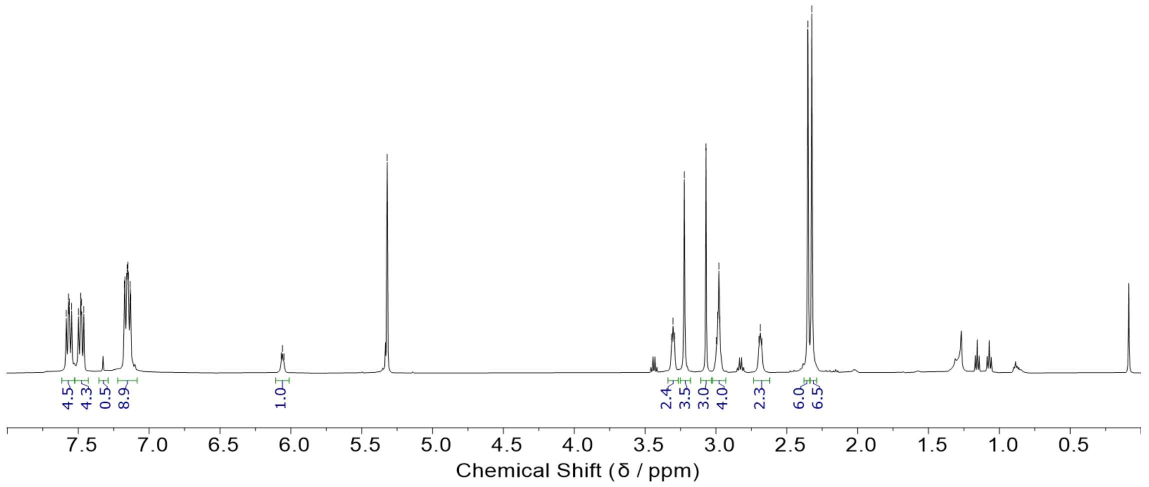

Figure S19: ^{1}H NMR spectrum of (RNH$_3$)[Mo(CO)$_4$(DP^{Ph}-NHR)] (R = -CH$_2$CH$_2$OCH$_3$) in dichloromethane-d$_2$.

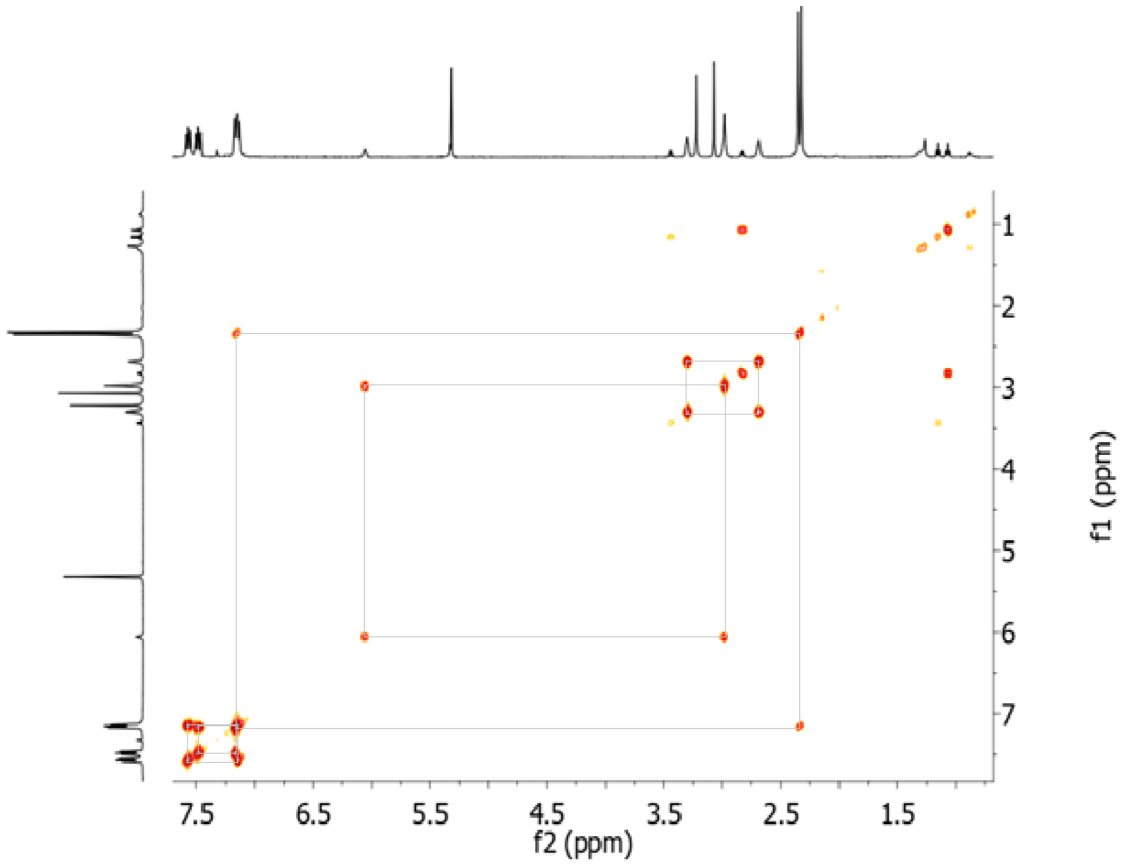

Figure S20: ^{1}H-^{1}H-TOCSY NMR spectrum of (RNH$_3$)[Mo(CO)$_4$(DP^{Tol}-NHR)] (R = -CH$_2$CH$_2$OCH$_3$) in dichloromethane-d$_2$.

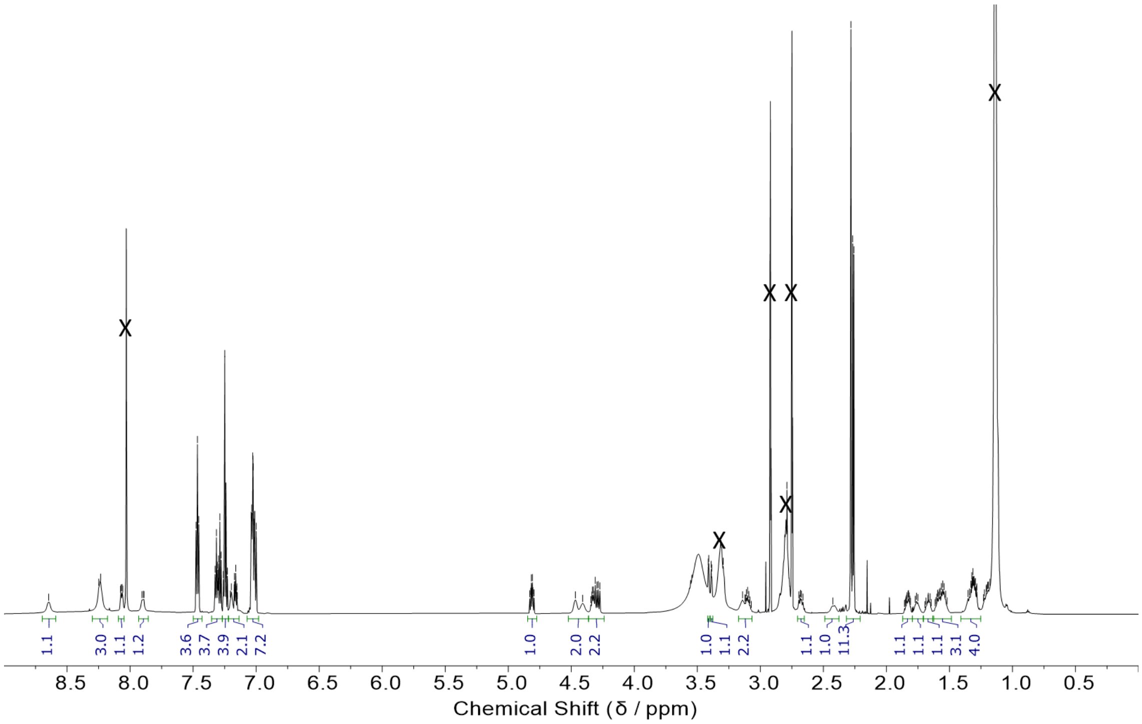

Figure S21: [1]*H NMR spectrum of DP*^{Tol}*-RGD in DMF-d*$_7$ *with DIPEA base. Crossed-out signals correspond to either DIPEA {δ (ppm) 1.02-1.09 (m), 2.54 (q), 3.08 (p)} or DMF {δ (ppm) 2.75(p), 2.92 (p), 8.03 (s)}.*

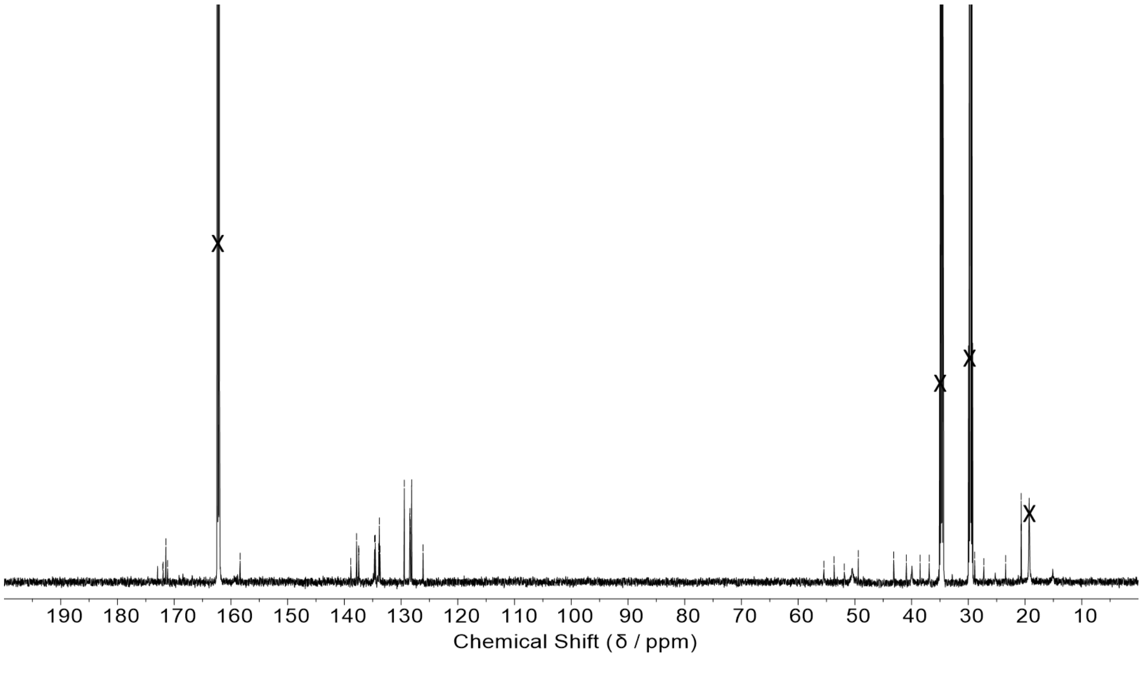

Figure S22: ^{13}*C NMR spectrum of DP*^{Tol}*-RGD in DMF-d*$_7$ *with DIPEA base. Crossed-out signals correspond to either DMF {δ (ppm) 162 (t), 35 (heptet) and 30 (heptet)} or DIPEA {δ (ppm) 20}, while smaller DIPEA peaks {δ (ppm) 39, 49 and 16} were not marked.*

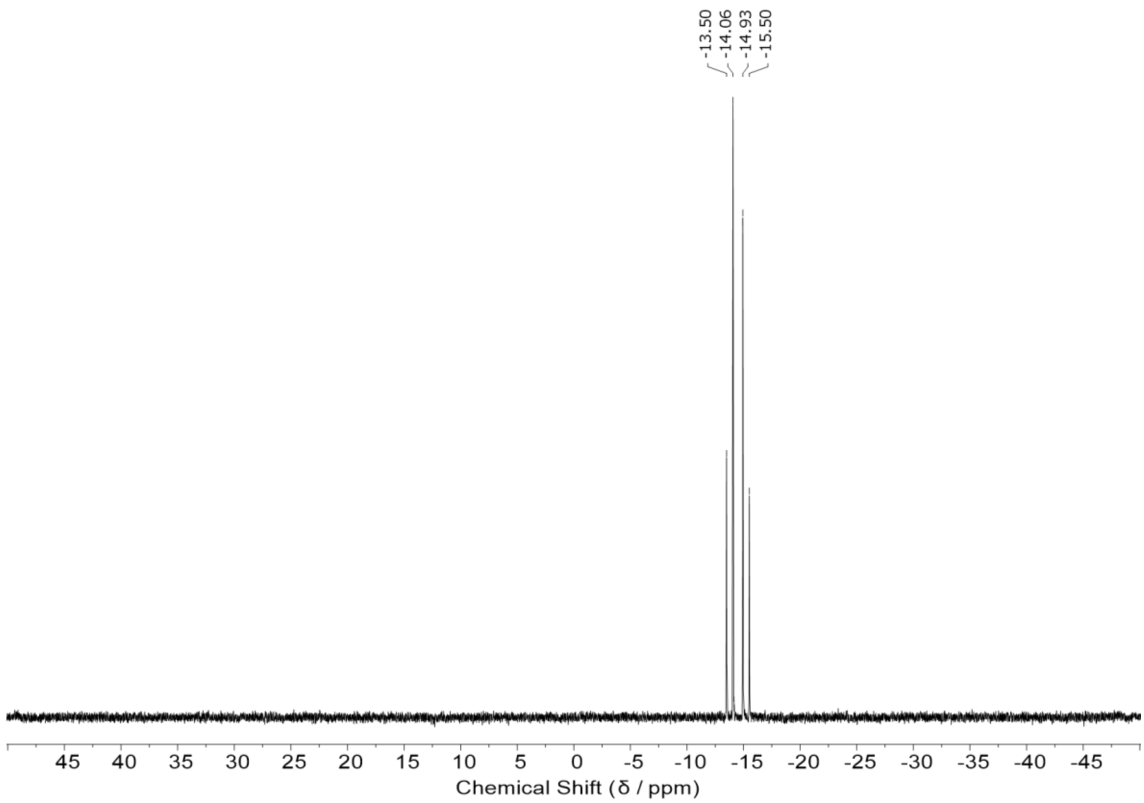

Figure S23: $^{31}P\{^1H\}$ NMR spectrum of DP^{Tol}-RGD in DMF-d_7 with DIPEA base.

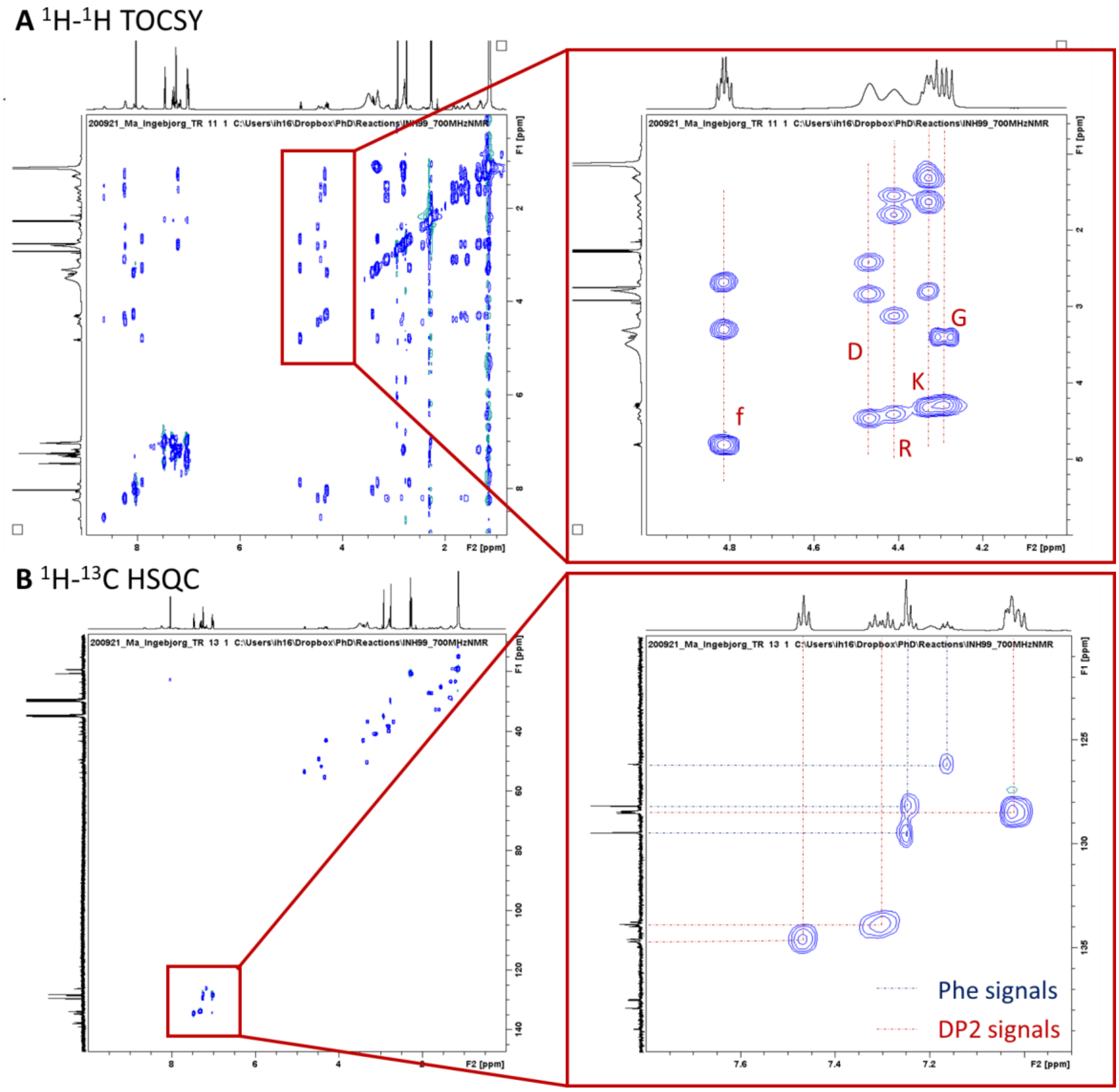

Figure S24: ^{1}H-^{1}H-TOCSY and ^{1}H-^{13}C HSQC NMR spectra of DP[Tol]-RGD in DMF-d₇ with DIPEA base.

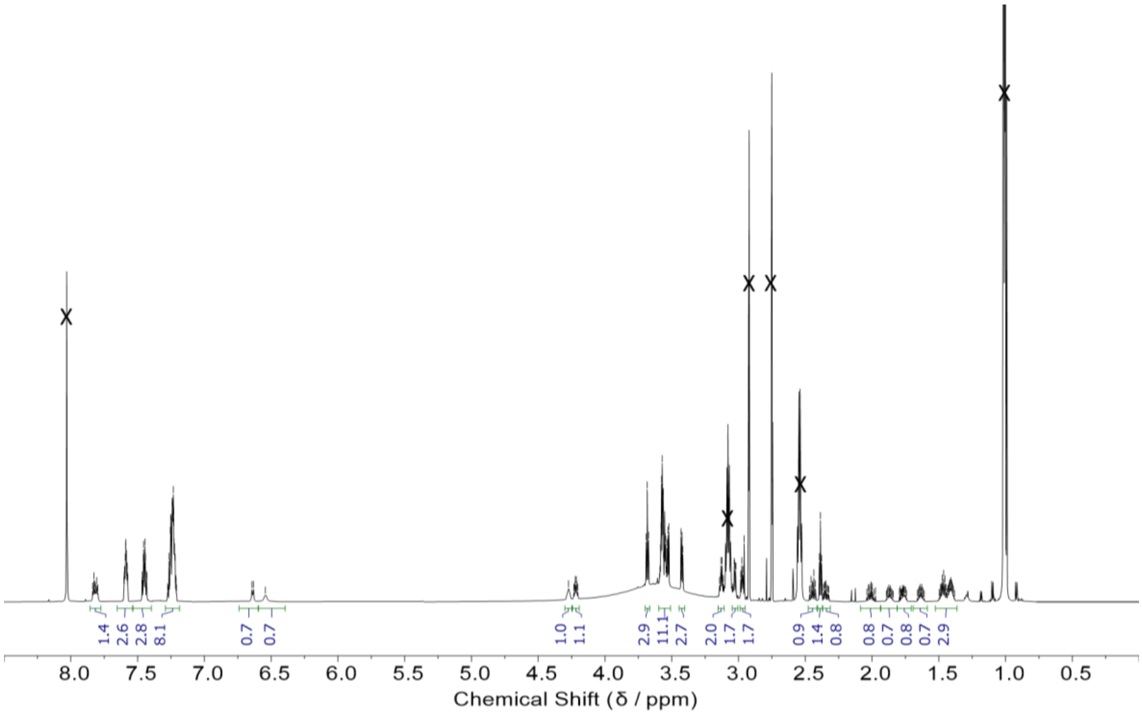

Figure S25: ^{1}H NMR spectrum of DP[Ph]-PSMAt in DMF-d7 with DIPEA base. Crossed-out signals correspond to either DIPEA {δ (ppm) 1.02-1.09 (m), 2.54 (q), 3.08 (p)} or DMF {δ (ppm) 2.75(p), 2.92 (p), 8.03 (s)}.

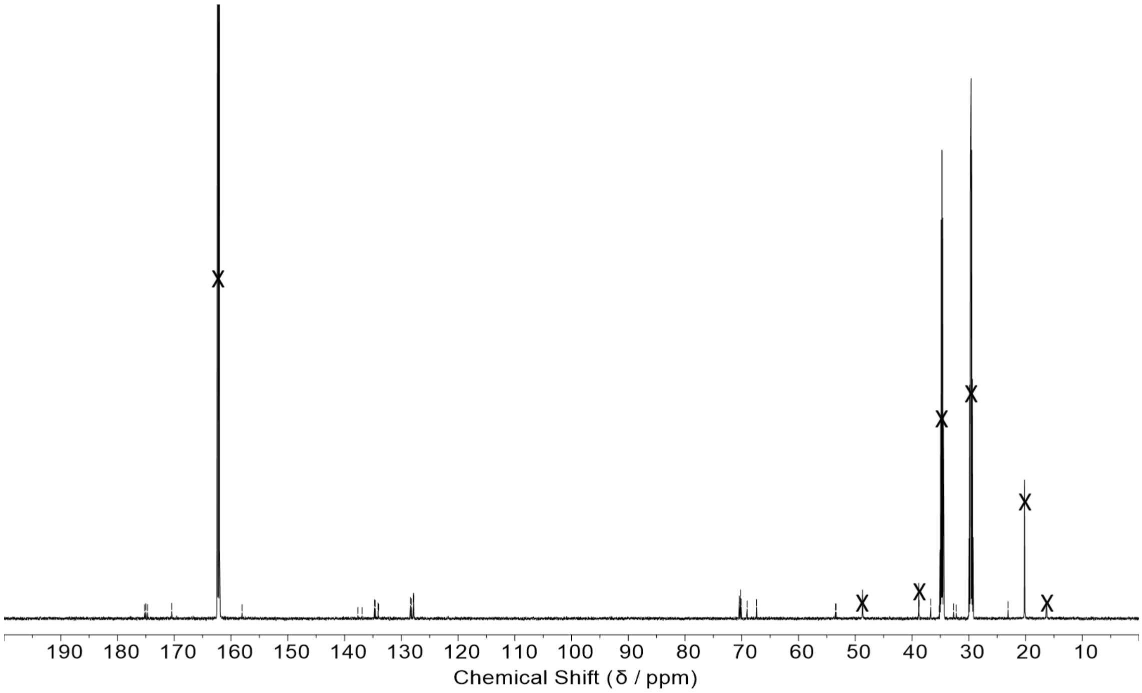

Figure S26: ^{13}C NMR spectrum of DP[Ph]-PSMAt in DMF-d7 with DIPEA base. Crossed-out signals correspond to either DIPEA {δ (ppm) 39, 49, 20 and 16} or DMF {δ (ppm) 162 (t), 35 (heptet) and 30 (heptet)}.

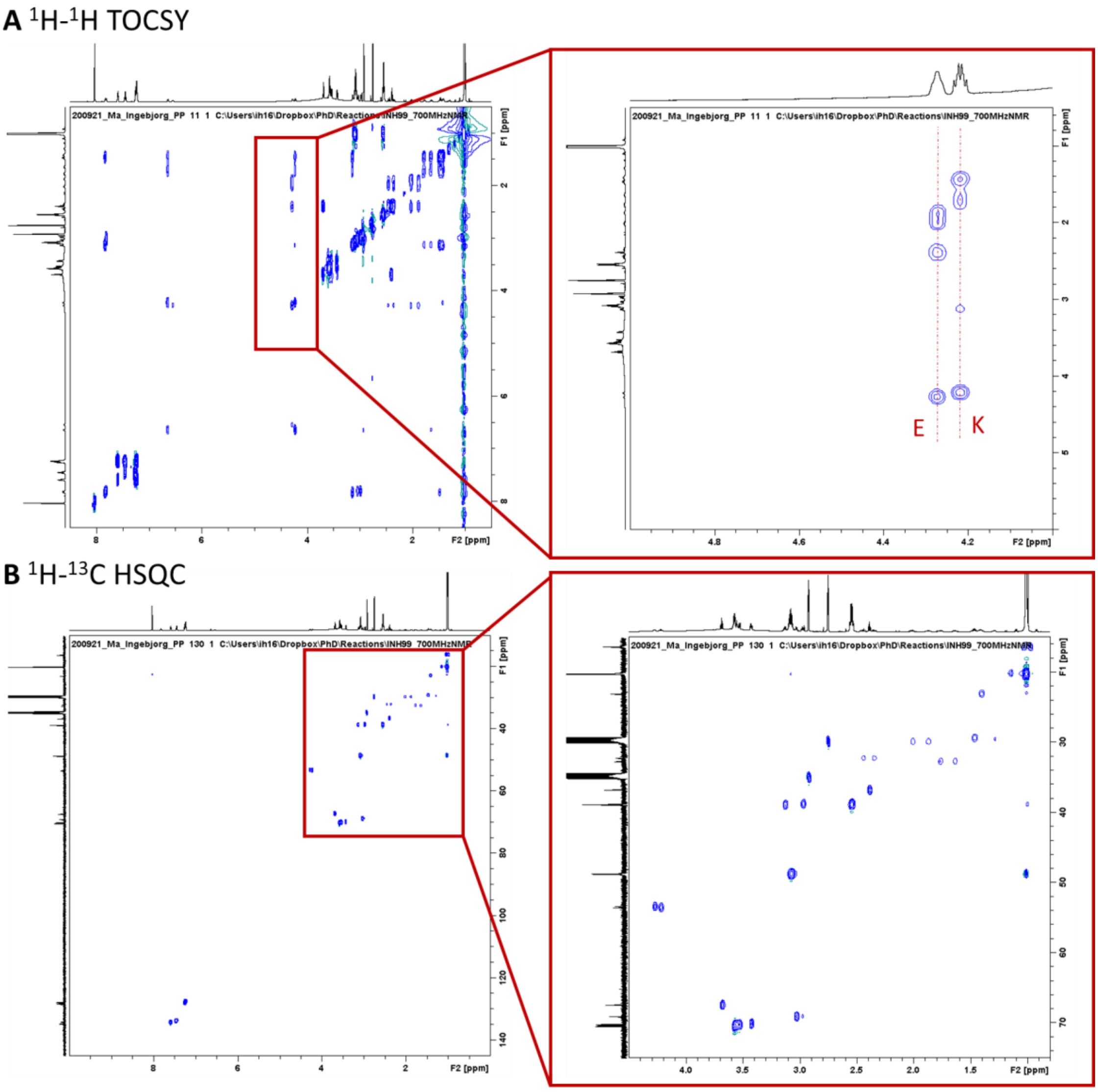

Figure S27: ^{1}H-^{1}H-TOCSY and ^{1}H-^{13}C HSQC NMR spectrum of DP^{Ph}-PSMAt in DMF-d_7 with DIPEA base.

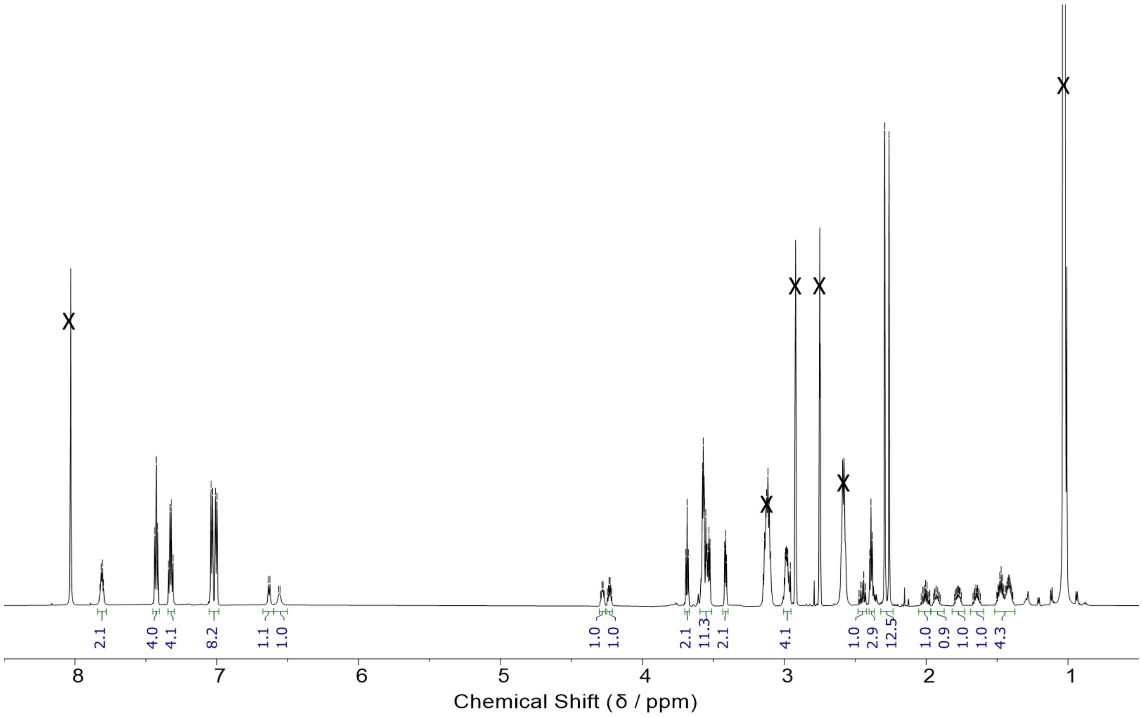

Figure S28: ¹H NMR spectrum of DP^{Tol}-PSMAt in DMF-d$_7$ with DIPEA base. Crossed-out signals correspond to either DIPEA {δ (ppm) 1.02-1.09 (m), 2.54 (q), 3.08 (p)} or DMF {δ (ppm) 2.75(p), 2.92 (p), 8.03 (s)}.

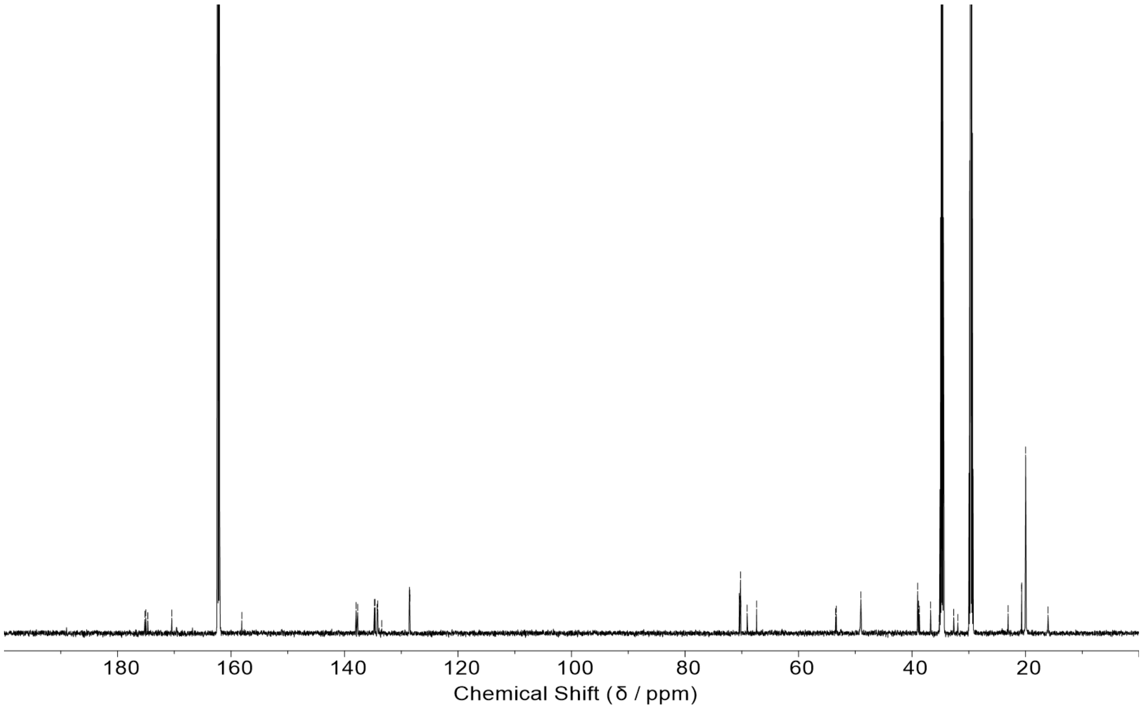

Figure S29: ¹³C NMR spectrum of DP^{Tol}-PSMAt in DMF-d$_7$ with DIPEA base.

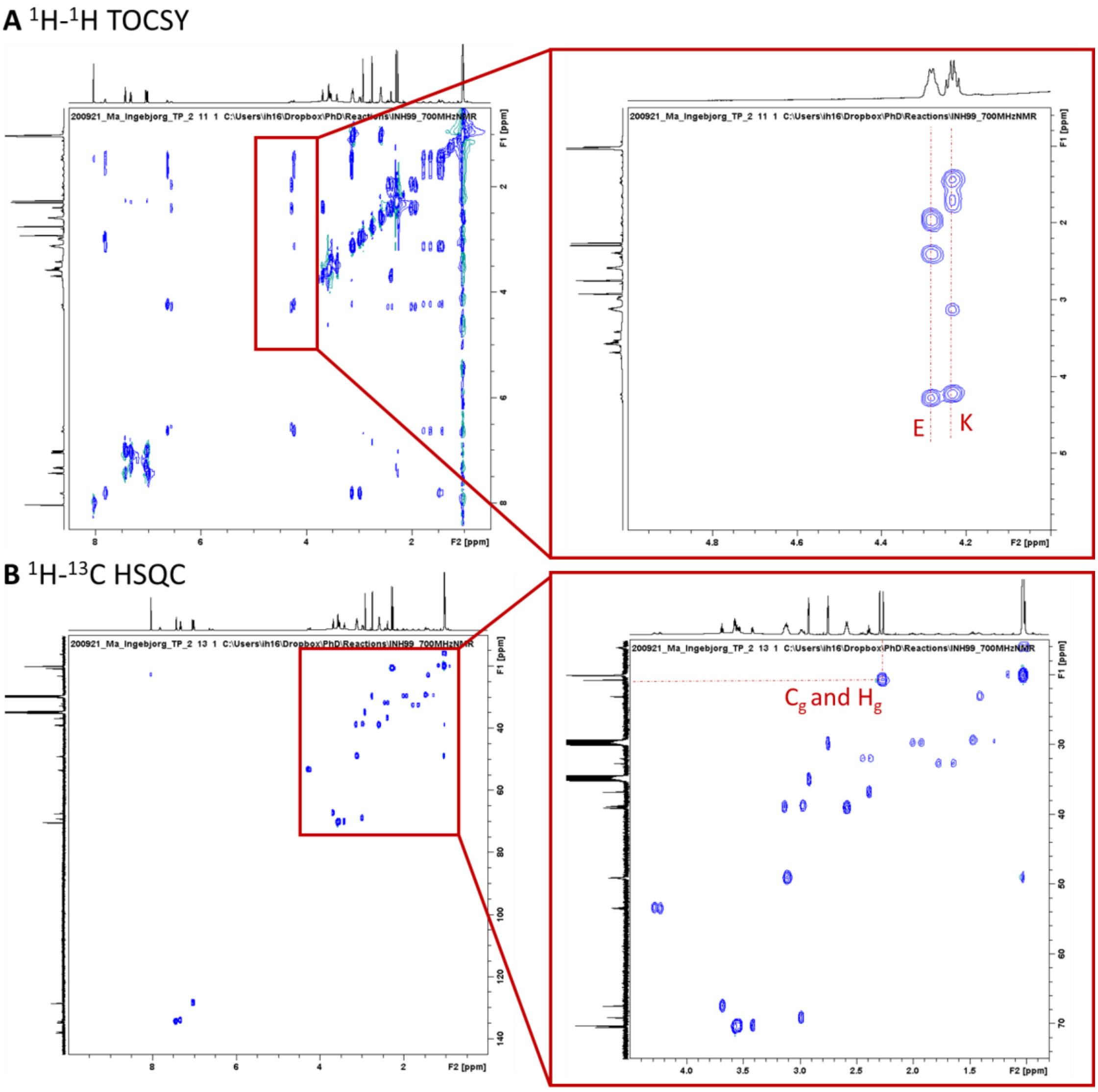

Figure S30: ^{1}H-^{1}H-TOCSY and ^{1}H-^{13}C HSQC NMR spectrum of DP^{Tol}-PSMAt in DMF-d_7 with DIPEA base.

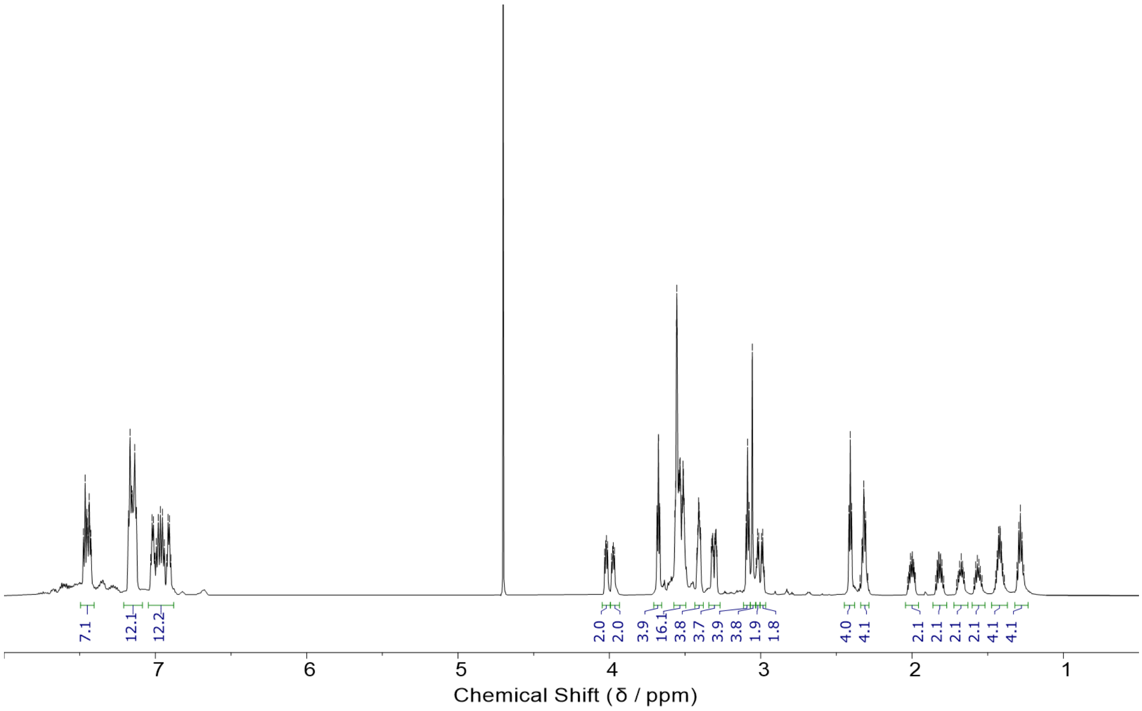

Figure S31: ^{1}H NMR spectrum of $[ReO_2(DP^{Ph}\text{-}PSMAt)_2]^+$ in D_2O with 33 mM ammonium acetate-d_7.

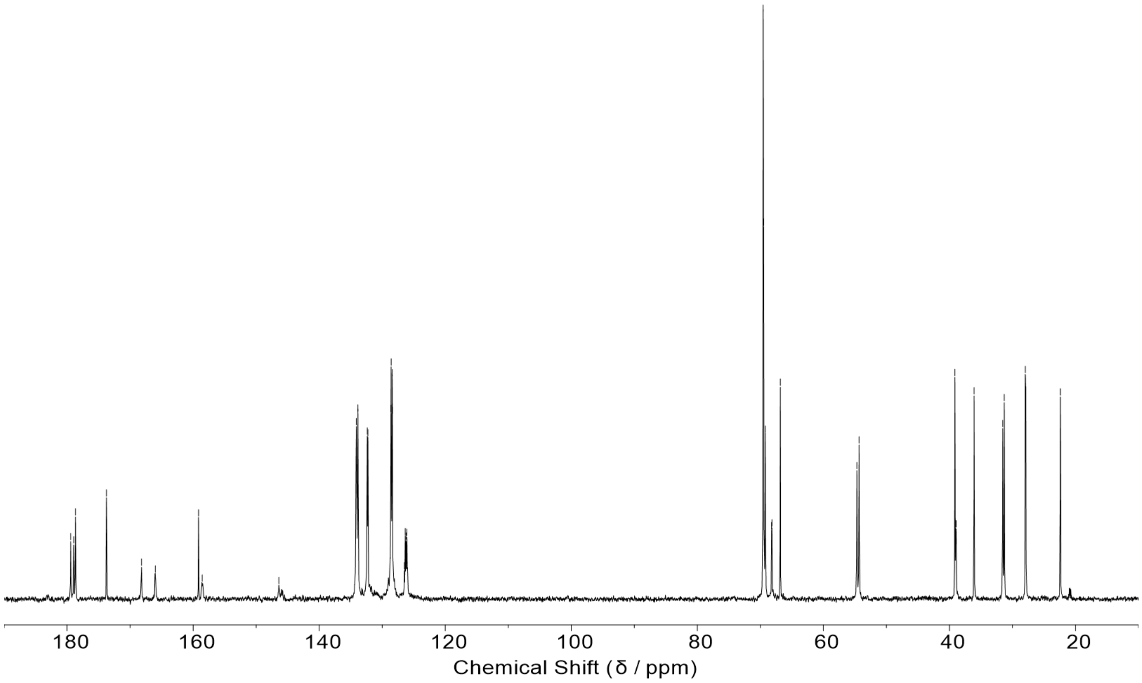

Figure S32: ^{13}C NMR spectrum of $[ReO_2(DP^{Ph}\text{-}PSMAt)_2]^+$ in D_2O with 33 mM ammonium acetate-d_7.

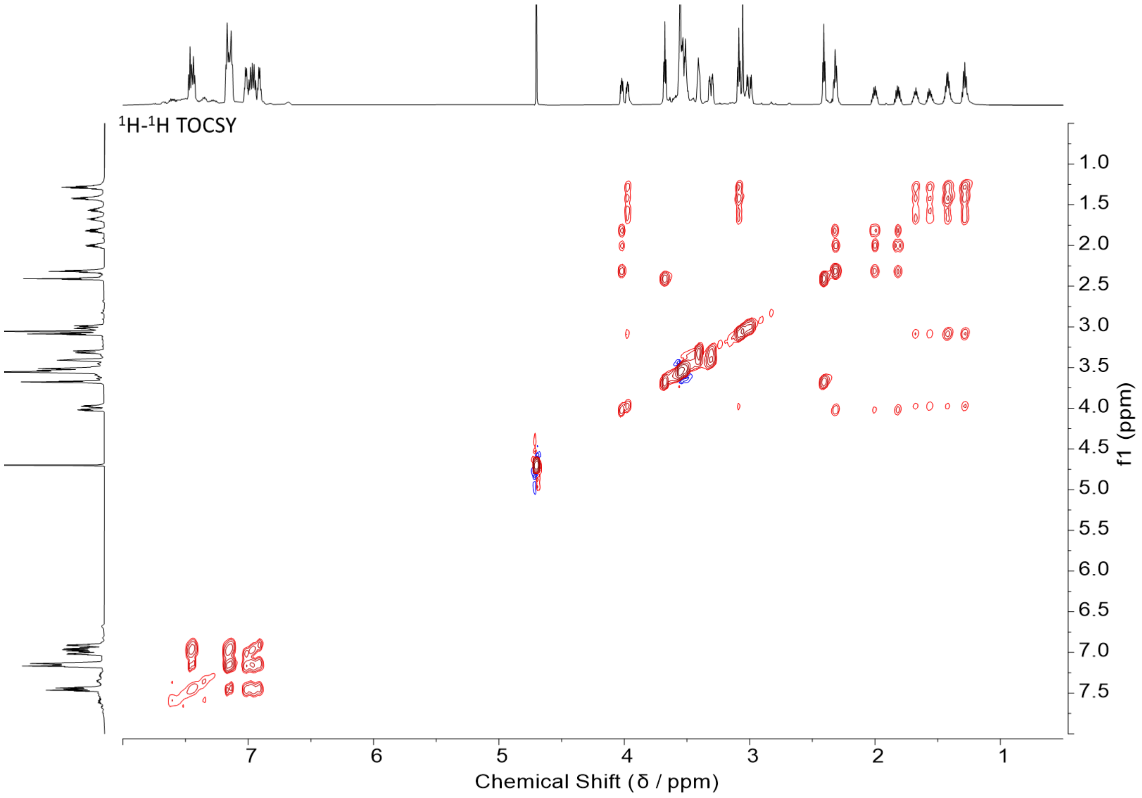

Figure S33: ¹H-¹H-TOCSY NMR spectrum of $[ReO_2(DP^{Ph}\text{-}PSMAt)_2]^+$ in D_2O with 33 mM ammonium acetate-d_7.

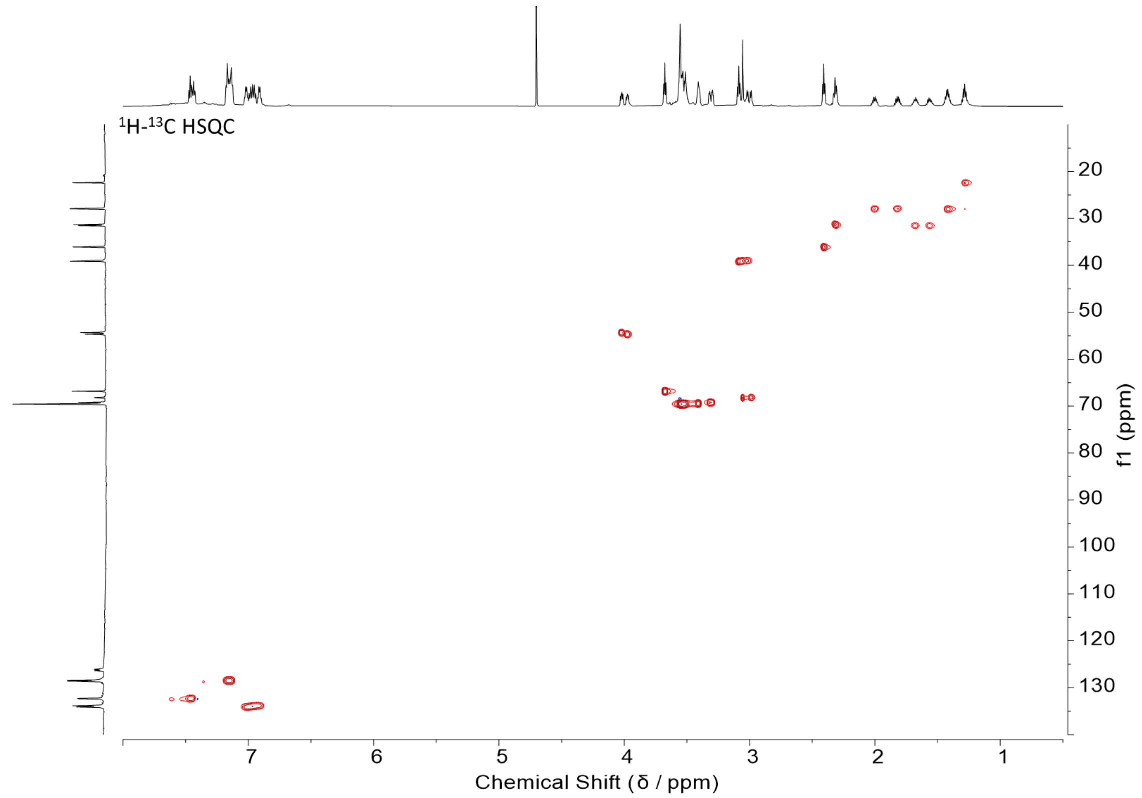

Figure S34: ¹H-¹³C HSQC NMR spectrum of $[ReO_2(DP^{Ph}\text{-}PSMAt)_2]^+$ in D_2O with 33 mM ammonium acetate-d_7.

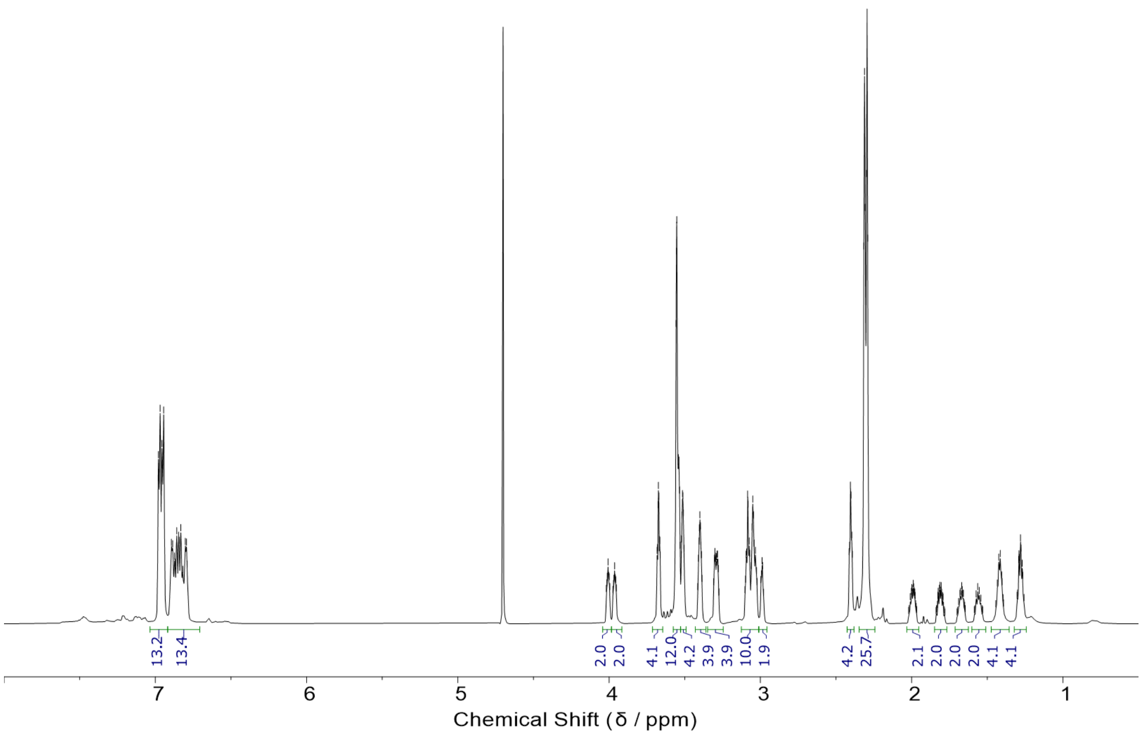

Figure S35: ^{1}H NMR spectrum of $[ReO_2(DP^{Tol}\text{-}PSMAt)_2]^+$ in D_2O with 33 mM ammonium acetate-d_7.

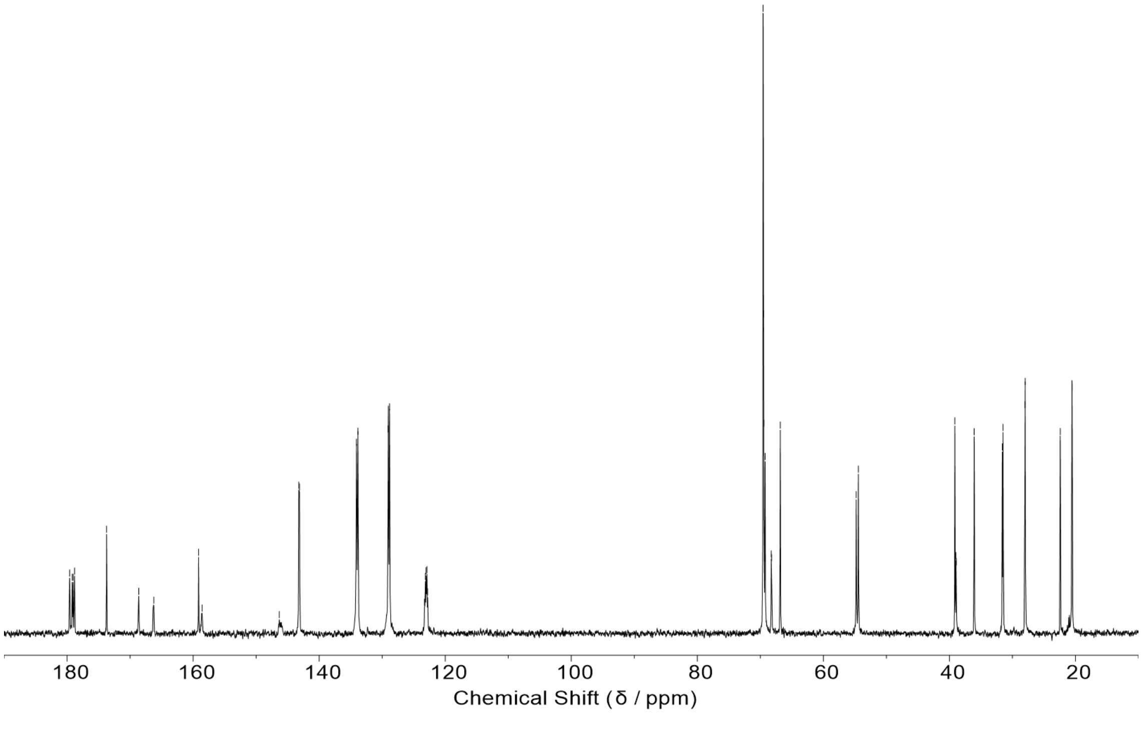

Figure S36: ^{13}C NMR spectrum of $[ReO_2(DP^{Tol}\text{-}PSMAt)_2]^+$ in D_2O with 33 mM ammonium acetate-d_7.

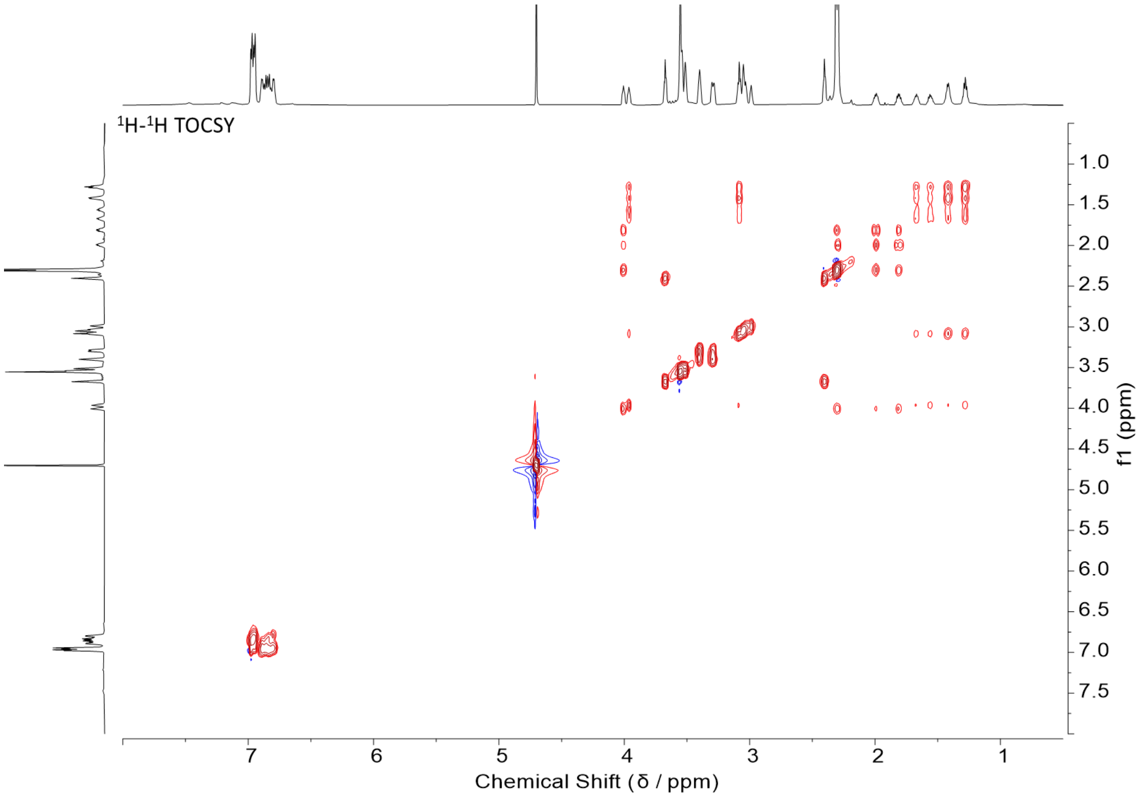

Figure S37: ^1H-^1H-TOCSY NMR spectrum of $[ReO_2(DP^{Tol}\text{-}PSMAt)_2]^+$ in D_2O with 33 mM ammonium acetate-d_7.

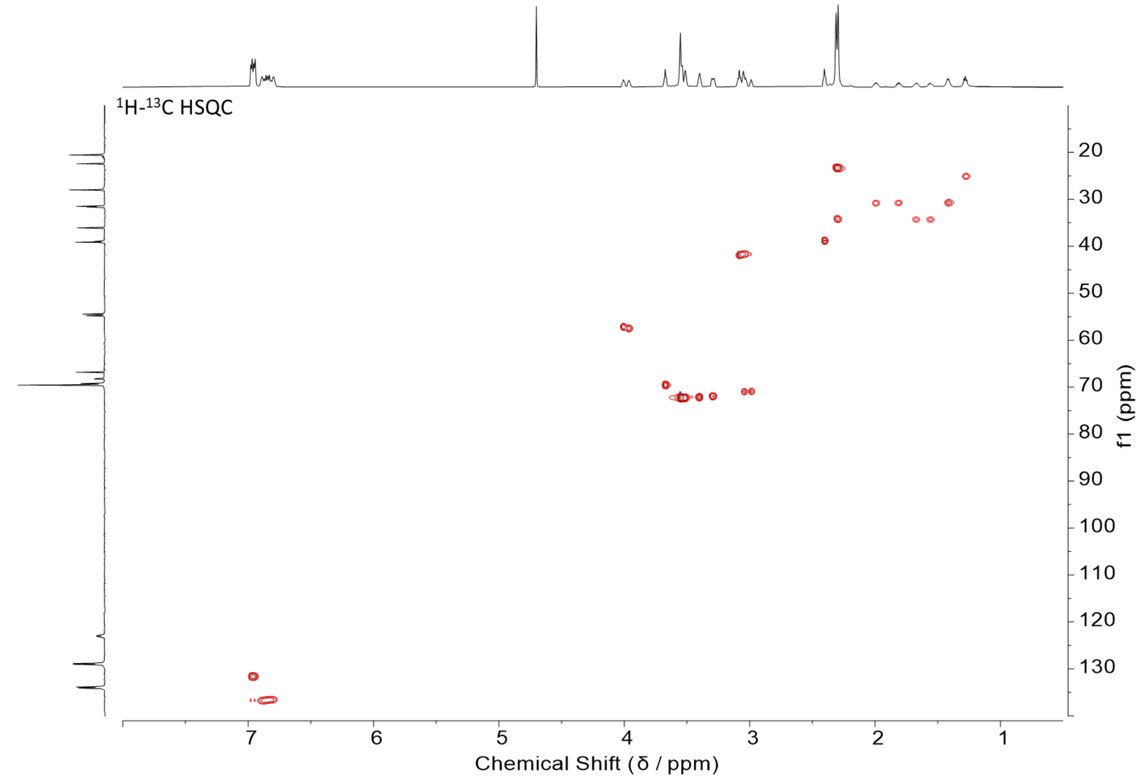

Figure S38: ^1H-^{13}C HSQC NMR spectrum of $[ReO_2(DP^{Tol}\text{-}PSMAt)_2]^+$ in D_2O with 33 mM ammonium acetate-d_7.

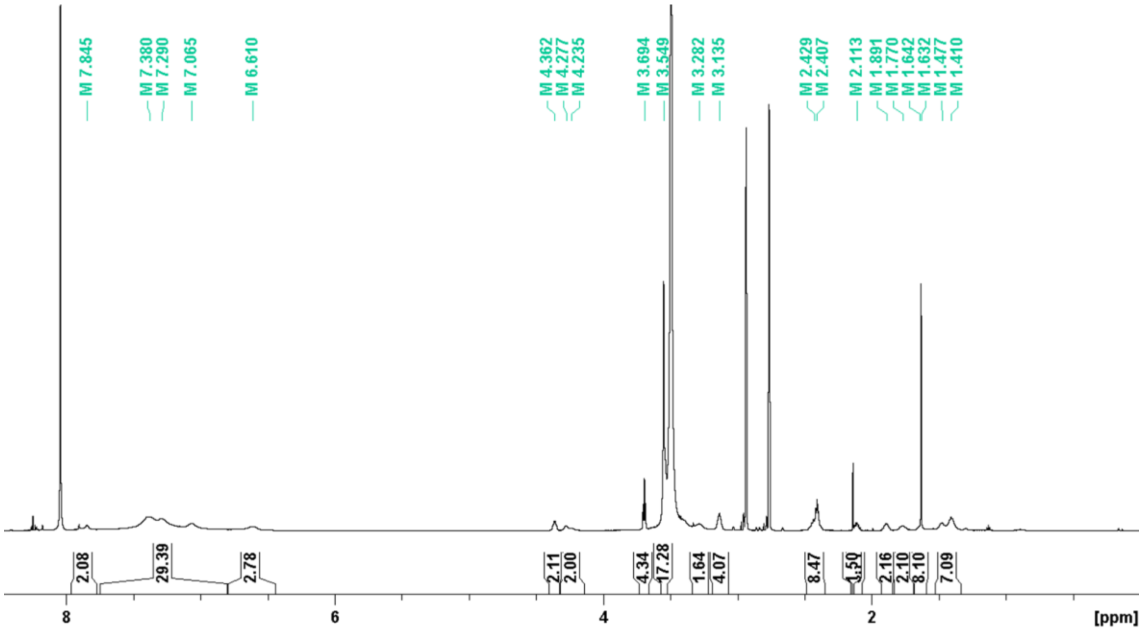

Figure S39: ^{1}H NMR spectrum of [Cu(DP^{Ph}-PSMAt)${}_{2}$]${}^{+}$ in DMF-d${}_{7}$ with DIPEA base.

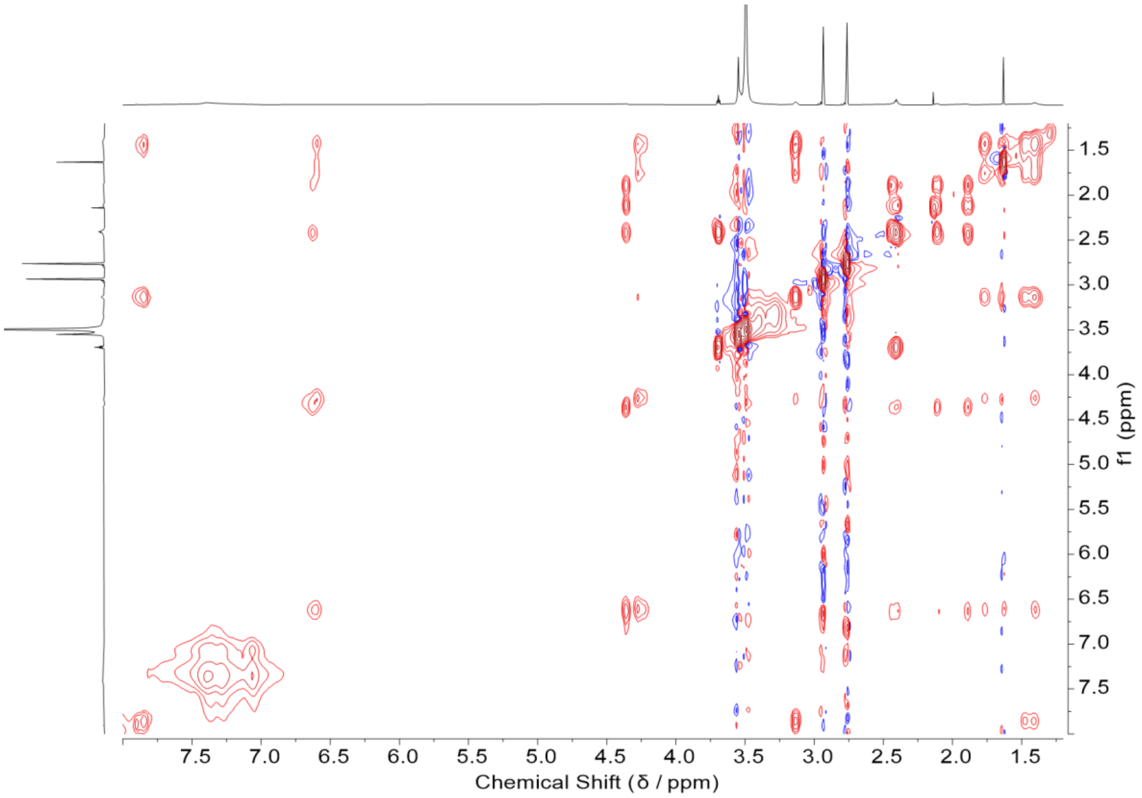

Figure S40: ^{1}H-^{1}H-TOCSY NMR spectrum of [Cu(DP^{Ph}-PSMAt)${}_{2}$]${}^{+}$ in DMF-d${}_{7}$ with DIPEA base.

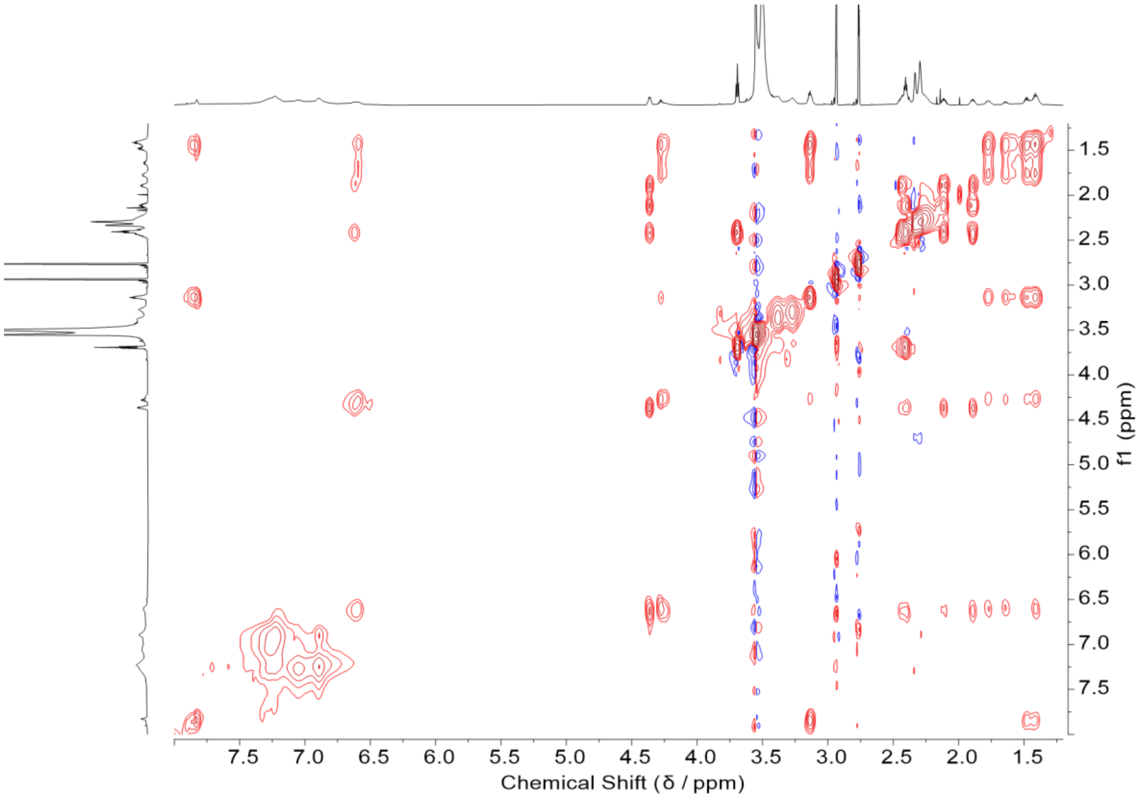

Figure S41: ^1H-^1H-TOCSY NMR spectrum of [Cu(DP^{Tol}-PSMAt)$_2$]$^+$ in DMF-d_7 with DIPEA base.

4. $^{31}P\{^{1}H\}$ NMR spectrum simulations

The $^{31}P\{^{1}H\}$ NMR spectra of *cis*- and *trans*-[$^{nat}ReO_2(DP^{Ph}$-PSMAt$)_2]^+$ and [$^{nat}ReO_2(DP^{Tol}$-PSMAt$)_2]^+$ were simulated in Mestrenova.

First, the spectrum for *cis*-[$^{nat}ReO_2(DP^{Tol}$-PSMAt$)_2]^+$ was simulated as an AA'BB' spin system. Due to *trans*-[$^{nat}ReO_2(DP^{Tol}$-PSMAt$)_2]^+$ showing only 10 of the 24 expected lines for the AA'BB' spin system, the simulation for the *trans* isomer was achieved using the numerical *J* values from the *cis* isomer. For example, the large *trans*-P atom coupling $J_{AB'} = J_{A'B}$ = 360 Hz for the *cis* isomer was used for $J_{AA'}$ and $J_{BB'}$ of the *trans* isomer. In this way, a reasonable simulation fit for the *trans* isomer was achieved, where the observed pattern could only be achieved when $J_{AA'} = J_{BB'}$ = large (> 100 Hz) and the rest < ±20 Hz.

***cis*-[$^{nat}ReO_2(DP^{Tol}$-PSMAt$)_2]^+$: Simulated $^{31}P\{^{1}H\}$ NMR** (283 MHz, D_2O): δ (ppm) 25.35 (m, $J_{AB'} = J_{A'B}$ = 360 Hz, $J_{AB} = J_{A'B'}$ = 15.0 Hz, $J_{AA'}$ = 0.25 Hz), 23.8 (m, $J_{AB'} = J_{A'B}$ = 360 Hz, $J_{AB} = J_{A'B'}$ = 15.0 Hz, $J_{BB'}$ = 0.25 Hz).

***trans*-[$^{nat}ReO_2(DP^{Tol}$-PSMAt$)_2]^+$: Simulated $^{31}P\{^{1}H\}$ NMR** (283 MHz, D_2O): δ (ppm) 26.91 (m, $J_{AA'}$ = 360 Hz, $J_{AB} = J_{A'B'}$ = 15.0 Hz, $J_{AB'} = J_{A'B}$ = 0.25 Hz), 22.12 (m, $J_{BB'}$ = 360 Hz, $J_{AB} = J_{A'B'}$ = 15.0 Hz, $J_{AB'} = J_{A'B}$ = 0.25 Hz)

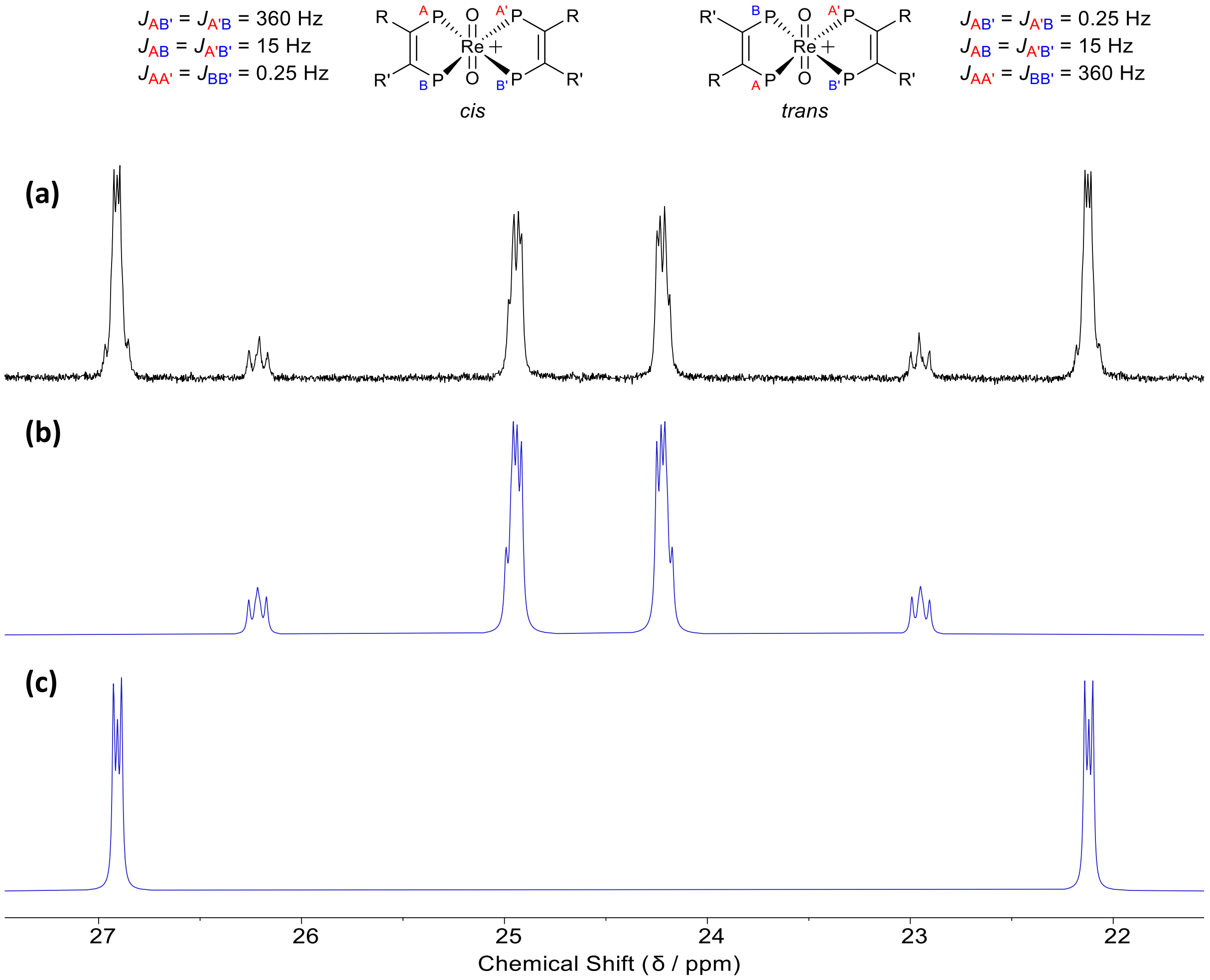

Figure S42: (a) Experimental $^{31}P\{^{1}H\}$ NMR spectrum of cis/trans-[$^{nat}ReO_2(DP^{Tol}$-PSMAt$)_2]^+$. Simulated $^{31}P\{^{1}H\}$ NMR spectrum of (b) cis-[$^{nat}ReO_2(DP^{Tol}$-PSMAt$)_2]^+$ and (c) trans-[$^{nat}ReO_2(DP^{Tol}$-PSMAt$)_2]^+$.

Simulation using the J values for *cis/trans*-[$^{nat}ReO_2(DP^{Tol}$-PSMAt)$_2$]$^+$ also gave a reasonable fit for the analogous *cis*- and *trans*-[$^{nat}ReO_2(DP^{Ph}$-PSMAt)$_2$]$^+$ complexes. Less accurate shifts were obtained due to line broadening in the experimental spectrum of *cis*- and *trans*-[$^{nat}ReO_2(DP^{Ph}$-PSMAt)$_2$]$^+$.

cis-[$^{nat}ReO_2(DP^{Ph}$-PSMAt)$_2$]$^+$: **$^{31}P\{^1H\}$ NMR** (283 MHz, D_2O): δ (ppm) 26.2 (m, $J_{AB'} = J_{A'B}$ = 360 Hz, $J_{AB} = J_{A'B'}$ = 15.0 Hz, $J_{AA'}$ = 0.25 Hz), 24.4 (m, $J_{AB'} = J_{A'B}$ = 360 Hz, $J_{AB} = J_{A'B'}$ = 15.0 Hz, $J_{BB'}$ = 0.25 Hz).

trans-[$^{nat}ReO_2(DP^{Ph}$-PSMAt)$_2$]$^+$: **$^{31}P\{^1H\}$ NMR** (283 MHz, D_2O): δ (ppm) 28.0 (m, $J_{AA'}$ = 360 Hz, $J_{AB} = J_{A'B'}$ = 15.0 Hz, $J_{AB'} = J_{A'B}$ = 0.25 Hz), 22.5 (m, $J_{BB'}$ = 360 Hz, $J_{AB} = J_{A'B'}$ = 15.0 Hz, $J_{AB'} = J_{A'B}$ = 0.25 Hz).

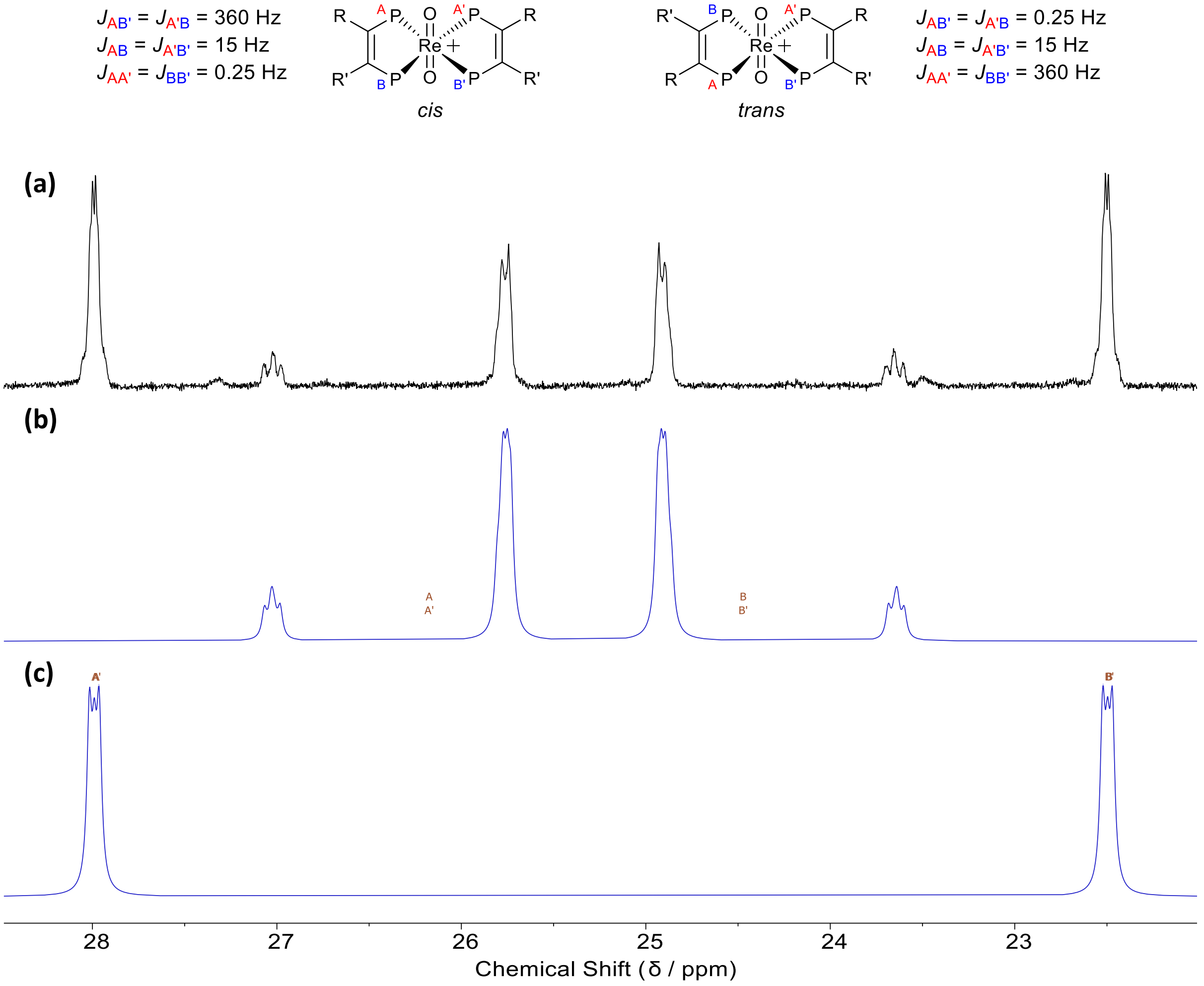

Figure S43: (a) Experimental $^{31}P\{^1H\}$ NMR spectrum of cis/trans-[$^{nat}ReO_2(DP^{Ph}$-PSMAt)$_2$]$^+$. Simulated $^{31}P\{^1H\}$ NMR spectrum of (b) cis-[$^{nat}ReO_2(DP^{Ph}$-PSMAt)$_2$]$^+$ and (c) trans-[$^{nat}ReO_2(DP^{Ph}$-PSMAt)$_2$]$^+$.

5. High resolution mass spectrometry results:

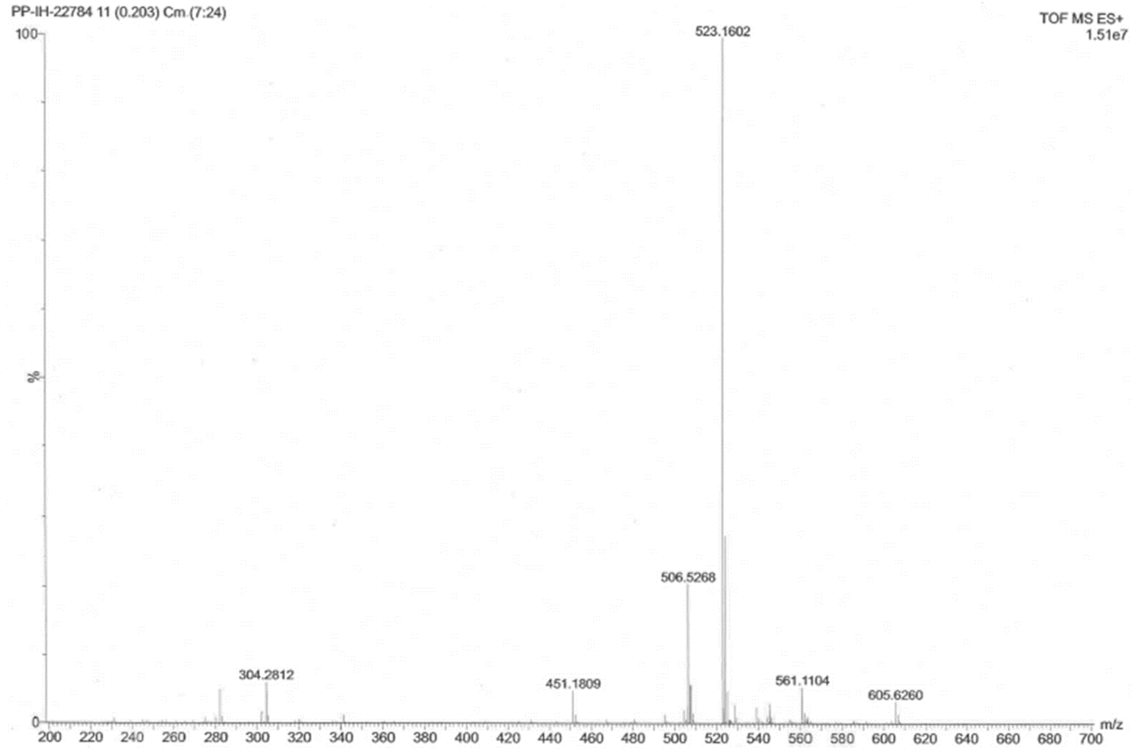

Figure S44: HRMS-ESI+ spectrum of DP^{Tol}.

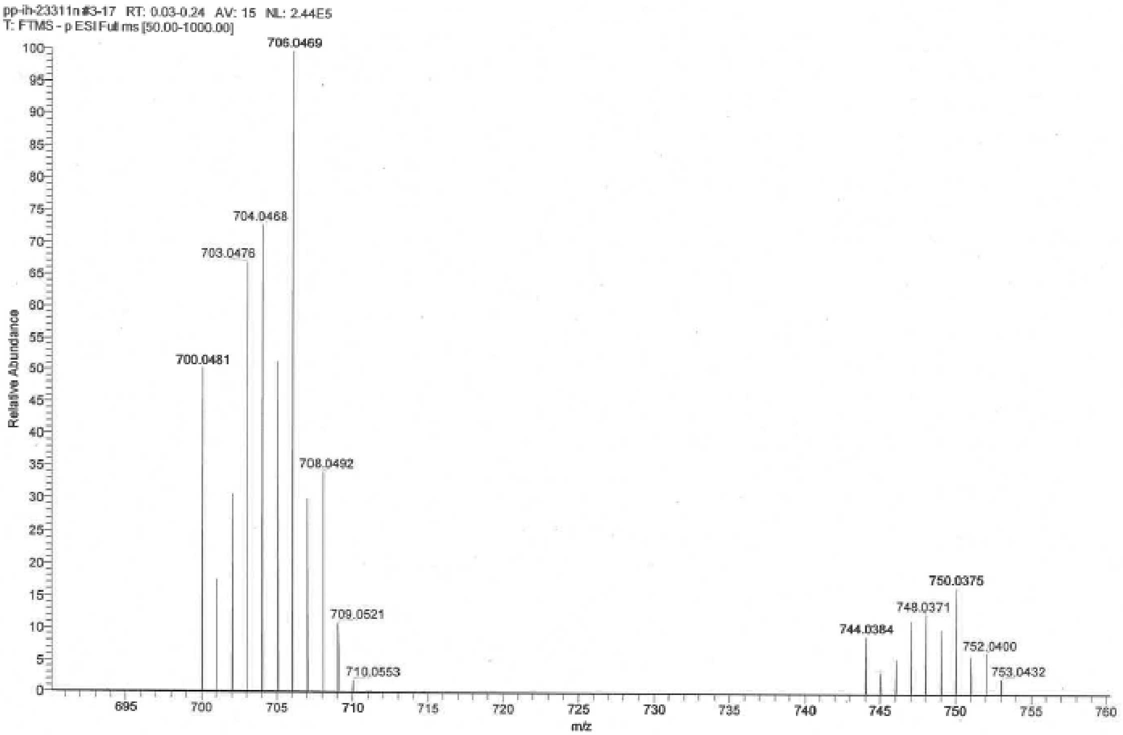

Observed m/z	Theoretical Mass	Error (ppm)	Formula	ID
750.0375	750.0339	4.80	$C_{35}H_{29}NO_8P_2Mo$	$[M-H]^-$
706.0469	706.0440	4.11	$C_{35}H_{29}NO_8P_2Mo$	$[M-H-CO_2]^-$

Figure S45: HRMS-ESI- spectrum of $(RNH_3)[Mo(CO)_4(DP^{Ph}-NHR)]$ ($R = -CH_2CH_2OCH_3$).

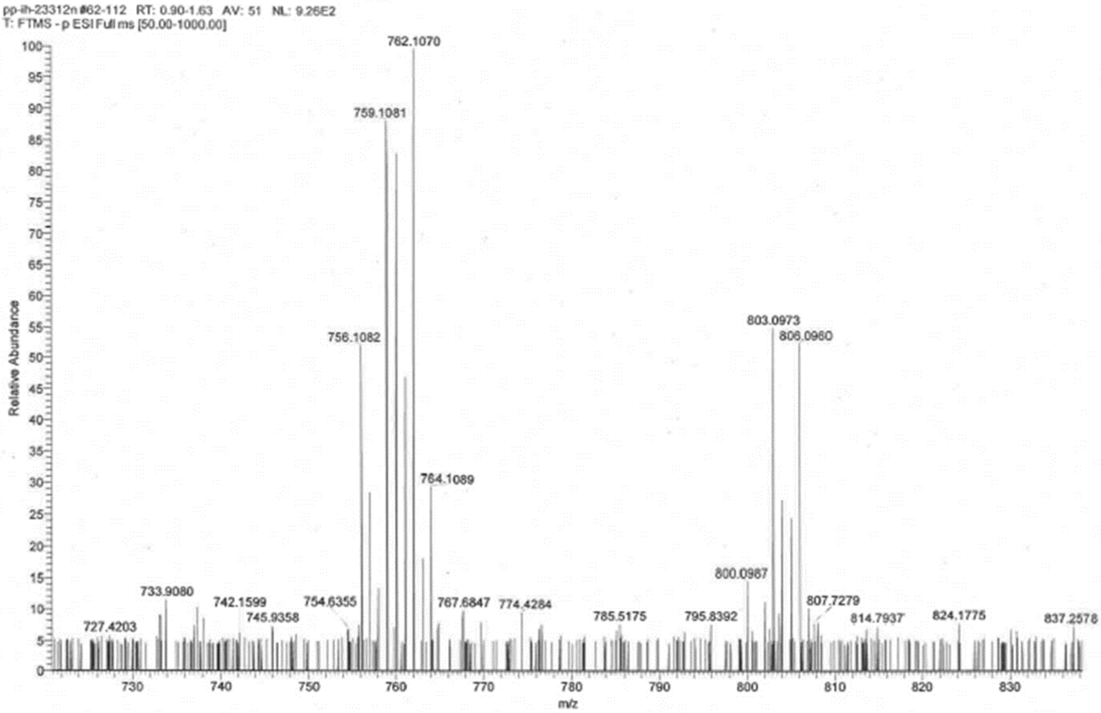

Observed m/z	Theoretical Mass	Error (ppm)	Formula	ID
762.1070	762.1066	0.52	$C_{39}H_{37}NO_8P_2Mo$	$[M\text{-}H\text{-}CO_2]^-$

Figure S46: HRMS-ESI- spectrum of of $(RNH_3)[Mo(CO)_4(DP^{Tol}\text{-}NHR)]$ ($R = \text{-}CH_2CH_2OCH_3$).

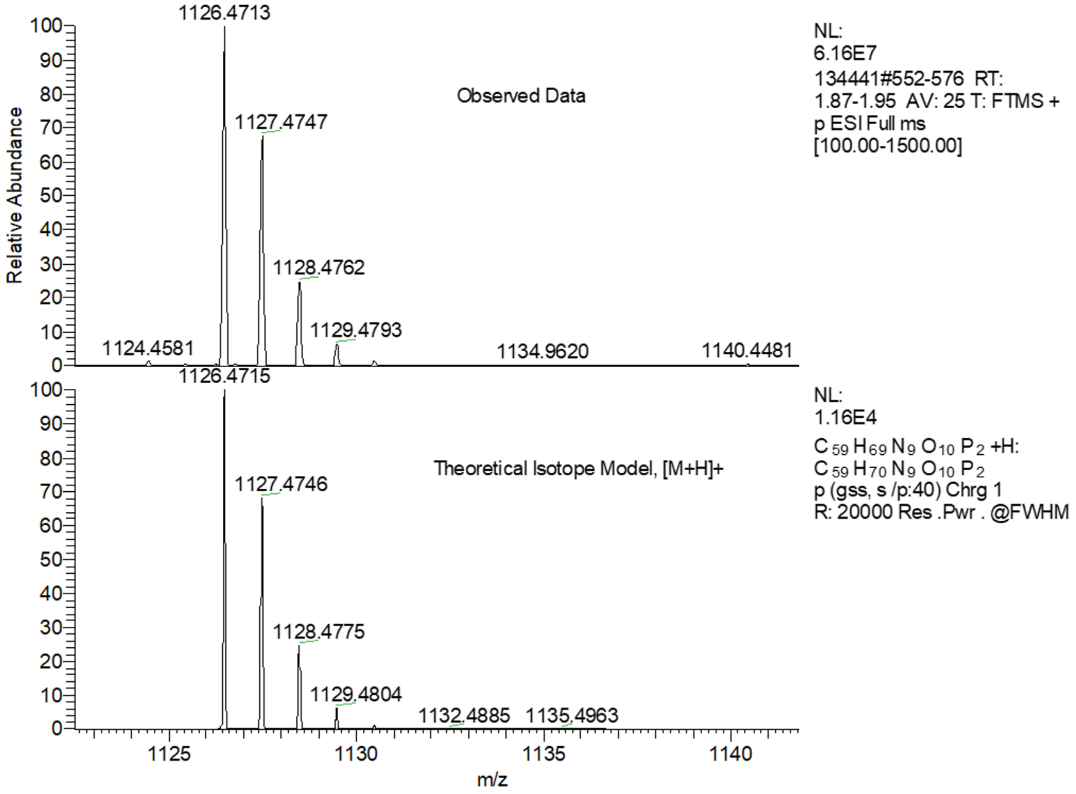

Figure S47: Observed and simulated HRMS-ESI+ spectra of DP^{Tol}-RGD.

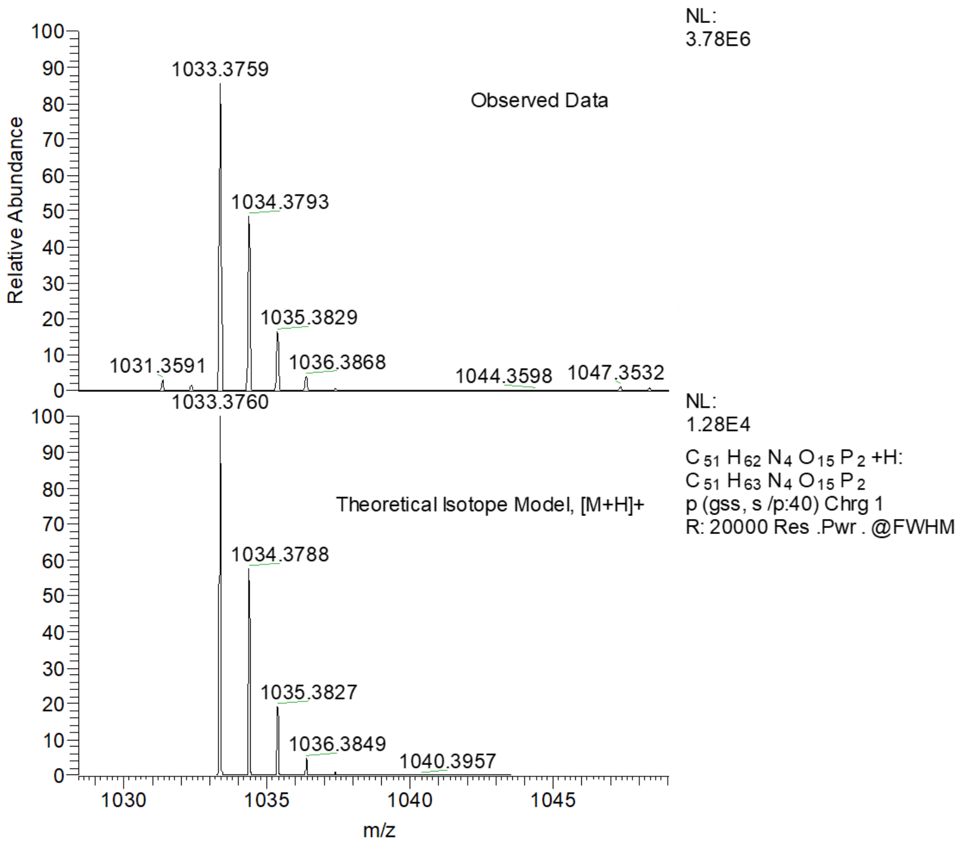

Figure S48: Observed and simulated HRMS-ESI+ spectra of DP^{Ph}-PSMAt.

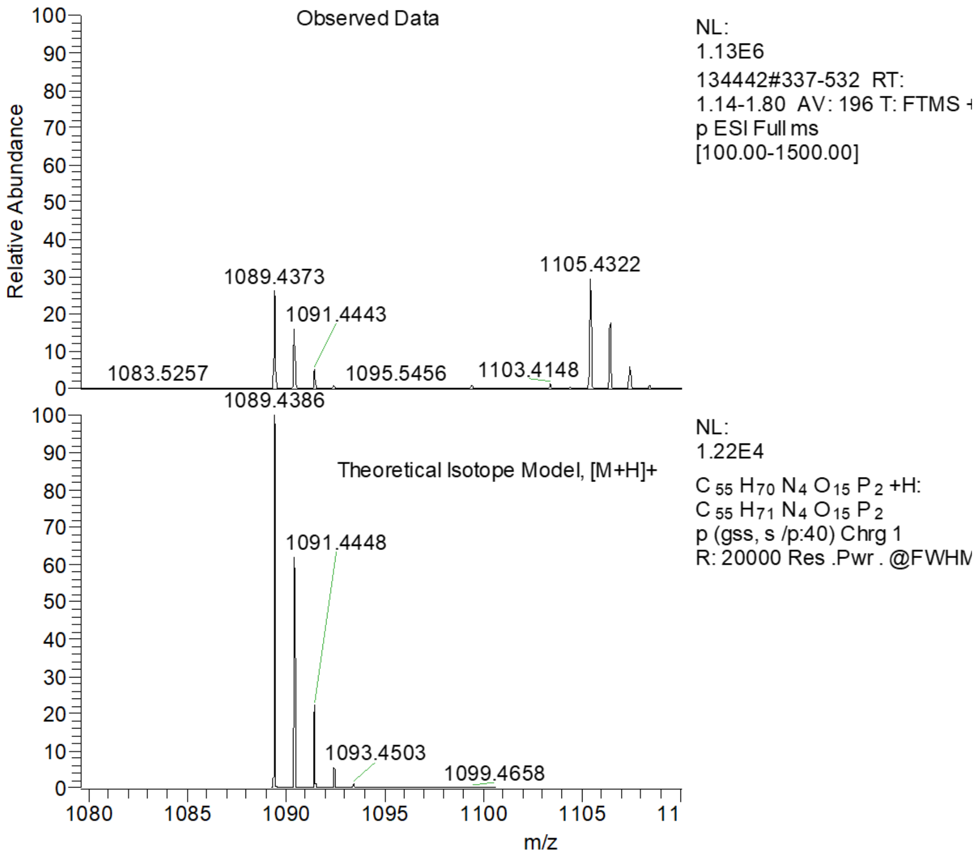

Figure S49: Observed and simulated HRMS-ESI+ spectra of DP^{Tol}-PSMAt.

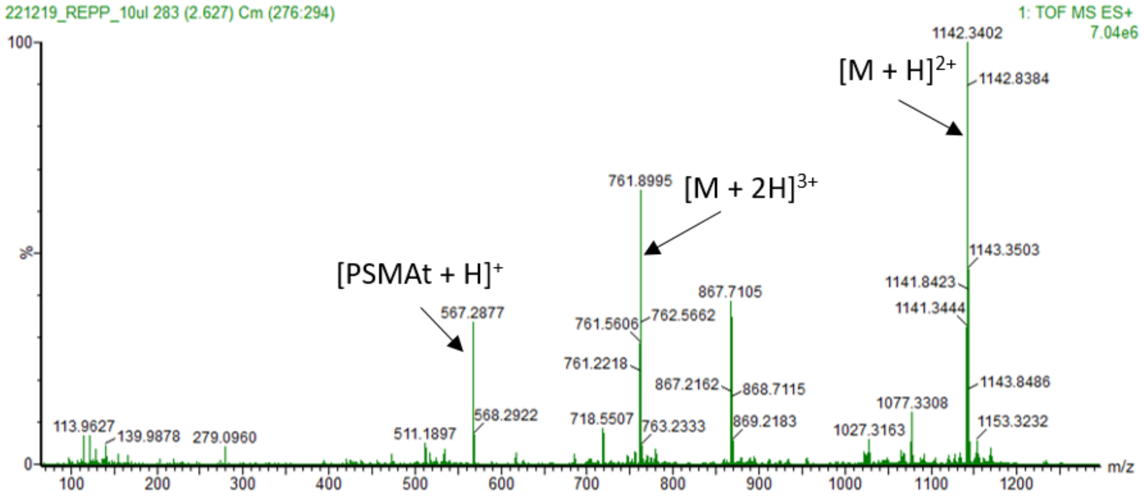

Observed	Theoretical mass	Formula	ID
1142.3402	1142.3454	$C_{102}H_{125}O_{32}N_8P_4Re_1$	$[M+H]^{2+}$
761.8995	761.8993	$C_{102}H_{126}O_{32}N_8P_4Re_1$	$[M+2H]^{3+}$

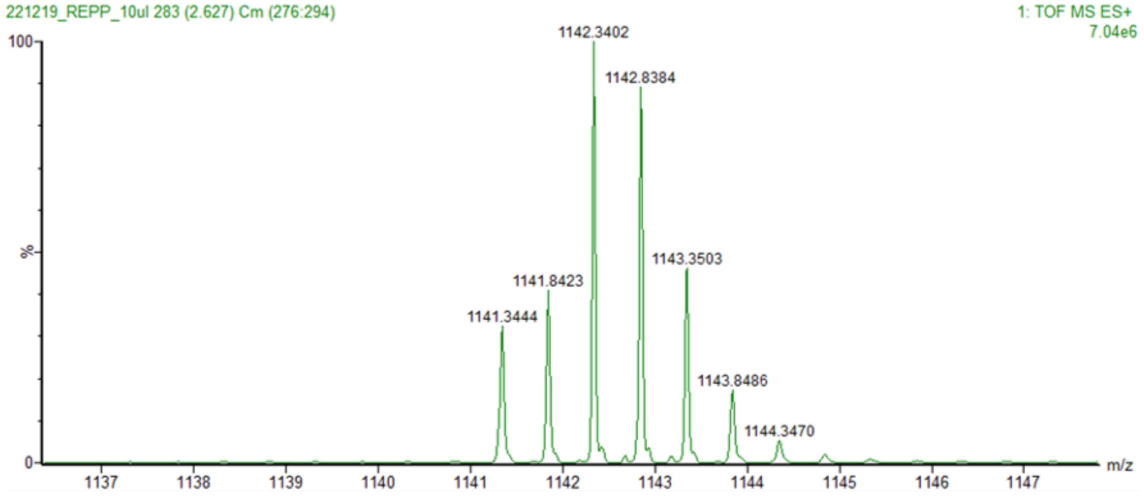

Figure S50: Observed HRMS-ESI+ spectrum of $[ReO_2(DP^{Ph}\text{-}PSMAt)_2]^+$.

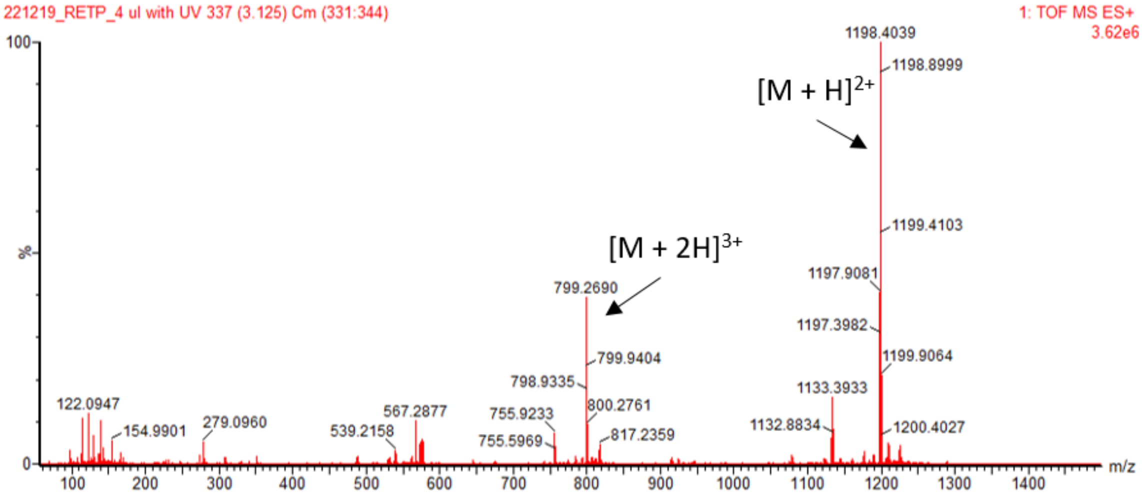

Observed	Theoretical mass	Formula	ID
1198.4039	1198.4080	$C_{110}H_{141}O_{32}N_8P_4Re_1$	$[M+H]^{2+}$
799.2690	799.2744	$C_{110}H_{142}O_{32}N_8P_4Re_1$	$[M+2H]^{3+}$

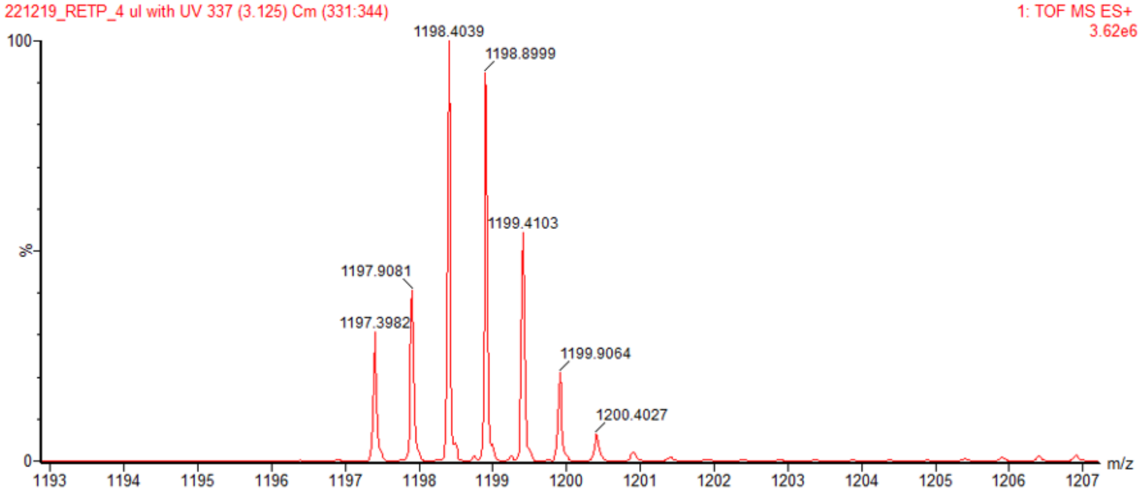

Figure S51: Observed HRMS-ESI+ spectrum of $[ReO_2(DP^{Tol}\text{-}PSMAt)_2]^+$.

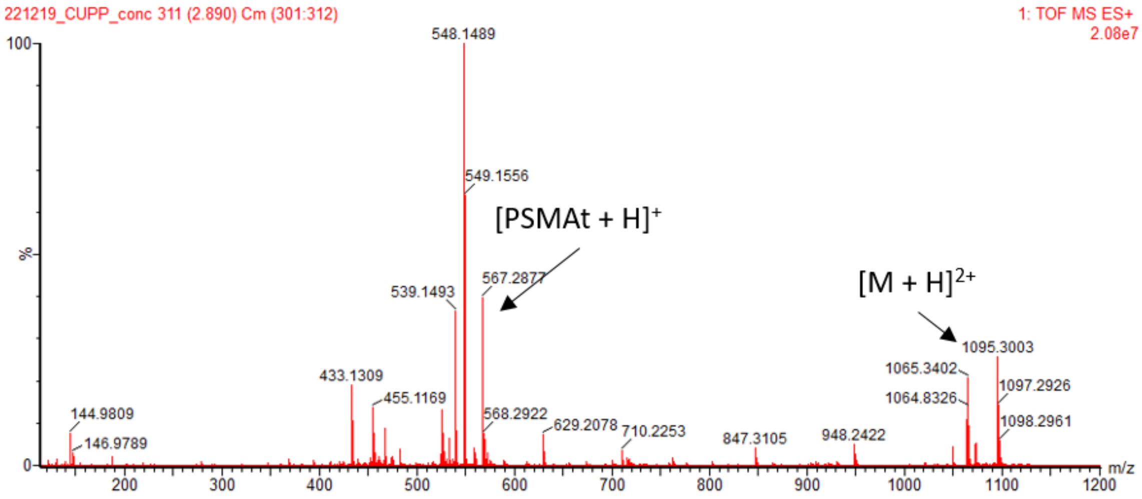

Observed	Theoretical mass	Formula	ID
1065.3402	1065.3385	$C_{102}H_{125}Cu_1N_8O_{30}P_4$	$[M+H]^{2+}$

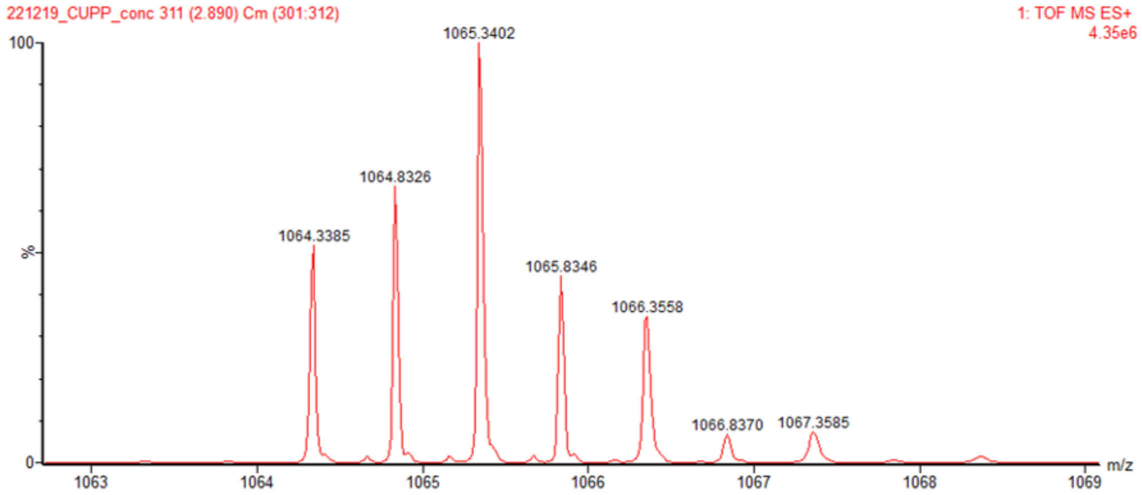

Figure S52: Observed HRMS-ESI+ spectrum of $[CuO_2(DP^{Ph}\text{-}PSMAt)_2]^+$.

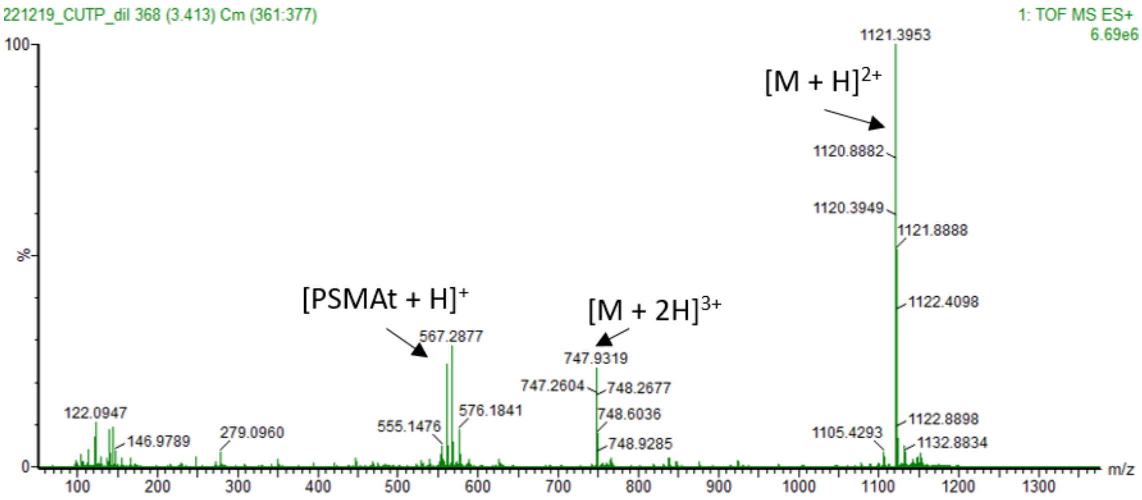

Observed	Theoretical mass	Formula	ID
1121.3953	1121.4012	$C_{110}H_{141}Cu_1N_8O_{30}P_4$	$[M+H]^{2+}$
747.9319	747.9366	$C_{110}H_{142}Cu_1N_8O_{30}P_4$	$[M+2H]^{3+}$

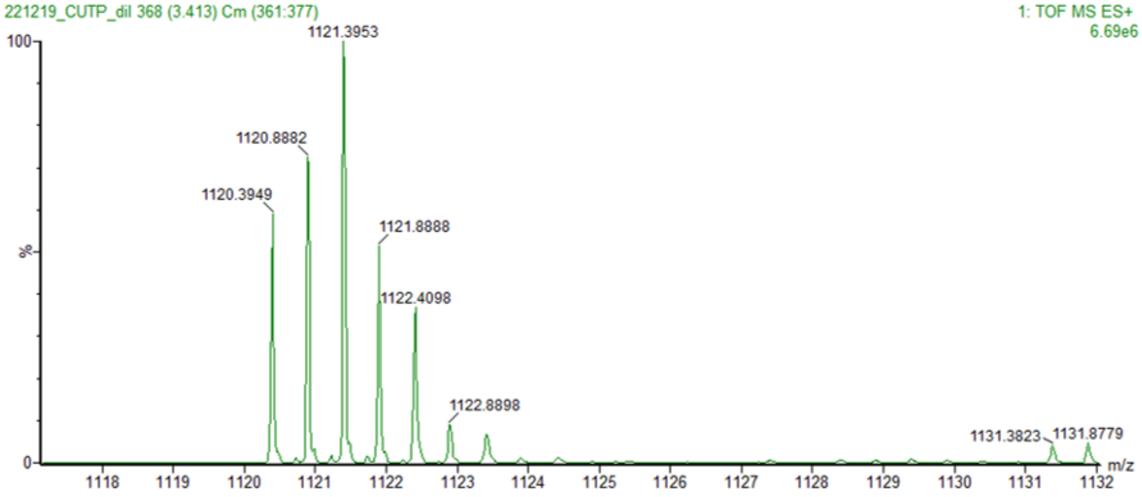

Figure S53: Observed HRMS-ESI+ spectrum of $[CuO_2(DP^{Tol}\text{-}PSMAt)_2]^+$.

6. Infra-red spectroscopy results:

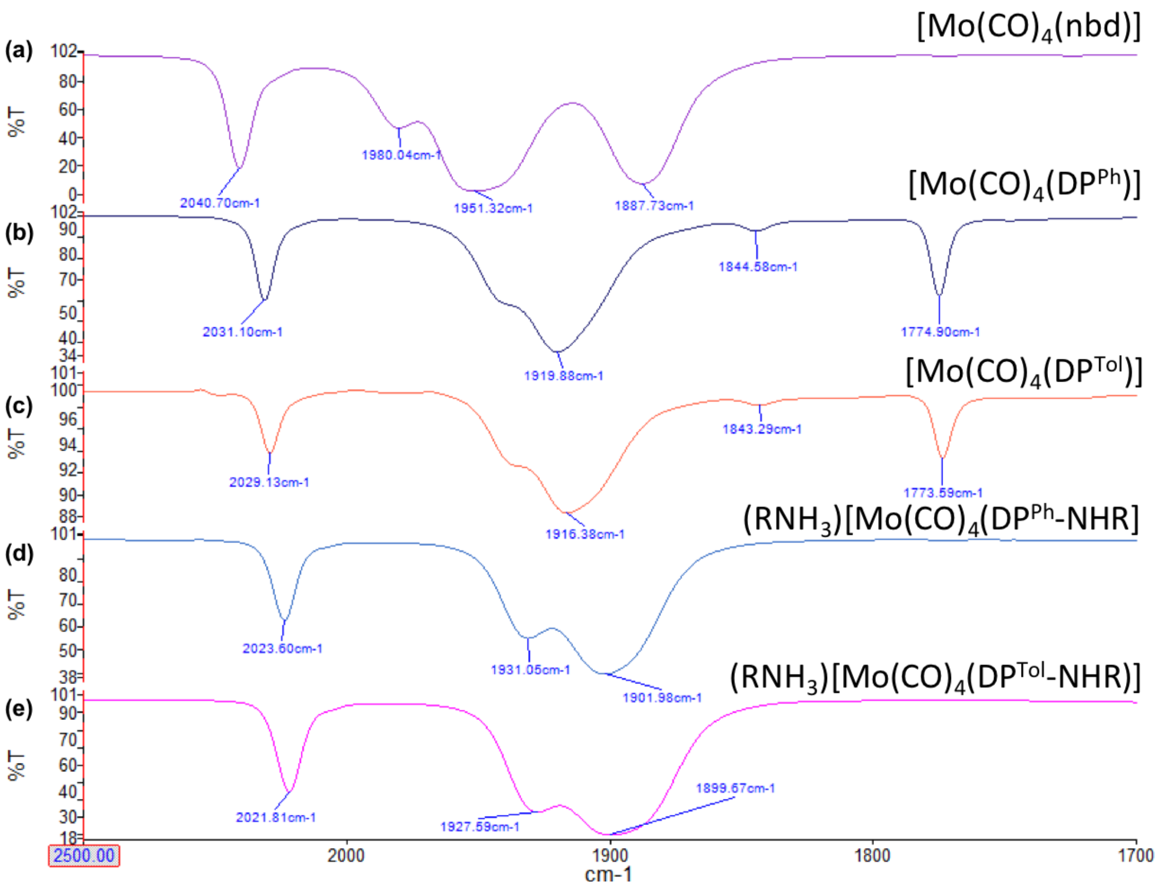

Figure S54: The carbonyl stretching region for M-CO in the infra-red spectra of [Mo(CO)$_4$(nbd)], [Mo(CO)$_4$(DP$^{Ph/Tol}$)], and [Mo(CO)$_4$(DP$^{Ph/Tol}$-NHR)]$^-$. The highest frequency band was used for comparisons. Shoulder peak positions for L = DP^{Ph} and DP^{Tol} are estimated values determined manually.

7. HPLC and low-resolution LCMS-ESI results:

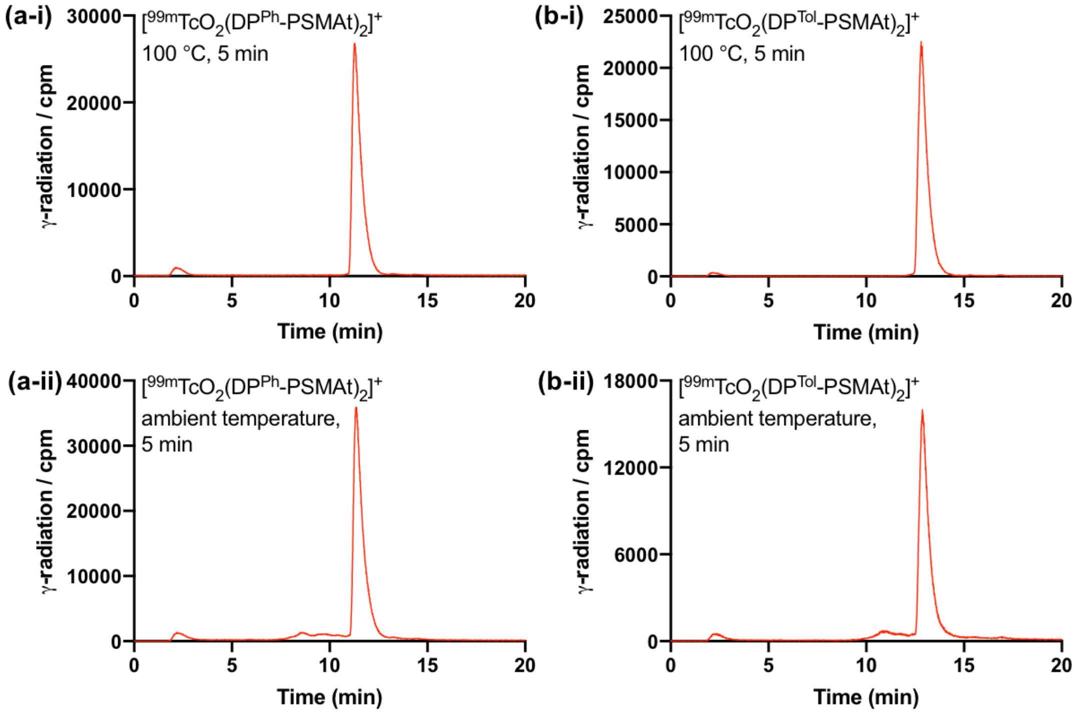

Figure S55: (a) Reverse phase radio-HPLC trace of [^{99m}TcO$_2$(DP^{Ph}-PSMAt)$_2$]$^+$ prepared from an aqueous solution of ^{99m}TcO$_4^-$ and a kit, at either (a-i) 100 °C or (a-ii) ambient temperature; (b) Radio-HPLC trace of [^{99m}TcO$_2$(DP^{Tol}-PSMAt)$_2$]$^+$ prepared from an aqueous solution of ^{99m}TcO$_4^-$ and a kit, at either (b-i) 100 °C or (b-ii) ambient temperature.

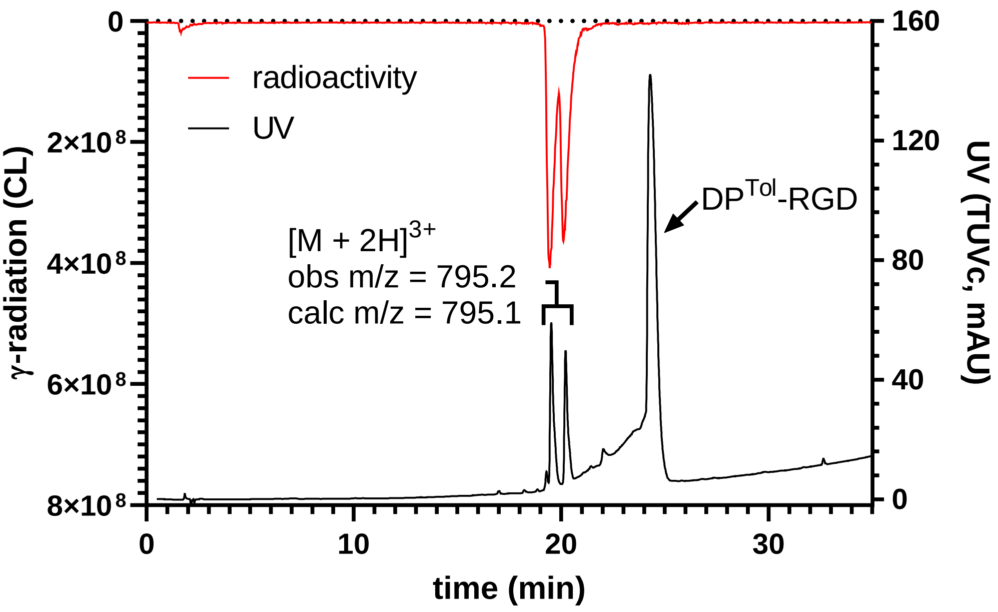

Figure S56: HPLC analysis of crude [$^{99m/99}$TcO$_2$(DP^{Tol}-RGD)$_2$]$^+$. The radiochromatogram (red) shows two species, consistent with separation of the cis- and trans-[$^{99m/99}$Tc][TcO$_2$(DP^{Tol}-RGD)$_2$]$^+$). Two co-eluting peaks were observed in the UV chromatogram (black). The LR-MS-ESI+ spectra of the two co-eluting species both showed signals corresponding to the dipositive and tripositive molecular ions of [TcO$_2$(DP^{Tol}-RGD)$_2$]$^+$.

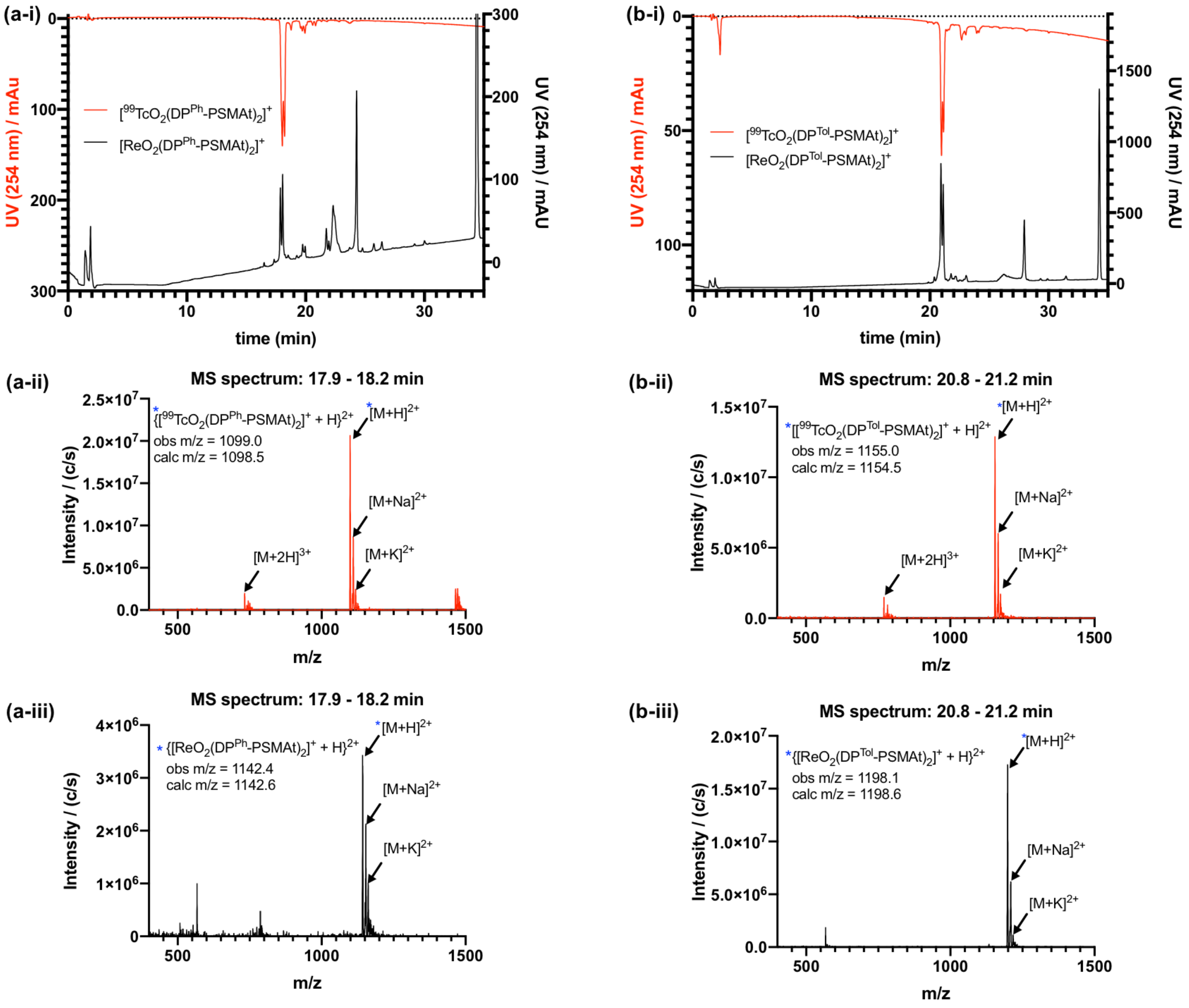

Figure S57: UV chromatogram and mass spectrum of $[MO_2(DP^{Ph}\text{-}PSMAt)_2]^+$) and $[MO_2(DP^{Tol}\text{-}PSMAt)_2]^+$ (M = $^{99g}Tc/^{nat}Re$).

8. References

1 Fenske, D.; Becher, H. J. 2,3-Bis(diphenylphosphino)maleinsäureanhydrid und Diphenylphosphinoderivate des Cyclobutendions als Liganden in Metallcarbonylen. *Chem. Ber.* **1974**, *107*, 117–122.

2 Fei, M.; Sur, S. K.; Tyler, D. R. Reaction of (η_5-C_5Ph_5)$_2$$Mo_2(CO)_6$ with a Chelating Phosphine Ligand: Generation of Stable 17- and 19-Electron Complexes. Dynamic Equilibrium of (η_5-C_5Ph_5)$_2$$Mo_2(CO)_6$ and (η_5-C_5Ph_5)$Mo(CO)_3$. *Organometallics* **1991**, *10*, 419–423.

3 Provis-Evans, C. B.; Emanuelsson, E. A. C.; Webster, R. L.; Rapid Metal-Free Formation of Free Phosphines from Phosphine Oxides. *Adv. Synth. Catal.* **2018**, *360*, 3999–4004.

4 Rong, M. K.; Van Duin, K.; Van Dijk, T.; De Pater, J. J. M.; Deelman, B. J.; Nieger, M.; Ehlers, A. W.; Slootweg , J. C.; Lammertsma, K. Iminophosphanes: Synthesis, Rhodium Complexes, and Ruthenium(II)-Catalyzed Hydration of Nitriles. *Organometallics* **2017**, *36*, 1079–1090.
